# Synaptic plasticity-based regularizer for artificial neural networks

**DOI:** 10.1038/s41598-025-91635-3

**Published:** 2025-04-24

**Authors:** Qais Yousef, Pu Li

**Affiliations:** Group of Process Optimization, Institute for Automation and Systems Engineering, Technische Universität Ilmenau, P.O. Box 100565, 98684 Ilmenau, Germany

**Keywords:** Distribution change, Model regularization, Synaptic plasticity, Variable environment, Computational science, Computer science

## Abstract

Regularization is an important tool for the generalization of ANN models. Due to the lack of constraints, it cannot guarantee that the model will work in a real environment with input data distribution changes. Inspired by neuroplasticity, this paper introduces a bounded regularization method that can be safely applied during the deployment phase. First, the reliability of neuron outputs is improved by extending our recent neuronal masking method to generate new supporting neurons. The model is then regularized by incorporating a synaptic connection module containing conenctions of the generated neurons to their previous layer. These connections are optimized online by introducing a synaptic rewiring process triggered by the information about the input distribution. This process is formulated as bilevel mixed-integer nonlinear programming (MINLP) with an objective to minimize the outer risk of the output by identifying the connections that minimize the inner risk of the neuron output. To address this optimization problem, a single-wave scheme is introduced to decompose the problem into smaller, parallel sub-problems that minimize the inner cost function while ensuring the aggregated solution to minimize the outer one. In addition, a storage/recovery memory module is proposed to memorize these connections and their corresponding risks, enabling the model to retrieve previous knowledge when encountering similar situations. Experimental results from classification and regression tasks show around 8% improvement in accuracy over state-of-the-art techniques. As a result, the proposed regularization method enhances the adaptability and robustness of ANN models in a variable environment.

## Introduction

The training data for an artificial neural network (ANN), regardless of its quantity, cannot encompass all potential variations that may encounter the model in the deployment environment. This limitation arises because the distributions of the real-world input data are variable and, hence, challenging to correctly predict the output during the deployment phase. This issue manifests in various applications, such as autonomous driving and medical imaging. The performance of ANN models in such applications is often limited due to their tendency to overfit and low generalization capability^1–3^. To mitigate this problem, approaches such as cross-validation^4^, dropout^5,6^, and regularization^7^ are usually employed during the training phase of the model by considering the stochastic nature of the training data. However, during the deployment phase, these techniques lose their efficacy as the distribution of the input data changes, because the trained model is deterministic, thereby reducing its ability to process the newly coming samples.

In response to the variable nature of the data distribution in a real environment, termed as a variable environment in our previous work^8^, previous studies have proposed regularization methods to enhance the generalization of the model and mitigate the effect of distributional changes, e.g., Bayesian neural network (BNN)^5,9,10^ and evidential learning^7,11^. Although they have shown promising results, their reliance on prior estimates limits the reliability in a variable environment. Similarly, meta-learning methods^12–14^ address this issue by training the model to adapt to distributional changes. Yet, these methods also require prior estimates. On the other hand, test-time approaches primarily depend on certainty estimates^15–17^ or sample features^18^, offering significant improvements but with high computational costs, thus limiting their real-time application. Meanwhile, low-cost methods that apply regularization over weights^19,20^ or entropy^21^ during the deployment show only modest improvements due to a lack of proper boundedness^5,22,23^, leading to instability and divergence of the model.

To ensure a reliable prediction in a variable environment, an ANN model needs to generalize consistently from the training dataset (with a source distribution), adapt to new events from the deployment environment (with a varying target distribution), and retain its previous knowledge for future use. This behavior mirrors the human brain, primarily due to neuroplasticity, also called synaptic plasticity^24^. Current methods inspired by synaptic plasticity^25–29^ frequently adjust the model parameters through supervised or reinforcement learning principles. Although solutions like^29^ provide lightweight learning abilities, these learning techniques depend to some extent on retraining to adapt to the changed distribution, which does not meet the expectations and requirements of real-world applications.

This study addresses the change of input data distribution within a variable environment by proposing a bounded regularization approach that is inspired by synaptic plasticity. Our aim is to enhance the adaptability of ANN models to distributional changes during deployment. We focus on the structural plasticity^24^ characterized by synaptic rewiring based on new stimuli^24,30^. This systematic rewiring allows neurons to adapt to changing input distributions, producing precise and accurate outputs while retaining existing knowledge for future use. This feature allows for seamless adaptation to new situations, thereby enhancing cognitive abilities^31–34^. Consequently, the brain makes transitions from slow, logical, rational, and conscious thinking to fast, almost instantaneous, automatic, and intuitive thinking^35^.

Based on this natural synaptic plasticity principle, we hypothesize that neuronal connection restructuring during the deployment of an ANN model, in a bounded manner, can enhance its adaptability and consequently its accuracy. We realize this by extending our previously proposed neuronal masking method^36^ to apply to any layer of the neural network except the input layer. Originally, neuronal masking involved generating new masks (neurons) for the output logits and connecting them randomly to neurons from the previous layer. This allows the newly generated masks to produce outputs slightly different from that of the original logit. By providing more relevant alternatives, the generated masks contribute to enhancing the model’s accuracy. Due to the lack of labels, during the model deployment, the accuracy is estimated by measuring the prospect certainty of the model output^36^.

In this study, after extending the neuronal masking method to other layers of the model, we introduce the concept of synaptic connections, which includes the structure of the neuronal connections for the introduced masks. We then propose a synaptic rewiring process to optimize the connection structure online by minimizing the prospect risk of the model output, i.e. maximizing the prospect certainty. The optimization problem is defined as a mixed-integer nonlinear programming (MINLP). Given the high dimensionality of the problem which includes the synaptic connections for all the generated masks for the neurons within the selected layers, we treat the synaptic rewiring process by introducing a single wave scheme. This scheme utilizes agents of evolutionary algorithms simultaneously to solve the MINLP for all the masks within the selected layers by minimizing the risk of the output of their corresponding neurons such that the overall prospect risk of the model output is minimized.

In addition, given the complexity of the bounded synaptic rewiring process, we employ the concept of the information source proposed in^8^ to provide a quantified value representing the input distribution. This allows the evaluation of the degree of distributional change of the model input to trigger the rewiring process only when the distribution change exceeds a threshold. Furthermore, we introduce a storage/retrieve memory to store the synaptic connections that correspond to specific information source values. This enables the model to memorize its experiences for future use with similar input data distributions.

Briefly, by introducing the proposed synaptic plasticity method, we aim to provide a robust and adaptable model that can effectively respond to data distribution changes during deployment in a variable environment, without the need for retraining. To evaluate our approach in variable environments with distributional changes, we provide a mathematical analysis and conduct numerical experiments on classification and regression tasks using benchmark as well as real datasets. The results demonstrate that the proposed approach significantly surpasses existing techniques in terms of accuracy.

The remainder of the paper is as follows. Sect. "Methodology development" formulates the problem understudy, provides the necessary preliminary background, and elaborates on the solution in detail. In Sect. "Implementation and Experimental Results", we present our evaluation procedure, illustrate the experimental results, and discuss their implications. Finally, in Sect."Conclusions", we conclude our work and propose potential future studies.

## Methodology development

In this section, we present the development of our method. We begin with the general form of an ANN model, and then we proceed to formulate the specific problem of this study, highlighting the challenges and requirements. Finally, we detail our proposed solution, elucidating how it addresses the identified problem and enhances the adaptability of the model.

### Code availability

A corresponding code that demonstrates the implementation of the components in the proposed synaptic plasticity method is provided in^37^ illustrating a deeper understanding of the implementation of our approach.

### Mathematical formulation of ANN model

A general form of an ANN model $\:f$ can be expressed as^38^

1 $$\:{\widehat{\varvec{x}}}_{t}=f({\varvec{u}}_{t},\varvec{\theta\:})$$

where $\:{\widehat{\varvec{x}}}_{t}$ is the output logit vector at sample time $t \in \left\{ {t_{1} ,t_{2} , \ldots ,t_{T} } \right\}$, $\:{\varvec{u}}_{t}$ is the input vector, and $\:\varvec{\theta\:}\:$is the parameter set. The output of this model is described as

2 $$\:{\widehat{\varvec{y}}}_{t}=g\left({\widehat{\varvec{x}}}_{t}\right)$$

where $\:g(.)$ is an activation function.

### Problem statement

It is commonly known that the objective of training is to minimize the empirical risk using the available data with a source distribution $\:{\mathcal{P}}^{s}$, i.e^23,38^.

3 $$\:{\mathfrak{R}}_{emp}\left({\mathcal{P}}^{s},\varvec{\theta\:},\mathcal{L}\left(\widehat{\varvec{y}},{\varvec{u}}^{s},\varvec{\theta\:}\right)\right)=\frac{1}{n}\sum\:_{i=1}^{n}\mathcal{L}\left({\widehat{\varvec{y}}}_{i},{\varvec{u}}_{i}^{s},\varvec{\theta\:}\right)$$

where$\:\mathcal{\:}\mathcal{L}(.)$ is the loss function. Since it depends on the source distribution $\:{\mathcal{P}}^{s}$, $\:{\mathfrak{R}}_{emp}$ can easily tend to overfit. Therefore, to reduce overfitting during the training pipeline, a regularized risk $\:{\mathfrak{R}}_{reg}$ is to be minimized, after adding a regularizer $\:\varLambda\:\left(\varvec{\theta\:}\right)$ to (3):

4 $$\:\varLambda\:\left(\varvec{\theta\:}\right)=\frac{{\mathcal{P}}^{t}}{{\mathcal{P}}^{s}}$$

which results in^23^,

5 $$\:{\mathfrak{R}}_{reg}\left({\mathcal{P}}^{s},\varvec{\theta\:},\mathcal{L}\left(\widehat{\varvec{y}},{\varvec{u}}^{\varvec{s}},\varvec{\theta\:}\right)\right)={\mathfrak{R}}_{emp}+\gamma\:\varLambda\:\left(\varvec{\theta\:}\right)$$

where $\:\gamma\:$ is a regularization coefficient that tunes the impact of the regularizer. It is well-known that regularization techniques achieve limited improvement, mainly due to the strength of variations of the target distribution $\:{\mathcal{P}}^{t}$, which cannot be estimated or compensated by the regularizer $\:\varLambda\:\left(\varvec{\theta\:}\right)$ beforehand. Estimating $\:{\mathcal{P}}^{t}$ in a variable environment requires a highly complex process and expensive computational costs. Furthermore, a modest estimation error may result in a very large $\:\varLambda\:$ and lead to a diverged model (refer to^23^ Sect. 8.2).

Therefore, conducting regularization during the deployment period by considering the variations of $\:{\mathcal{P}}^{t}$ could be a valid direction to tackle this dilemma. However, it is unsafe to use an unconstrained or a random form of the regularizer^3^. This is because it will allow additional noise and higher uncertainty to be added to the estimation unless the model is deployed multiple times on the same sample and an average output is taken, as in^5,22^, which however is computationally extremely inefficient. To address this problem, we propose a bounded regularization method, inspired by synaptic plasticity, which maintains the model stability and thus can be safely activated during the deployment phase.

### Proposed approach

The proposed synaptic plasticity-based method consists of three parts (components): (i) neuronal masking and refinement of the neuron output, (ii) synaptic rewiring to determine the synaptic connections for the generated masks triggered by the information about the distribution of the input data, and (iii) storage/retrieval memory to store the experienced results for future use.

#### Neuronal masking and output refinement

##### Neuronal masking

In a recent work^36^, we introduced the concept of neuronal masking which entails generating masks $\left\{ m_{{i,1}} ,m_{{i,2}} , \ldots ,m_{{i,N^{M}}} \right\}$ for the output logit $\:{x}_{i}$ accompanied with specific random connection ratios $R = \left\{ {r_{{i,1}} ,r_{{i,2}} , \ldots ,r_{{i,N^{M} }} } \right\}$. To enhance the probabilistic nature of a trained model, these ratios prevent the masks from being fully connected with the neurons of the previous layer. The fundamental idea is to generate multiple output alternatives for the original neuron. By measuring the mean output of these masks and comparing it to the output of the original neuron, we can estimate the risk of this output and refine it accordingly. The number of masks and the corresponding ratios are defined as hyperparameters (for details see^36^).

In this study, we seek to further enhance the learning capacity of ANN models by focusing on the neuron level and improving its accuracy and certainty. For this purpose, we extend the neuronal masking to apply it to any layer of the model except the input layer.

Let the model $\:f$ have $\:L$ layers in addition to the input layer, let $\:{x}_{l,i}$ be the $\:{i}^{th}$neuron in layer $\:l$, where $\:{\widehat{x}}_{l,i,t}$is its output at sample time $\:t$. Then, its correspondingly generated masks can be represented as,

6 $$M_{{l,i}} = \left\{{m_{l,i,1}, m_{l,i,2}, \ldots,m_{l,i,N_{l}^{M}}} \right\}$$

whose outputs are

7 $$\hat{M}_{{l,i,t}} = \left\{{\hat{m}_{l,i,1,t}, \hat{m}_{l,i,2,t}, ...,\hat{m}_{l,i,N_{l}^{M} ,t}} \right\}$$

**Fig. 1:**
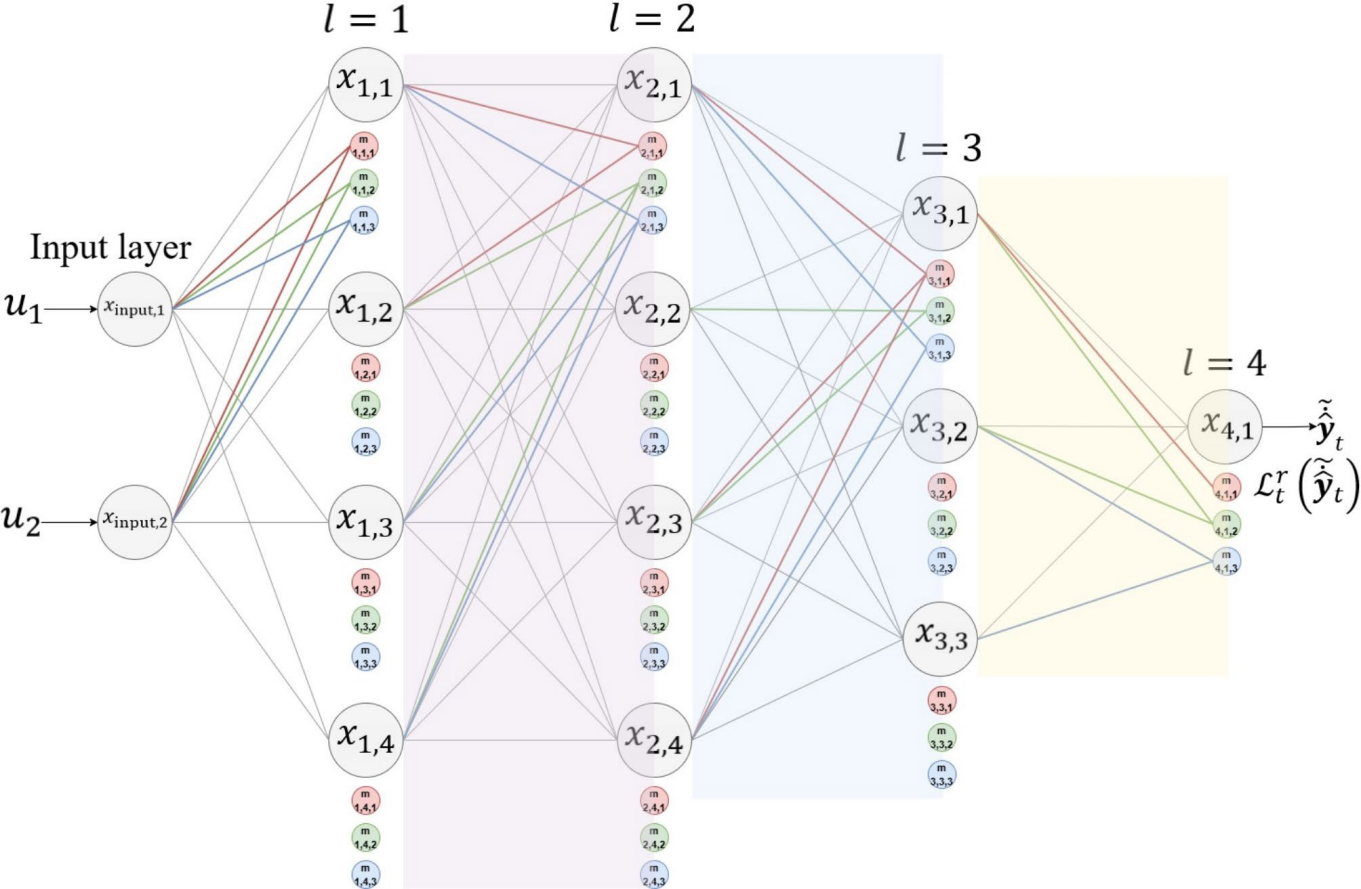
Visualization of the generated masks for each neuron within each layer. In this example, $\:{\left\{{N}_{l}^{M}\right\}}_{l=1}^{L}= \text 3$, indicating that three masks are generated for each neuron. For clarity, only sampled masks and their variable synaptic connections are shown. According to our method, all generated masks should maintain their connections with the neurons of the preceding layer as determined by the single-wave optimization $\:\varvec{S}_{t}^{\text{*}}$. e.g., for the mask $\:{m}_{\text{3,1},3}$, the connection will be $\:{S}_{\text{3,1},3,t}^{\text{*}}=\left\{\text{1,0},\text{0,1}\right\}$.

The selection of the layer(s) for applying neuronal masking is a hyperparameter by specifying the number of masks $\:{N}_{l}^{M}$ to be generated for each neuron in a given layer. This is represented in the vector $\:{\left\{{N}_{l}^{M}\right\}}_{l=1}^{L}$, where non-zero entries indicate the indices of the layers where neuronal masking will be applied, as well as the number of masks to be generated for each neuron in these layers. For instance, consider a model with $\:L=4$ layers. If $\:{\left\{{N}_{l}^{M}\right\}}_{l=1}^{L}=\left\{\text{3,0},\text{5,4}\right\}$, it indicates that the neuronal masking will be applied to layers 1, 3, and 4, with 3, 5, and 4 masks per neuron, respectively. Figure 1 illustrates the concept of a neuronal masking.

As depicted in Fig. 1, each mask receives input through distinct pathways connected to the neurons of its previous layer in varying synaptic connections, which will be detailed in subsection Synaptic Rewiring This approach allows the masks to produce diverse outputs utilized to refine the value of the corresponding original neuron and to estimate its risk.

##### Original neuron output refinement

**Fig. 2:**
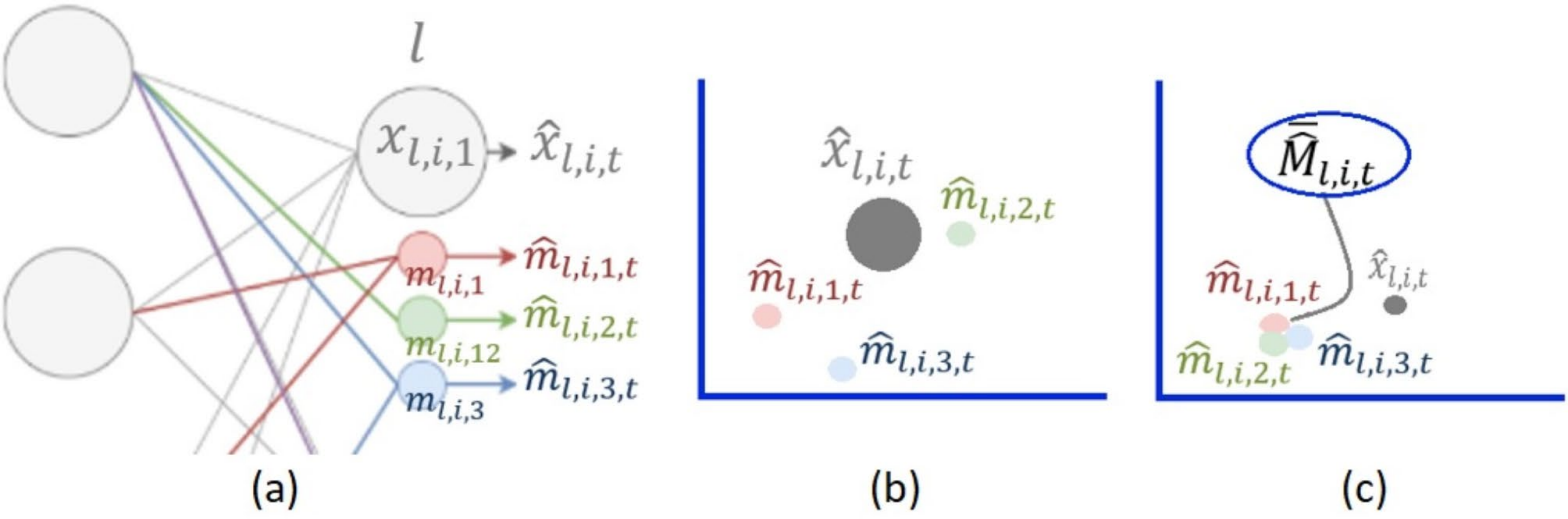
Illustrative example showing the disparity $\:{\sigma\:}_{{M}_{l,i,t}}$ between the original neuron and its masks. (**a**) the generated masks, (**b**) with high disparity, and (**c**) with low disparity.

To obtain an accurate and representative refinement for $\:{\widehat{x}}_{l,i,t}$, we employ the weighted probability function ${\widehat{Pr}} ^\mathcalligra{w}$ proposed in^36^, which represents the certainty of the outputs of the original neuron and each of its masks. This function, as proven in^36^, ensures the satisfaction of the following four conditions to achieve an accurate refinement of $\:{\widehat{x}}_{l,i,t}$ with the value that has the highest weighted probability. If $\:{d}_{l,i,t}=|{\widehat{x}}_{l,i,t}-{\overline{\widehat{M}}}_{l,i,t}|$ is the distance between the original neuron and the mean of its masks, and $\:{\sigma\:}_{{M}_{l,i,t}}$is the standard deviation of these masks, reflecting their disparity, as illustrated in Fig. 2, then the calculation of $\:{\widehat{Pr}}^{\mathcalligra{w}}$ for the values of the original neuron $\:{\widehat{x}}_{l,i,t}$ and each of its masks $\:{\widehat{M}}_{l,i,t}$ satisfies the following conditions:

i. If $\:{\sigma\:}_{{M}_{l,i,t}}<{d}_{l,i,t}$ i.e. the masks are more sure of their outputs than the original neuron, then $\:{\widehat{Pr}}^{\mathcalligra{w}}\left({\widehat{M}}_{l,i,t}\right)>{\widehat{Pr}}^{\mathcalligra{w}}\left({\widehat{x}}_{l,i,t}\:\right)$.

ii. The weights for each mask should reflect its distance from the mean $\:{\overline{\widehat{M}}}_{l,i,t}$.

iii. If $\:{\widehat{x}}_{l,i,t}={\overline{\widehat{M}}}_{l,i,t}={\widehat{m}}_{l,i,j,t}$, then it should be $\:{\widehat{Pr}}^{\mathcalligra{w}}\left({\widehat{x}}_{l,i,t}\:\right)>{\widehat{Pr}}^{\mathcalligra{w}}\left({\widehat{m}}_{l,i,j,t}\right)$, since the original neuron is fully connected with the previous layer,

iv. $$\:{\widehat{Pr}}^{\mathcalligra{w}}\left({\widehat{x}}_{l,i,t}\:\right)\:+\:\sum\:{\widehat{Pr}}^{\mathcalligra{w}}\left({\widehat{M}}_{l,i,t}\right)=1.$$

Hence, $\:{\dot{\widehat{x}}}_{l,i,t}$is refined by selecting the value that has the highest $\:{\widehat{Pr}}^{\mathcalligra{w}}$ among this neuron or any of its masks, formulated as,

8 $$\:{\dot{\widehat{x}}}_{l,i,t}=\text{a}\text{r}\text{g}\text{m}\text{a}\text{x}\left({\widehat{Pr}}^{\mathcalligra{w}}\left({\widehat{x}}_{l,i,t}\:\right),{\left\{{\widehat{Pr}}^{\mathcalligra{w}}\left({\widehat{m}}_{l,i,j,t}\right)\right\}}_{j=1}^{{N}_{l}^{M}}\right)$$

#### Synaptic rewiring

To enable the mask connections to adapt to changes in the input distribution, we dynamically optimize the ratio $\:R$ for the generated masks online, moving away from its constant nature as presented in^36^. Thus, we introduce the concept of the *synaptic connection* $\:S\in\:\varvec{S}$, defining the connections between the masks and the neurons in the previous layer as follows,

9 $$\:{S}_{l,i,j,t}=\left\{{s}_{l,i,j,t,1},{s}_{l,i,j,t,2},\dots\:,{s}_{l,i,j,t,{N}_{l-1}^{N}}\right\},\:\:\forall\:\:2\le\:l\le\:L\:\:;\:s\in\:\left\{\begin{array}{c}0,\:\:\:\:\:\:\:\:\:not\:selected\\\:1,\:\:\:\:\:\:\:\:\:\:\:\:\:\:\:\:\:selected\end{array}\right.$$

$$\:s.t.\:\:\:{N}_{l-1}^{N}>\sum\:{S}_{l,i,j,t}>0$$

Here, $\:{S}_{l,i,j,t}$ is the synaptic connection vector for the mask $\:{m}_{l,i,j}$ at time $\:t$. $\:{N}_{l-1}^{N}$ represents the number of neurons in layer $\:l-1$. $\:{S}_{l,i,j,t}$ contains the indices for all the neurons within $\:l-1$, providing all possible synaptic connections between $\:{m}_{l,i,j}$ and $\:l-1$, such that, the indices of the neurons of $\:l-1$ to which this mask should be connected are set to 1. Synaptic connections are applied to all the masks within any layer $\:2\le\:l\le\:L$, excluding the first layer after the input layer, $\:l=1$. The masks of the first layer are all fully connected with the input layer to ensure that all the input features are considered. The constraint $\:{N}_{l-1}^{N}>\sum\:{S}_{l,i,j,t}>0\:$ensures that each mask has at least one connection to the previous layer without being fully connected. Figure 1 illustrates the synaptic connections for sampled masks.

To ensure boundedness, the synaptic rewiring of the synaptic connections $\:\varvec{S}$ is formulated as a mixed-integer nonlinear programming (MINLP) problem to find optimal connections, by minimizing the prospect risk of the model output.

##### Prospect risk loss

During the deployment phase of the model, calculating the accuracy of its output is challenging due to the lack of available labels. Therefore in^36^, a method is provided to estimate the output accuracy by evaluating its certainty $\:{\Omega\:}\left(\widehat{y}\right)$ based on the prospect theory^39^, as follows,

10 $$\:\varOmega\:\left(\widehat{y}\right)={\varOmega\:}^{b}\left(b\left(\widehat{y}\right)\right)\:{\varOmega\:}^{\mathcalligra{w}}\left({\widehat{Pr}}^{\mathcalligra{w}}\left(\widehat{y}\right)\right)$$

where, $\:{\varOmega\:}^{b}$ is the value function explaining the relative perception of the positive or negative influence of the neuron output. $\:b$ is the behavior function reflecting the neuron’s influence on the distribution of the model output, while $\:{\varOmega\:}^{\mathcalligra{w}}$ is the probability function mirroring the perceived weighted probability $\:{\widehat{Pr}}^{\mathcalligra{w}}$of the influence of this neuron, (see^36^ for details). To be defined as a cost function for the MINLP problem, we reformulate the certainty function in (10) into *prospect risk loss*,

11 $$\:{\mathcal{L}}^{r}=-\varOmega\:$$

$\:{\mathcal{L}}^{r}$ quantifies the risk of the refined output of the model without affecting its neurons or output calculation processes. Bounding the synaptic rewiring problem with the prospect risk ensures the optimal synaptic structure for the masks, such that the overall risk of the model output is minimized. Given the solution’s dimensionality for all the masks across the selected layers $\:\left[\varvec{S}\right]=\sum\:_{l=2}^{L}{N}_{l}^{N}{N}_{l-1}^{N}{N}_{l}^{M}$, and the dependency of each layer’s connections on preceding layers, determining all synaptic connections $\:{\varvec{S}}_{t}^{\text{*}}$ online is challenging. Methods like Bellman’s principle of optimality^40^ could be useful because it divides the synaptic rewiring problem into subproblems based on stages. However, since it requires forward and backward iterations, it will be prohibitive to be used online. Therefore, we propose an online optimization method coined *single-wave* to address the synaptic rewiring problem in a single forward pass.

##### Single wave scheme for optimizing the synaptic rewiring

The single-wave scheme minimizes the cost function (11) by minimizing the internal cost function at each layer. Within each layer, the synaptic connections of the masks for each neuron are optimized simultaneously using an evolutionary algorithm, such as the dwarf mongoose algorithm^41^. This is achieved by assigning a search agent to each neuron to determine the optimal synaptic connection vector $\:{\varvec{S}}_{l,i,t}^{*}$ for its masks by minimizing their internal cost function.

We design the internal cost function to assess the risk of the refined neuron. From (8), the neuron’s refinement is achieved by selecting the output with the highest weighted probability among the neuron and its masks. Therefore, $\:{\dot{\widehat{\varvec{x}}}}_{l,i,t}$ directly depends on its masks. Optimizing the synaptic connections of the masks allows them to produce more accurate and certain values, thereby refining $\:{\dot{\widehat{\varvec{x}}}}_{l,i,t}$. Consequently, the optimization of the synaptic connections for the masks can be achieved by minimizing the risk of the refined neuron. This risk can be measured by converting the weighted probability $\:{\widehat{Pr}}^{\mathcalligra{w}}\left({\dot{\widehat{\varvec{x}}}}_{l,i,t}\right)$ to a convex form $\:1-{\widehat{Pr}}^{\mathcalligra{w}}$.

Given the dependency of each layer on the preceding layers, the internal cost function at the neuron level must also account for the accumulated risk from the previous layers. To formulate the cost functions and the optimization problem for synaptic rewiring using the proposed single-wave scheme, it is assumed that all layers, except the input layer, deploy synaptic plasticity features. Thus, the internal cost function is formulated as follows,

12 $$\:{\xi\:}_{l,i,t}\left({\varvec{M}}_{l,i}\left({\varvec{S}}_{l,i,t}.\:{\stackrel{\sim}{\dot{\widehat{\varvec{x}}}}}_{l-1,t}\right),{\delta\:}_{l}\right)=\left(1-{\delta\:}_{l}\right)\left(1-{\widehat{Pr}}^{\mathcalligra{w}}\left({\dot{\widehat{\varvec{x}}}}_{l,i,t}\right)\right)+{\delta\:}_{l}{\xi\:}_{l-1,t}\:,$$

$$\:\forall\:\:2\le\:l\le\:L;\:\:\:\left[{\varvec{S}}_{l,i}\right]={N}_{l-1}^{N}{N}_{l}^{M}$$

13 $$\:{\xi\:}_{l,t}\left({\varvec{M}}_{l}\left({\varvec{S}}_{l,t}.{\stackrel{\sim}{\dot{\widehat{\varvec{x}}}}}_{l-1,t}\right),{\delta\:}_{l}\right)=\frac{\sum\:_{i=1}^{{N}_{l}^{N}}{\xi\:}_{l,i,t}\left({M}_{l,i}\left({\varvec{S}}_{l,i,t}.{\stackrel{\sim}{\dot{\widehat{\varvec{x}}}}}_{l-1,t}\right),{\delta\:}_{l}\right)}{{N}_{l}^{N}};$$

$$\:\left[{\varvec{S}}_{l}\right]={N}_{l}^{N}{N}_{l-1}^{N}{N}_{l}^{M}$$

The cost value of the first layer is $\:{\xi\:}_{1,t}=\frac{\sum\:_{i=1}^{{N}_{1}^{N}}1-{\widehat{Pr}}^{\mathcalligra{w}}\left({\dot{\widehat{x}}}_{1,i,t}\right)}{{N}_{1}^{N}}$ and $\:{\widehat{\varvec{x}}}_{1,t}={\widehat{\varvec{x}}}_{input,t}\left({\varvec{u}}_{t}\right)$. In (12), $\:{\xi\:}_{l,i,t}$ denotes the internal risk of the neuron $\:i$ in layer $\:l$ at time $\:t$. This risk is influenced by the synaptic connections vector $\:{\varvec{S}}_{l,i,t}$ of the corresponding masks and the final refined values of the neurons of the previous layer $\:{\stackrel{\sim}{\dot{\widehat{\varvec{x}}}}}_{l-1,t}$. The product operator in the input argument of $\:\varvec{M}$ is used to consider only the refined values of the neurons to which the masks are connected when calculating the refined mask value. The refinement of the mask values facilitates the final refinement of the neuron value, obtained by (8) as, $\:{\stackrel{\sim}{\dot{\widehat{x}}}}_{l,i,t}=\text{a}\text{r}\text{g}\text{m}\text{a}\text{x}\left({\widehat{Pr}}^{\mathcalligra{w}}\left({\widehat{x}}_{l,i,t}\:\right),{\widehat{Pr}}^{\mathcalligra{w}}\left({\varvec{M}}_{l,i}\left({\varvec{S}}_{l,i,t}^{\text{*}}.{\stackrel{\sim}{\dot{\widehat{\varvec{x}}}}}_{l-1,t}\right)\right)\right)$.

The discount factor in (12) $\:0<\delta\:<1$ degrades, with a user-defined degradation rate, when moving from a layer to the following one, i.e. $\:{\delta\:}_{l-1}>{\delta\:}_{l}>{\delta\:}_{l+1}$, to allow the agents to select their solutions considering the penalty from the previous layer. This factor in its form in (12) ensures that $\:{\xi\:}_{l,i,t}$ remains bounded, i.e. $\:0<{\xi\:}_{l,i,t}<1$.

In the formulation of (12), the first layer $\:l=1$ is skipped because its masks are fully connected to the input layer, receiving their inputs directly, to ensure that all input features are considered in the subsequent layers. Therefore, the initial value $\:{\xi\:}_{1,t}$ is known and calculated directly based on the input data. Consequently, the optimization wave flow starts at the second layer$\:\:l=2$ and continues through the selected layers until reaching the output layer $\:L$.

The accumulated risk at the layer level $\:{\xi\:}_{l,t}$, as defined in (13), is calculated by taking the mean of the risks of all the refined neurons within the same layer. This value is then used to calculate the internal risks of the neurons in the subsequent layer. By minimizing each of the risks $\:{\left\{{\xi\:}_{l,i,t}\right\}}_{i=1}^{{N}_{l}^{N}}$, the overall accumulated risk of a layer (13) is also minimized.

The vector $\:{\varvec{S}}_{l,i,t}$ is sampled by the search agent of the evolutionary optimizer and combined with all the synaptic connections of the masks within the same layer to form $\:{\varvec{S}}_{l,t}$, i.e. $\:{\varvec{S}}_{l,t}={\left\{{\varvec{S}}_{l,i,t}\right\}}_{i=1}^{{N}_{l}^{N}}$. The optimal synaptic connections $\:{\varvec{S}}_{l,t}^{\text{*}}$ is then determined after finding all $\:{\left\{{\varvec{S}}_{l,i,t}^{\text{*}}\right\}}_{i=1}^{{N}_{l}^{N}}$ that minimize the internal cost function (12) and consequently (13). The dimension of the synaptic structure of the masks of a specific neuron is $\:\left[{S}_{l,i}\right]={N}_{l-1}^{N}{N}_{l}^{M}$. The dimension of the solution vector containing the entire synaptic connection structure for all the masks within the same layer is $\:\left[{\varvec{S}}_{l}\right]={N}_{l}^{N}{N}_{l-1}^{N}{N}_{l}^{M}$.

Determining the optimal synaptic connections for all the layers $\:{\left\{{\varvec{S}}_{l,t}^{\text{*}}\right\}}_{l=2}^{L}$ allows the construction of $\:{\varvec{S}}_{t}^{\text{*}}$, i.e. $\:{\varvec{S}}_{t}^{\text{*}}={\left\{{\varvec{S}}_{l,t}^{\text{*}}\right\}}_{l=2}^{L}$. This represents the overall synaptic connection structure for all the masks within the selected layers of the model. Consequently, all neurons in the model are refined with their final values and therefore the final output of the model is refined by deploying $\:{\varvec{S}}_{t}^{\text{*}}$ in (1) and (2) as

14 $$\:{\stackrel{\sim}{\dot{\widehat{\varvec{x}}}}}_{t}=f\left({\varvec{u}}_{t},\varvec{\theta\:},{\varvec{S}}_{t}^{\text{*}}\right)$$

15 $$\:{\stackrel{\sim}{\dot{\widehat{\varvec{y}}}}}_{t}=g\left({\stackrel{\sim}{\dot{\widehat{\varvec{x}}}}}_{t}\right)$$

Accordingly, the prospect risk loss $\:{\mathcal{L}}^{r}$ in (11) is calculated for the refined final output $\:{\stackrel{\sim}{\dot{\widehat{\varvec{y}}}}}_{t}$. This loss is minimized since it relies on the values and probabilities of the neurons that have been successively optimized with the single wave scheme. Therefore, the synaptic rewiring optimization problem for the selection of $\:{\varvec{S}}_{t}^{\text{*}}$ can be generally formalized as follows,

16.1 $$\:\text{min}{\mathcal{L}}_{t}^{r}\left(f\left({\varvec{u}}_{t},\varvec{\theta\:},{\varvec{S}}_{t}\right)\right)$$

16.2 $$\:s.t.\:\:\:\:{\varvec{S}}_{l,i,t}^{\text{*}}=\underset{{\left\{{\varvec{S}}_{l,i,t}\right\}}_{l=1}^{{N}_{l}^{M}}}{\text{argmin}}\left\{\left(1-{\delta\:}_{l}\right)\left(1-{\widehat{Pr}}^{\mathcalligra{w}}\left({\dot{\widehat{x}}}_{l,i,t}\right)\right)+{\delta\:}_{l}{\xi\:}_{l-1,t}\right\}\:\:\:\forall\:\:2\le\:l\le\:L$$

$$\:\text{s}.\text{t}.\:\:\:0<{\xi\:}_{l,t}<1$$

This bilevel optimization formulation in (16) requires the optimizer to solve the outer level (16.1) by addressing the inner level (16.2), ensuring the condition $\:0<{\xi\:}_{l,t}<1$ is satisfied. This process involves determining the synaptic structure solution $\:{\varvec{S}}_{l,i,t}^{\text{*}}$ at time $\:t$ that minimizes the local risk $\:{\xi\:}_{l,i,t}$ of neuron $\:{x}_{l,i,t}$, while considering the risk of neurons in the previous layer $\:{\xi\:}_{l-1,t}$.

**Fig. 3:**
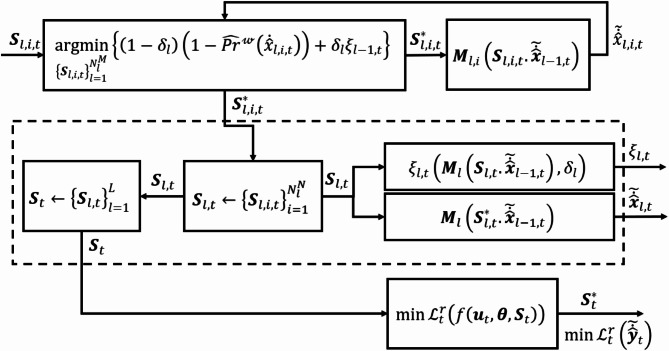
Block diagram illustrating the relationship between the bilevel optimization formulation in (16) and the components of the synaptic plasticity method used to calculate the cost function values.

When the optimal solution is realized in the model, the prospect risk loss of the final refined output $\:{\mathcal{L}}_{t}^{r}\left({\stackrel{\sim}{\dot{\widehat{\varvec{y}}}}}_{t}\right)$ is minimized. Figure 3 illustrates the relationships between the optimization problems and the cost functions. Algorithm 1 summarizes the steps of the proposed single-wave scheme for synaptic rewiring, aimed at finding the optimal synaptic structure. Theoretical walkthrough example and proof of this method are provided in the supplementary material.

**Fig. 4:**
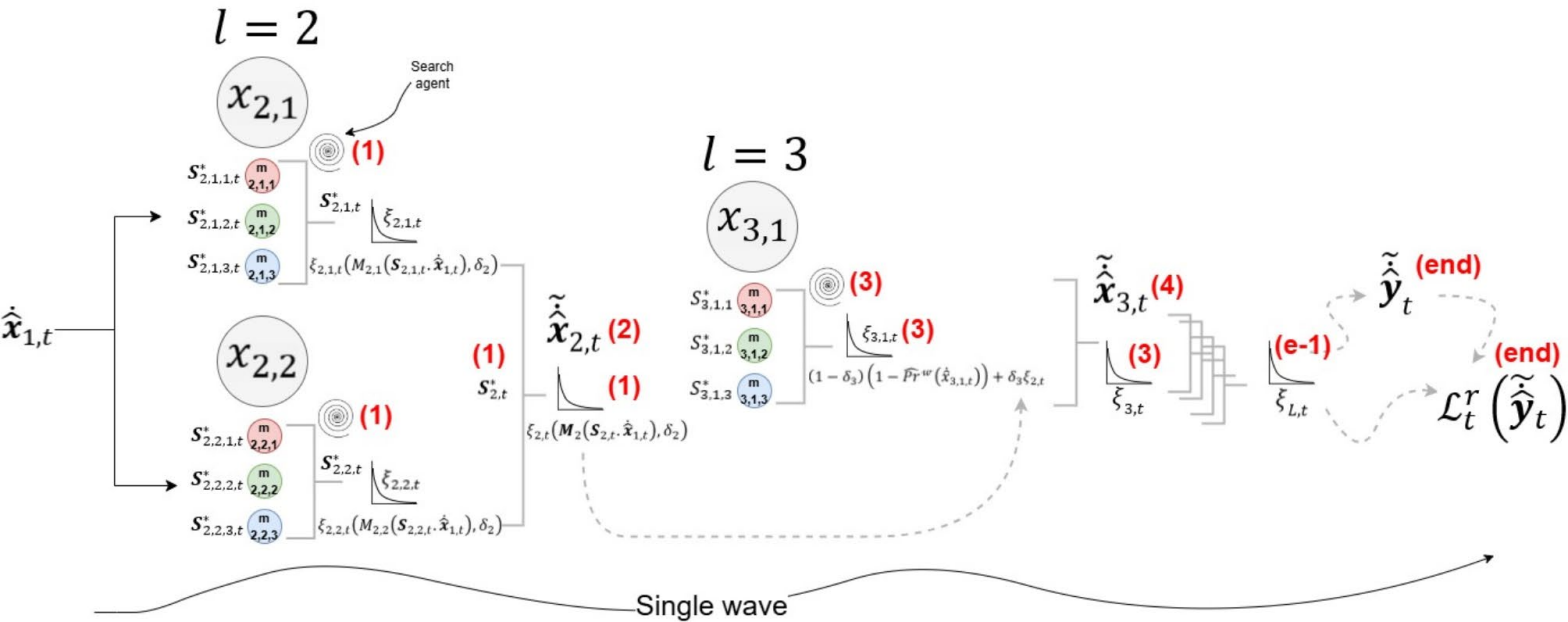
An illustrative diagram showing the flow of the single-wave scheme for the synaptic rewiring process. The steps determine the synaptic structure and the sequence to obtain the final refined neuron values and assess their associated costs.

Figure 4 illustrates the workflow of the single-wave scheme, showing the vector $\:{\dot{\widehat{\varvec{x}}}}_{1,t}$ is initialized from the fully connected layer $\:l=1$ and utilized in layer $\:l=2$. The single wave starts in the second layer where step (1) simultaneously determines $\:{\mathbf{S}}_{2,\text{t}}^{\text{*}}$ by finding $\:{\varvec{S}}_{\text{2,1},t}^{*}\:$and $\:{\varvec{S}}_{\text{2,2},t}^{*}$ that minimize $\:{\xi\:}_{\text{2,1},t}$ and $\:{\xi\:}_{\text{2,2},t}$, respectively. Consequently, when combined into $\:{\varvec{S}}_{2,t}$, $\:{\xi\:}_{2,t}$ is minimized.

Finding $\:{\varvec{S}}_{2,t}^{*}$ allows the determination of the final refined values of the vector $\:{\stackrel{\sim}{\dot{\widehat{\varvec{x}}}}}_{2,t}$ in step (2). Step (3) then begins, which is similar to step (2), to determine $\:{\varvec{S}}_{3,t}^{*}$, enabling the refinement of the neuron vector $\:{\stackrel{\sim}{\dot{\widehat{\varvec{x}}}}}_{3,t}$ in step (4). These steps continue through subsequent layers until obtaining $\:{\varvec{S}}_{L,t}^{*}$, which minimizes the accumulated risk of the output layer $\:{\xi\:}_{L,t}$ in step (end-1). This allows the formulation of the complete synaptic connection structure $\:{\varvec{S}}_{t}^{*}=\left[{\varvec{S}}_{2,t}^{*},{\varvec{S}}_{3,t}^{*},\dots\:,{\varvec{S}}_{L,t}^{*}\right]$, consequently determining the final refined value of the model’s output $\:{\stackrel{\sim}{\dot{\widehat{\varvec{y}}}}}_{t}$. The risk $\:{\mathcal{L}}_{t}^{r}\left({\stackrel{\sim}{\dot{\widehat{\varvec{y}}}}}_{t}\right)$ is then measured in the last step (end), ensuring it is minimized.

**Algorithm 1:** Single wave for synaptic rewiring.

*1*	*# Initialization*
*2*	define ${\updelta }_{2}$ and its degradation rate;
*3*	calculate ${\dot{\widehat{{\varvec{x}}}}}_{1,t}$;
*4*	calculate ${\xi }_{1,t}$;
*5*	define ${\left\{{N}_{l}^{M}\right\}}_{l=1}^{L}$; *# select the layers to apply synaptic plasticity and *
*6*	*# set *${N}^{M}$*for each selected layer.*
*7*	**while** ${\mathcal{L}}_{t}^{r}\left({\dot{\widehat{{\varvec{y}}}}}_{t}\right)$ is not minimized {
*8*	*#* *to find *${\left\{{{\varvec{S}}}_{l,t}^{*}\right\}}_{l=1}^{L}$
*9*	** for **each $2\le l\le L${ *# moving forward starting from *$l=2.$
10	define the number of search agents = ${N}_{l}^{N}$;
11	** while** ${\xi }_{l,t}$in (13) is not minimized {
12	** parfor** each ${x}_{l,i}${ *# parallel process for all the neurons in the layer.*
13	define ${{\varvec{S}}}_{l,i,t}$that minimizes ${\xi }_{l,i,t}$ in (12) s.t. ${\xi }_{l,t}\left({{\varvec{S}}}_{l,t}\right)$in (13) is minimized;
14	${{\varvec{S}}}_{l,t}\leftarrow {\left\{{{\varvec{S}}}_{l,i,t}\right\}}_{i=1}^{{N}_{l}^{M}}$;
15	} *# end parfor*
16	output ${{\varvec{S}}}_{l,t}^{*}$;
17	} *# end while*
18	${{\varvec{S}}}_{t}^{*}\leftarrow {\left\{{{\varvec{S}}}_{l,t}^{\boldsymbol{*}}\right\}}_{l=2}^{L}$; s.t. ${\mathcal{L}}_{t}^{r}\left({\widetilde{\dot{\widehat{{\varvec{y}}}}}}_{t}\right)$is minimized;
19	} *# end for*
20	} *# end while*

##### Information source

An iterative evolutionary algorithm to determine the optimal synaptic connection structure for each generated mask during the deployment phase is highly time-demanding. This challenge necessitates further studies in future research. However, in this work, aiming to reduce the requirement for online optimization, we employ the information source $\:\pi\:$ proposed in^8^. This information source, derived from auxiliary sensors or external systems, delivers information about the changes in the input distribution during the deployment phase.

By integrating the information module into the ANN model under consideration, it becomes aware of the changes in its deployment environment and can adapt accordingly to generate more accurate results^8^. In this paper, we utilize the information source $\:{\pi\:}_{t}$ to quantify distribution changes in the input data, without affecting the calculation process of the neuros or their outputs.

Given that $\:{\mathcal{P}}^{t}$ may not change for each newly coming input $\:{\varvec{u}}_{t}^{t}$, there may be negligible or no difference between $\:{\pi\:}_{t}$ and $\:{\pi\:}_{t-1}$. Hence, the synaptic rewiring optimization process may not be required at each time. Consequently, synaptic connections $\:\varvec{S}$ are rewired only when a certain threshold of the difference between $\:{\pi\:}_{t}$ and $\:{\pi\:}_{t-1}$ is exceeded. To address this, we use a user-defined hyperparameter $\:\alpha\:$, such that,

17 $$\:tanh\left(\left|{\pi\:}_{t}-{\pi\:}_{t-1}\right|\right)\ge\:\alpha\:\:;\:\:\:\:0<\alpha\:<1$$

The use of the hyperbolic tangent function ensures that the absolute difference between the current value of the information source and the previous one remains within the range of 0 and 1.

#### Storage/retrieval memory

**Fig. 5:**
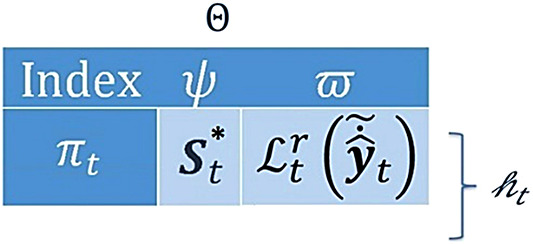
The general design of the proposed memory module.

To store and retrieve $\:{\varvec{S}}^{\varvec{*}}$ for all selected neurons along with the corresponding $\:\pi\:$ and $\:{\mathcal{L}}^{r}$ for later use, we propose a database-like memory module with the ability to forget inaccurate records using the update functionality. Each row in the memory $\:{\Theta\:}$, indexed by $\:\pi\:$, contains two fields: $\:\psi\:$ for the $\:{\varvec{S}}^{\varvec{*}}$ vectors and $\:\varpi\:$ for their corresponding risks $\:{\mathcal{L}}_{t}^{r}\left({\stackrel{\sim}{\dot{\widehat{\varvec{y}}}}}_{t}\right)$. This memory has three main functionalities: storing new entries, retrieving old records, and updating current records. The size of this memory $\:{\mathcalligra{h}}_{t}$ is variable during the deployment phase. Figure 5 shows the general structure of $\:{\Theta\:}$.

It is important to note that the inequality (17) may be satisfied by a range of $\:\pi\:$ values. This observation determines whether a new entry should be stored or an existing record can be retrieved. The range for $\:{\pi\:}_{t}$ that satisfies (17) is defined as,

18 $$\:\varvec{r}\left({\pi\:}_{t}\right)=\left[{\pi\:}_{t}-\alpha\:,\:{\pi\:}_{t}+\alpha\:\right]$$

The difference margin vector $\:\varvec{r}\left({\pi\:}_{t}\right)$ contains all possible values of $\:{\pi\:}_{t}$ allowed by $\:\alpha\:$, permitting the model to reuse the stored $\:{\varvec{S}}^{\varvec{*}}$. During the deployment of the model, when a new sample $\:{\varvec{u}}_{t}^{t}\sim{\mathcal{P}}^{t}$ with new information $\:{\pi\:}_{t}$ satisfying (17) is encountered, there are two possibilities:

##### Storage

If no available information in the memory falls within the range of the new information, i.e. $\:\forall\:\pi\:\in\:\varTheta\:,\pi\:\notin\:\varvec{r}\left({\pi\:}_{t}\right)$, a new synaptic rewiring $\:{\varvec{S}}_{t}^{\text{*}}$ will be generated using (16), deployed in the model, and then stored in the memory along with its resulting $\:{\mathcal{L}}_{t}^{r}\left({\stackrel{\sim}{\dot{\widehat{\varvec{y}}}}}_{t}\right)$ indexed by the $\:{\pi\:}_{t}$, as follows,

19 $$\:\psi\:\left({\pi\:}_{t}\right)\leftarrow\:{\varvec{S}}_{t}^{\text{*}}$$

20 $$\:\varpi\:\left({\pi\:}_{t}\right)\leftarrow\:{\mathcal{L}}_{t}^{r}\left({\stackrel{\sim}{\dot{\widehat{\varvec{y}}}}}_{t}\right)$$

##### Retrieval

If at least one record in the memory has $\:\pi\:\in\:\varvec{r}\left({\pi\:}_{t}\right)$, the $\:{\varvec{S}}^{\text{*}}$ entry corresponding to the lowest $\:{\mathcal{L}}^{r}\left(\stackrel{\sim}{\dot{\widehat{\varvec{y}}}}\right)$ is selected and deployed in the model to process $\:{\varvec{u}}_{t}^{t}$.

##### Update

After processing $\:{\varvec{u}}_{t}^{t}$ with the retrieved synaptic connections $\:{\varvec{S}}^{\text{*}}$, a new prospect risk loss $\:{\mathcal{L}}_{t}^{r}\left({\stackrel{\sim}{\dot{\widehat{\varvec{y}}}}}_{t}\right)$ is measured. If this loss is worse than the one in the memory corresponding to the retrieved $\:{\varvec{S}}^{\text{*}}$, i.e.

21 $$\:{\mathcal{L}}_{t}^{r}\left({\stackrel{\sim}{\dot{\widehat{\varvec{y}}}}}_{t}\right)-\varpi\:\left(\pi\:\right)>\beta\:\:\:\:;\:\:\:\:\:0<\beta\:<1$$

it indicates that the existing synaptic connections are outdated. Therefore, this entry should be updated. The parameter $\:\beta\:$ is a user-defined threshold for the prospect risk loss difference, representing the user’s tolerance for the increased risk.

Updating the memory entries is crucial to continuously improving the model’s accuracy and avoiding the repetition of non-optimal connections selected by the optimizer. Updating a record follows three steps: (i) a new $\:{\varvec{S}}_{new,t}^{\text{*}}$ for $\:{\pi\:}_{t}$ is determined using (16), (ii) the model processes $\:{\varvec{u}}_{t}^{t}\:$with the new $\:{\varvec{S}}_{new,t}^{\text{*}}$, and the corresponding $\:{\mathcal{L}}_{new,t}^{r}\left({\stackrel{\sim}{\dot{\widehat{\varvec{y}}}}}_{new,t}\right)$ is measured, and (iii) all $\:\pi\:\in\:\varvec{r}\left({\pi\:}_{t}\right)$ in the memory are updated with the new combination of $\:{\varvec{S}}_{new,t}^{\text{*}}$ and $\:{\mathcal{L}}_{new,t}^{r}\left({\stackrel{\sim}{\dot{\widehat{\varvec{y}}}}}_{new,t}\right)$.

The integration of the information source and memory module decreases the need for frequent activation of the synaptic rewiring process. This reduction is effective not only for subsequent inputs with distribution information that falls within the same range, $\:\varvec{r}\left({\pi\:}_{t}\right)$, but also for new situations with input distribution information similar to previously encountered cases. By retrieving the stored information, the system can efficiently adapt to new inputs without repeatedly undergoing the computationally intensive synaptic rewiring process. Algorithm 2 summarizes the proposed memory scheme.

**Algorithm 2:** Storage/ retrieval memory module.

*21*	*# in the training/deployment phases*
22	**for** each ${{\varvec{u}}}_{t}^{t}${
23	** if** $\forall \pi \in\Theta ,\pi \notin {\varvec{r}}\left({\pi }_{t}\right)$ {
24	*# store*
25	generate ${{\varvec{S}}}_{t}^{*}$ using (16);
26	deploy:${\widetilde{\dot{\widehat{{\varvec{y}}}}}}_{t}=g\left(f\left({{\varvec{u}}}_{{\varvec{t}}},{\varvec{\theta}},{{\varvec{S}}}_{t}^{*}, {\pi }_{t}\right)\right)$;
27	$\psi \left({\pi }_{t}\right)\leftarrow {{\varvec{S}}}_{t}^{*}$;
28	$\varpi \left({\pi }_{t}\right)\leftarrow {\mathcal{L}}_{t}^{r}\left({\widetilde{\dot{\widehat{{\varvec{y}}}}}}_{t}\right)$ ;
29	}
30	** else if** $\exists \pi \in\Theta ,\pi \in {\varvec{r}}\left({\pi }_{t}\right)$ {
31	*# retrieve*
32	${{\varvec{S}}}_{t}^{*}\leftarrow \psi \left(\pi \in {\varvec{r}}\left({\pi }_{t}\right)\right) s.t. \varpi (\pi )=min\left(\varpi \left(\forall \pi \in {\varvec{r}}\left({\pi }_{t}\right)\right)\right)$;
33	deploy: ${\widetilde{\dot{\widehat{{\varvec{y}}}}}}_{t}=g\left(f\left({{\varvec{u}}}_{{\varvec{t}}},{\varvec{\theta}},{{\varvec{S}}}_{t}^{*}, {\pi }_{t}\right)\right)$;
34	** if** ${\mathcal{L}}_{t}^{r}\left({\widetilde{\dot{\widehat{{\varvec{y}}}}}}_{t}\right)-\varpi ({\pi }_{t})>\beta$ {
*35*	*# update*
36	generate ${{\varvec{S}}}_{t,new}^{*}$ using (16);
37	deploy: ${\dot{\widehat{{\varvec{y}}}}}_{t}=g\left(f\left({{\varvec{u}}}_{{\varvec{t}}},{\varvec{\theta}},{{\varvec{S}}}_{t,new}^{*}, {\pi }_{t}\right)\right)$;
38	$\psi \left({\varvec{r}}\left({\pi }_{t}\right)\right)\leftarrow {{\varvec{S}}}_{new,t}^{*}$;
39	$\varpi ({\varvec{r}}\left({\pi }_{t}\right))\leftarrow {\mathcal{L}}_{new,t}^{r}({\widetilde{\dot{\widehat{{\varvec{y}}}}}}_{new,t})$;
40	}
41	}
42	}

## Implementation and experimental results

The following experiments aim to validate the proposed method for addressing distributional changes using both synthetic (toy) and real datasets, with a focus on evaluating the model’s accuracy. In all experiments, we use the following parameters in the synaptic plasticity model, which are arbitrarily selected:

- $\:{\left\{{\left\{{N}_{l,i}^{M}\right\}}_{i=1}^{{N}_{l}^{N}}\right\}}_{l=1}^{L}=3$: i.e. three masks are generated for each neuron in each layer (except the input layer) of the model.
- $\:\alpha\:=0.5$: the difference threshold for the accepted range between $\:{\pi\:}_{t}$ and $\:{\pi\:}_{t-1}$.
- $\:\beta\:=0.5$: the threshold for the prospect risk loss difference.
- Synaptic rewiring is performed using the single-wave scheme with the dwarf mongoose^41^ evolutionary algorithm.
- The initial value of the discount factor is $\:{\delta\:}_{2}=0.5$, with a constant degradation rate of $\:\frac{{\delta\:}_{2}}{L-1}$ as the single wave moves forward in the subsequent layers.

For comparison, we address the same task using several well-known model-agnostic methods that provide regularization mechanisms for handling distributional changes: TENT^15^ (test-time technique with uncertainty estimation), MAML^12^ (meta-learning method), and TTT Online^17^ (test-time method with model generalization). In our recent work, the information module^8^ and the prospect certainty method^36^ were proposed. Here, we carried out experiments based on both methods to evaluate the effect of the synaptic plasticity method in this paper. To assess the method’s capability to mitigate distributional changes and adapt to the deployment environment, we also compare the model’s accuracy when processing the testing dataset (which represents the distributional change) with that obtained when the model processes a familiar training dataset.

### Toy experiments

#### Experiment 1. classification

In this experiment, six classes from the Cifar-10 dataset^42^ (i.e., Bird, Dog, Automobile, Ship, Horse, and Airplane) are used for training and testing. The data are independently and identically distributed (IID). For evaluating distributional changes, the ImageNet^43^ and COCO^44^ datasets are utilized for testing, representing out-of-distribution (OOD) data in the classification tasks. To ensure a comparative evaluation, the categories from the testing datasets are combined and assigned the same index as in the training dataset.

The baseline model selected for this experiment is ResNet-9, whose architecture is detailed in^45^. All methods used are configured with the following settings:

- Number of epochs: 120.
- Batch size: 64.
- Trainer: Adam with default settings of the TensorFlow 2.11.0.
- Learning rate: constant with halving at the stagnation of validation loss for more than three epochs.

Other hyperparameters, unless specified, were optimized using the Optuna package^46^ with the tree-structured Parzen estimator search strategy^47^ and the threshold pruner^46^.

We compare our approach with the selected methods using the mean cross-entropy (MCE) loss metric. Each method was executed five times. The mean error of each approach is presented in Table 1.

**Table 1:** Experimental results of the toy classification model.

Method	Mean cross-entropy error $\:\downarrow\:$			Similarity $\:\downarrow\:$
	CIFAR−10 $\:\:\left(IID\right)$	ImageNet $\:\:\left(OOD1\right)$	COCO $\:\:\left(OOD2\right)$	$\:mean\:\left(OOD1,OOD2\right)-IID$
Baseline	0.28	12.89	19.31	15.82
Baseline + TENT	0.13	08.49	10.03	09.13
Baseline + MAML	0.08	06.01	11.39	08.62
Baseline + TTT online	0.13	08.32	09.15	08.61
Baseline + information module	0.12	01.19	01.81	01.38
Baseline + prospect certainty layer	0.10	01.11	01.90	01.41
Baseline + neuroplasticity (ours)	**0.01**	**00.48**	**00.51**	**00.49**

Bold values represent the best results among the compared methods.

The baseline results are instrumental in understanding how the compared approaches perform. As shown in Table 1, the MCE error by our method is quite consistent between the training and testing datasets, relative to the compared methods. For instance, using our method, the MCE difference between the IID and OOD datasets is only 0.49, followed by 1.38 when incorporating the information module within the model. This small difference between the training and the testing dataset demonstrates that our method effectively mitigates the impact of distributional changes.

#### Experiment 2. regression

Two test cases are designed in line with^48^. The following function is sampled to generate a synthetic dataset:

22 $$\:f\left(u\right)=u\:{sin}u+{\epsilon}_{1}u+{\epsilon}_{2}$$

where $\:{\epsilon}_{1}$, $\:{\epsilon}_{2}\sim\:\mathcal{N}(0,\:0.3)$ are disturbances, representing distribution changes.

##### Test case 1

1000 samples are generated from $\:u\in\:[0,\:10]$ for training the model, and 200 test samples from $\:u\in\:[10,\:15]$ are used for testing.

##### Test case 2

Similar to test case 1, but with adding a white Gaussian noise $\:\sim\mathcal{N}(-\text{1,1})$ to the region $\:u\:\in\:\:[5,\:8]$ to simulate distributional changes. The entire sequence in the range $\:u\in\:[0,\:15]$ is repeated twice to test the effect of the memory module when the model encounters a situation similar to a previous experience.

The settings used for the compared methods are as follows:

- Baseline: Dense(32, ReLU) - Dense(32, ReLU).
- Number of epochs: 700.
- Batch size: 32.
- Trainer: Adam with the default parameters of the TensorFlow 2.11.0.

The data is entered into the model sequentially. Each test case is conducted using each method five times to calculate the average root mean square error (RMSE) and the average inference speed. To evaluate the method’s behavior following the change in data distribution while keeping the model running, RMSE is calculated for each data region separately. The inference speed is measured for test case 2 after the end of each pass while keeping the model running to test the effect of memorizing experiences by the memory module.

The results of both test cases, which highlight the strengths and limitations of the compared methods, are shown in Table 2, in which the bricks indicate the range of the input data $\:u$.

**Table 2:** Experimental results of the toy regression model.

Method	Root mean square error $\:\downarrow\:$								Average inference speed $\:\downarrow\:$	
Baseline +	Test1 (IID) $\:[0,\:10]$	Test1 (Pred) $\:\:[10,\:15]$	First pass of Test2 (IID) $\:\:[0,\:6],\left[\text{8,10}\right]$	First pass of Test2 (OOD) $\:\:[6,\:8]$	First pass of Test2 (Pred) $\:\:[10,\:15]$	Second pass of Test2 (IID) $\:\:[0,\:6],\left[\text{8,10}\right]$	Second pass of Test2 (OOD) $\:\:[6,\:8]$	Second pass of Test2 (Pred) $\:\:[10,\:15]$	First pass ($\:ms$)	Second pass ($\:ms$)
TENT	0.012	3.001	0.013	3.877	5.138	0.014	3.877	5.141	1.035	1.030
MAML	0.024	3.425	0.020	3.910	5.317	0.023	3.911	5.315	0.491	0.494
TTT online	**0.003**	2.518	**0.009**	2.867	3.011	0.008	2.866	3.011	1.480	1.481
Information module	0.061	0.502	0.058	0.739	0.512	0.058	0.738	0.510	**0.304**	**0.302**
Prospect certainty layer	0.048	0.209	0.065	0.715	0.954	0.064	0.716	0.952	0.412	0.413
Neuroplasticity (ours)	0.004	**0.014**	0.011	**0.014**	**0.024**	**0.007**	**0.013**	**0.024**	3.243	0.794

Bold values represent the best results among the compared methods.

As shown in Table 2, the RMSE value for the IID samples resulting from our neuroplasticity method demonstrates competitive performance similar to the other methods, suggesting robustness in consistent data distributions. In contrast, from OOD samples, our method achieves much lower RMSE values compared with the others, indicating improved adaptability and response to data that deviates from the training distribution. This superior performance in OOD scenarios can be attributed to the adaptive synaptic structure. In addition, it can be seen from Table 2, to conduct prediction in $\:u\in\:[10,\:15]$, our method shows commendable accuracy with lower RMSE values among the other methods, reinforcing its ability to adapt well to data outside the training dataset.

We assess the functionality of the memory module by presenting inference speed results. From the inference speed shown in Table 2, our method is not the fastest in the first pass. This arises when the model encounters a new input distribution, i.e. our method requires time for the synaptic connections to rewire and adapt to the new situation. This rewiring process leads to increased processing time. Nevertheless, our method performs reasonably well compared to the test-time methods, as shown in Table 2.

In the second pass, our method notably exhibits significant improvement, indicating efficient utilization of the previously learned knowledge, as shown in Table 2. This improvement can be attributed to the memory module, which is unique to our method and reduces the need for online optimization. As a result, when encountering a familiar distribution stored in the memory, our method processes it in a time comparable to other methods. This highlights the advantage of the memory module in reducing processing times in familiar situations.

Despite these strengths, our method still shows limitations in the processing time, which is due to the application of the neuronal masks across all layers of the model, leading to a high dimensional MINLP problem. This highlights the need for further studies in future work, given the importance of online adaptation to changing data distributions in real-world applications.

### Real dataset experiments

In this experiment, the task of identifying pedestrians and predicting their moving trajectories is undertaken to simulate diversity in a real-world setting that exhibits characteristics of distributional changes. The human-centered nature of this task makes the model’s decision highly sensitive, especially in critical applications such as autonomous driving, where a single error could be catastrophic.

This experiment encompasses three primary subtasks: (i) identification and categorization of pedestrians, (ii) tracking them, and (iii) predicting their future locations^36^. For implementation, we employ CNN-M-2048 and LSTM sub-models described in^49,50^, respectively, as the base model. Detailed architecture and configuration settings are provided in^36^.

The model is trained using the PIE dataset^51^ and tested using the JAAD dataset^52^, the PANDA dataset^53^, and the Waymo Perception dataset^54^.

#### Classification results

The results of this binary classification task (i.e., person or not person), measured in terms of mean average precision ($\:mAP$), are presented in Table 3.

**Table 3:** Classification results comparison using the real dataset.

Method	$\:mAP$ $\:\uparrow\:$				Similarity $\:\downarrow\:$
	PIE $\:\left(IID\right)$	JAAD $\:\left(OOD1\right)$	PANDA $\:\left(OOD2\right)$	Waymo perception $\:\left(OOD3\right)$	$\:IID-mean\left(OOD1,OOD2,\:OOD3\right)$
Baseline +	84.16	46.26	33.01	34.78	46.14
TENT	84.10	44.01	37.64	42.18	42.82
MAML	84.21	58.62	39.17	47.59	35.75
TTT online	84.16	54.43	41.56	40.13	38.77
Information module	81.87	79.18	77.12	78.98	**03.44**
Prospect certainty	87.05	82.12	79.22	81.47	06.11
Neuroplasticity (ours)	**89.78**	**85.51**	**84.98**	**84.00**	04.95

Bold values represent the best results among the compared methods.

Two key observations can be drawn from Table 3. First, the superiority of the accuracy of our method is evident in scenarios both when the data distribution is consistent with the training data (IID) and also when the data exhibits changes in its distribution (OOD). This indicates that our method is highly adaptable, due to the synaptic rewiring triggered by the information source, and maintains high accuracy when the data distribution deviates from the training distribution.

In addition, as shown in Table 3, the difference in $\:mAP$ between the IID and OOD datasets is small, further highlighting the robustness of our method. This small difference demonstrates the ability to adapt to new environments without requiring retraining, thus effectively mitigating the impacts of distributional changes.

The results of our synaptic plasticity method fall within the acceptable range shown by the prospect certainty and the information module, which share similar features of low determinism during the deployment phase. Furthermore, our method surpasses other methods that maintain the model’s deterministic nature during its deployment, highlighting the effectiveness of our method in adapting to changing data distributions.

#### Regression results

The aim of the regression task is to identify the movement of pedestrians represented by their bounding boxes in the images. The results are compared using the average intersection over union ($\:AIoU$) metric which evaluates the similarity between the estimated bounding box after 10 frames and the labels of the same frame.

**Table 4:** Regression results comparison using the real dataset.

Method	$\:\varvec{A}\varvec{I}\varvec{o}\varvec{U}\:\uparrow\:$				Similarity $\:\downarrow\:$
	PIE	JAAD	PANDA	Waymo perception	$\:IID-mean\left(OOD1,OOD2,\:OOD3\right)$
Baseline +	*41.11*	*19.14*	*14.26*	*17.43*	*24.17*
TENT	49.83	31.08	26.47	27.88	21.35
MAML	47.02	28.19	27.68	26.90	19.43
TTT online	49.79	31.97	27.54	28.22	20.55
Information module	40.54	39.71	37.98	39.01	**01.64**
Prospect certainty	49.25	41.82	39.38	41.09	08.49
Neuroplasticity (ours)	**56.72**	**45.00**	**43.93**	**46.21**	11.67

Bold values represent the best results among the compared methods.

Results of the compared methods are presented in Table 4, showing that our method achieves the best accuracy. It demonstrates that the neuroplasticity method is task-independent, thus having a high reliability. In addition, our method gets a similarity value of 11.67, which, although higher than the values by the Information Module (1.64) and Prospect Certainty (8.49), still indicates competitive performance compared with the other methods. This suggests that our method not only adapts relatively well to different data distributions but also maintains high predictive accuracy.

It is worth noting that the overall performance in the classification task (see Table 3) is better than in the regression task (see Table 4). This discrepancy is due to the difference between the types of outputs (i.e., categorical vs. numerical), which affects the model’s behavior in response to the implicit uncertainty. This observation also applies to the benchmark methods. Moreover, the similarity degree of our method demonstrates between the IID and OOD samples is better in the synthetic dataset compared with the real-world datasets. This could be attributed to the baseline model used, which is not a focal point of this study.

In summary, the overall accuracy of the models in the conducted experiments verifies that our method is task-independent, improves the adaptability and the accuracy of the ANN model when deployed in a variable environment, and increases the reliability and robustness of the model when used in sensitive applications.

## Conclusions

This study addresses the problem of regularization for ANN models in variable environments with input data distribution changes. Inspired by neuroplasticity, a novel regularization approach is developed in this paper. This method enhances the reliability of model outputs by extending the concept of neuronal masking to neurons in all layers within the model, utilizing the weighted probability mean to refine these outputs.

To enhance the adaptability and minimize the risk of the model output, a synaptic connection module is introduced. This module determines the connections of the generated masks to neurons in the preceding layer. These connections are optimized through the synaptic rewiring process which is triggered by the information of the input distribution provided by the information source. This rewiring process is formulated as a bilevel MINLP problem, aiming to minimize the outer loss of prospect risk of the model output by determining the optimal synaptic connection that minimizes the inner weighted risk of the neurons.

To solve this problem, we propose a single-wave scheme that decomposes the problem into smaller, parallel sub-problems by minimizing the inner cost function in a manner that the aggregated solution also minimizes the outer cost function. To store the determined optimal connections and their corresponding prospect risk loss for the given sample information, a storage/recovery memory module is suggested. This module enables the model to retrieve and leverage previously learned connections when encountering similar situations, thus enhancing the computation efficiency.

The proposed method is validated using both synthetic (toy) and real datasets for classification and regression tasks. Experimental results show significant improvement in prediction accuracy up to 8% despite distributional changes, indicating superior adaptability and robustness. Therefore, the neuroplasticity-based method enhances the model’s accuracy, making it a robust solution when deploying ANN models in real-world applications.

Concerning future work, the effect of each component in this method on the model performance will be further investigated. In addition, the trade-off between accuracy, indeterminacy, processing time, and layer selection needs to be studied.

## Electronic supplementary material

Below is the link to the electronic supplementary material.

Supplementary Material 1

Supplementary Material 2

## Data Availability

Below is a list of datasets that have been utilized to validate the suggested method. To access some of the datasets, registration is required. The CIFAR-10 dataset is available at https://www.cs.toronto.edu/~kriz/cifar.html. The ImageNet dataset is available at: https://www.image-net.org/download.php. The COCO dataset is available at: https://cocodataset.org/#download. The PIE dataset is available at: https://data.nvision2.eecs.yorku.ca/PIE_dataset/. The JAAD dataset is available at: https://data.nvision2.eecs.yorku.ca/JAAD_dataset/. The PANDA dataset is available at: https://gigavision.cn/track/track/?nav=Tracking. The Waymo dataset is available at: https://waymo.com/open/.
