# Supplementary material for: Synaptic plasticity-based regularizer for artificial neural networks
Source: Sci Rep. 2025 Apr 24;15:14330. doi: 10.1038/s41598-025-91635-3 (PMC12022306; doi:10.1038/s41598-025-91635-3)
Supplement: Supplementary file 1 — Supplementary Material 1 [file 41598_2025_91635_MOESM1_ESM.docx]

Type of the Paper (Research)

Title *Synaptic plasticity-based regularizer for artificial neural networks*

Qais Yousef ^1,^* & Pu Li ^2,^*

1 ORCID: [0000-0003-0239-9150].

2 ORCID: [0000-0001-6481-9961].

1,2 Group of Process Optimization, Institute for Automation and Systems Engineering, Technische Universität Ilmenau, P.O. Box 100565, 98684 Ilmenau, Germany

* Correspondence: {qais.yousef, pu.li}@tu-ilmenau.de

**Synthetic Dataset**

| **Input** | **Target Test1** | **Input** | **Target Test2** |
| --- | --- | --- | --- |
| 0.0179 | -0.0031 | 0.0148 | 0.0025 |
| 0.0202 | -0.0099 | 0.0398 | 0.002 |
| 0.0227 | -0.0024 | 0.0427 | 0.0261 |
| 0.0359 | -0.0039 | 0.0429 | -0.0227 |
| 0.0392 | -0.0145 | 0.0547 | 0.009 |
| 0.0421 | 0.0112 | 0.0684 | -0.0464 |
| 0.0518 | 0.0101 | 0.1266 | -0.0119 |
| 0.0566 | 0.0216 | 0.1298 | -0.0052 |
| 0.0592 | 0.0114 | 0.136 | 0.079 |
| 0.061 | 0.0195 | 0.1408 | -0.0474 |
| 0.0747 | -0.0171 | 0.1464 | -0.0635 |
| 0.0904 | 0.0393 | 0.152 | 0.0274 |
| 0.0932 | -0.1066 | 0.1538 | -0.0433 |
| 0.1027 | 0.1044 | 0.1657 | 0.0365 |
| 0.1057 | 0.0501 | 0.1684 | 0.1662 |
| 0.1089 | -0.0013 | 0.1723 | 0.2347 |
| 0.1178 | 0.0177 | 0.1842 | 0.0117 |
| 0.1228 | 0.0203 | 0.1923 | -0.1578 |
| 0.1255 | 0.0618 | 0.1952 | 0.0575 |
| 0.1311 | 0.0325 | 0.1965 | -0.0389 |
| 0.136 | 0.089 | 0.2025 | -0.0248 |
| 0.1484 | 0.0334 | 0.2107 | -0.0825 |
| 0.1669 | 0.0242 | 0.2521 | -0.1013 |
| 0.1676 | 0.1322 | 0.2979 | 0.0905 |
| 0.1739 | -0.0257 | 0.3405 | 0.2042 |
| 0.203 | 0.1267 | 0.3473 | 0.1036 |
| 0.2044 | -0.0497 | 0.3614 | -0.1366 |
| 0.2241 | 0.0019 | 0.3656 | 0.1492 |
| 0.2261 | -0.0823 | 0.3757 | 0.3999 |
| 0.2269 | 0.0135 | 0.3859 | 0.4941 |
| 0.2278 | -0.0256 | 0.4043 | -0.0348 |
| 0.2282 | 0.285 | 0.4045 | 0.3179 |
| 0.2345 | 0.0626 | 0.4049 | -0.0553 |
| 0.247 | 0.0659 | 0.4241 | 0.0336 |
| 0.2474 | 0.0666 | 0.4277 | 0.1423 |
| 0.2477 | 0.1772 | 0.4297 | 0.1701 |
| 0.2482 | 0.0838 | 0.4462 | 0.2825 |
| 0.2562 | 0.0818 | 0.4587 | 0.1156 |
| 0.2599 | 0.0051 | 0.5007 | 0.5904 |
| 0.26 | 0.0383 | 0.5107 | 0.1541 |
| 0.2747 | 0.0478 | 0.5212 | 0.1985 |
| 0.3155 | 0.1398 | 0.5222 | 0.2314 |
| 0.3158 | 0.2591 | 0.5311 | 0.0563 |
| 0.33 | 0.1142 | 0.5393 | 0.5095 |
| 0.3399 | 0.0953 | 0.5396 | 0.2415 |
| 0.3474 | 0.0656 | 0.5466 | 0.2359 |
| 0.3539 | 0.1287 | 0.5653 | -0.1003 |
| 0.37 | 0.0072 | 0.5951 | 0.0793 |
| 0.374 | 0.249 | 0.6043 | -0.1588 |
| 0.3781 | 0.2858 | 0.6079 | 0.3195 |
| 0.3839 | 0.1104 | 0.6191 | 0.6165 |
| 0.3987 | 0.1317 | 0.6212 | 0.6839 |
| 0.4124 | 0.2538 | 0.6259 | 0.2516 |
| 0.4127 | 0.1827 | 0.626 | 0.5489 |
| 0.4167 | 0.3572 | 0.6342 | 1.1846 |
| 0.4225 | 0.2233 | 0.6352 | -0.131 |
| 0.4276 | 0.3054 | 0.6353 | 0.6684 |
| 0.434 | 0.1278 | 0.6368 | 0.4047 |
| 0.4411 | -0.0882 | 0.6421 | 0.1746 |
| 0.4437 | 0.0168 | 0.6422 | 0.6414 |
| 0.4458 | 0.7523 | 0.6492 | 0.2454 |
| 0.4612 | 0.3779 | 0.6581 | 0.9479 |
| 0.477 | 0.3088 | 0.6881 | -0.0974 |
| 0.4795 | 0.0434 | 0.6952 | 0.6761 |
| 0.4799 | 0.1978 | 0.7058 | 0.1758 |
| 0.4983 | 0.3416 | 0.7071 | 0.4267 |
| 0.499 | 0.217 | 0.734 | 0.8836 |
| 0.5166 | 0.1142 | 0.7379 | 0.6091 |
| 0.5363 | 0.1266 | 0.7439 | 0.5204 |
| 0.5364 | 0.2259 | 0.7442 | 0.5754 |
| 0.5456 | 0.2825 | 0.7458 | 1.4404 |
| 0.5936 | 0.6693 | 0.7738 | 1.0058 |
| 0.6012 | 0.0654 | 0.7759 | 0.1979 |
| 0.6096 | 0.6866 | 0.7834 | 0.5255 |
| 0.6222 | 0.249 | 0.7839 | 0.7626 |
| 0.6234 | 0.5688 | 0.7879 | 0.5215 |
| 0.627 | 0.3901 | 0.7896 | 0.6534 |
| 0.6388 | 0.1721 | 0.8387 | 0.6015 |
| 0.6456 | 0.5796 | 0.847 | 0.5903 |
| 0.6626 | 0.1694 | 0.8515 | 0.3716 |
| 0.6881 | 0.6226 | 0.8519 | 0.5055 |
| 0.7002 | 0.7856 | 0.8549 | 0.347 |
| 0.7053 | -0.3431 | 0.8688 | 0.6768 |
| 0.7081 | 0.3667 | 0.9151 | 1.0848 |
| 0.7147 | 0.1125 | 0.9316 | 0.2662 |
| 0.7173 | 0.1735 | 0.9497 | 1.136 |
| 0.7213 | 0.417 | 0.9555 | 1.4863 |
| 0.722 | 0.5182 | 0.9602 | 0.1374 |
| 0.7252 | 0.0016 | 0.9639 | 0.7737 |
| 0.7312 | 0.4311 | 0.9756 | 1.164 |
| 0.75 | 0.4398 | 0.976 | 1.348 |
| 0.7657 | 0.7677 | 0.9833 | 0.9005 |
| 0.7685 | 0.7686 | 1.0076 | 0.3774 |
| 0.7691 | 0.2748 | 1.0169 | 0.9675 |
| 0.7974 | 0.3725 | 1.03 | 1.4796 |
| 0.7982 | 0.5996 | 1.0416 | 0.9306 |
| 0.8238 | 0.6413 | 1.0484 | 0.487 |
| 0.8359 | 1.3193 | 1.0512 | 1.2144 |
| 0.8562 | 0.7154 | 1.0784 | 1.3389 |
| 0.8814 | 0.5348 | 1.1037 | 0.2124 |
| 0.9039 | 0.6497 | 1.1196 | 1.0014 |
| 0.908 | 1.0929 | 1.1411 | 0.8039 |
| 0.9222 | 1.3032 | 1.1466 | 0.8541 |
| 0.9242 | 0.4717 | 1.1575 | 1.1619 |
| 0.9327 | 0.7512 | 1.1635 | 0.2907 |
| 0.9557 | 0.9825 | 1.1646 | 1.3907 |
| 0.9707 | 1.1641 | 1.1716 | 1.5641 |
| 0.9721 | 0.3811 | 1.177 | 1.0473 |
| 0.984 | 0.7498 | 1.1788 | 1.1267 |
| 0.9911 | 1.0551 | 1.1934 | 1.574 |
| 0.9939 | 1.2278 | 1.2022 | 0.8878 |
| 1.0004 | 0.8713 | 1.2142 | 0.6908 |
| 1.0128 | 0.9697 | 1.2393 | 0.9697 |
| 1.0167 | 1.0453 | 1.2492 | 1.0187 |
| 1.0212 | 1.2163 | 1.2561 | 0.5606 |
| 1.0301 | 0.4198 | 1.2645 | 1.6495 |
| 1.0335 | 0.6145 | 1.2727 | 1.3829 |
| 1.0796 | 0.8705 | 1.2863 | 0.4902 |
| 1.0886 | 1.2512 | 1.2895 | 0.9134 |
| 1.0932 | 0.8848 | 1.3026 | 1.3919 |
| 1.1025 | 1.3842 | 1.3134 | 1.5566 |
| 1.108 | 1.0822 | 1.3161 | 1.5401 |
| 1.1316 | 1.5509 | 1.3191 | 1.8269 |
| 1.1623 | 1.1316 | 1.3282 | 1.6251 |
| 1.1692 | 1.0182 | 1.3346 | 1.8763 |
| 1.1733 | 1.1838 | 1.342 | 1.1165 |
| 1.185 | 1.061 | 1.3493 | 0.9622 |
| 1.2022 | 2.0241 | 1.3521 | 1.0209 |
| 1.2058 | 1.6965 | 1.3524 | 0.6926 |
| 1.2096 | 1.1779 | 1.3789 | 1.7919 |
| 1.2102 | 1.852 | 1.4022 | 1.6923 |
| 1.2338 | 0.0986 | 1.4115 | 1.3872 |
| 1.2492 | 1.4212 | 1.4191 | 1.9558 |
| 1.2585 | 1.5499 | 1.4296 | 1.4254 |
| 1.2825 | 1.1826 | 1.4404 | 0.6378 |
| 1.2837 | 0.854 | 1.4509 | 0.6674 |
| 1.2971 | 1.8045 | 1.4576 | 1.8108 |
| 1.3048 | 0.7111 | 1.4596 | 1.6491 |
| 1.3266 | 0.8422 | 1.4627 | 1.2768 |
| 1.3474 | 1.9219 | 1.4919 | 1.3232 |
| 1.3554 | 0.6544 | 1.5054 | 1.9307 |
| 1.3683 | 1.3392 | 1.5067 | 1.5773 |
| 1.3761 | 0.7392 | 1.5104 | 0.8779 |
| 1.3943 | 2.056 | 1.5365 | 1.6491 |
| 1.3987 | 2.1728 | 1.5418 | 2.4296 |
| 1.4228 | 1.5671 | 1.5463 | 2.0398 |
| 1.4238 | 1.5476 | 1.5493 | 0.3865 |
| 1.4261 | 1.7092 | 1.5531 | 1.4757 |
| 1.4417 | 2.3245 | 1.5539 | 2.412 |
| 1.4436 | 1.5033 | 1.5696 | 1.9533 |
| 1.4637 | 0.9883 | 1.5721 | 0.1703 |
| 1.4716 | 0.5617 | 1.5744 | 2.1045 |
| 1.4993 | 1.1627 | 1.5749 | 0.6737 |
| 1.5 | 1.1188 | 1.5783 | 1.8951 |
| 1.5017 | 0.9272 | 1.5796 | 1.8753 |
| 1.5059 | 0.8594 | 1.5888 | 0.8298 |
| 1.5073 | 1.775 | 1.5942 | 2.8712 |
| 1.5211 | 1.9602 | 1.5964 | 0.7957 |
| 1.5325 | 1.6612 | 1.6081 | 1.8559 |
| 1.5348 | 1.7885 | 1.6195 | 1.189 |
| 1.5432 | 0.9078 | 1.6477 | 1.8569 |
| 1.5462 | 1.4804 | 1.6483 | 1.6306 |
| 1.5576 | 0.5406 | 1.6807 | 3.3665 |
| 1.5649 | 1.523 | 1.6904 | 2.2207 |
| 1.581 | 0.8706 | 1.7047 | 1.3093 |
| 1.5866 | 1.0095 | 1.7069 | 1.5935 |
| 1.5936 | 1.3747 | 1.7154 | 1.1852 |
| 1.5949 | 2.5095 | 1.7351 | 1.2876 |
| 1.6102 | 2.2912 | 1.7409 | 1.7829 |
| 1.6198 | 0.9422 | 1.745 | 1.8598 |
| 1.6306 | 0.7208 | 1.7491 | 2.4844 |
| 1.6327 | 2.417 | 1.7607 | 0.6859 |
| 1.6352 | 2.141 | 1.767 | 1.2579 |
| 1.6564 | 0.5192 | 1.7688 | 1.5568 |
| 1.6626 | 2.3126 | 1.7832 | 0.7176 |
| 1.6746 | 3.4331 | 1.8046 | 1.9929 |
| 1.6942 | 1.4385 | 1.8317 | 1.1107 |
| 1.6992 | 2.3087 | 1.8336 | 2.0119 |
| 1.7009 | 1.6335 | 1.8521 | 2.4376 |
| 1.7228 | 2.3411 | 1.8531 | 2.5701 |
| 1.731 | 1.5024 | 1.8564 | 0.8219 |
| 1.7328 | 1.2375 | 1.8772 | 1.3149 |
| 1.7382 | 0.9142 | 1.8776 | 0.9922 |
| 1.7506 | 2.234 | 1.8779 | 1.5021 |
| 1.7528 | 1.2802 | 1.8919 | 1.6282 |
| 1.7804 | 1.3115 | 1.9091 | 1.9319 |
| 1.792 | 0.8431 | 1.919 | 1.8438 |
| 1.8031 | 1.132 | 1.9258 | 0.1577 |
| 1.8091 | 1.8951 | 1.9295 | 2.1159 |
| 1.824 | 1.5843 | 1.9382 | 0.2094 |
| 1.8253 | 2.1149 | 1.9478 | 1.8725 |
| 1.8328 | 2.8079 | 1.9718 | 1.772 |
| 1.8391 | 1.4418 | 1.979 | 1.6574 |
| 1.8397 | 3.6736 | 1.9897 | 2.5266 |
| 1.8561 | 1.876 | 1.99 | 0.3261 |
| 1.8624 | 1.8715 | 1.9924 | 0.5849 |
| 1.8635 | 2.7524 | 1.9924 | 3.109 |
| 1.8776 | 1.8405 | 1.9946 | 3.3691 |
| 1.8947 | -0.1464 | 1.9993 | 1.3455 |
| 1.8976 | 3.2735 | 2.0004 | 3.2437 |
| 1.901 | 1.6657 | 2.0027 | 2.1975 |
| 1.9032 | 2.5548 | 2.0194 | 1.5014 |
| 1.9032 | 0.5739 | 2.0405 | 2.4594 |
| 1.9109 | 0.9049 | 2.0412 | 2.291 |
| 1.9147 | 2.6872 | 2.0596 | 1.014 |
| 1.9172 | 2.201 | 2.0611 | 1.1425 |
| 1.9361 | 1.4596 | 2.0619 | 2.8421 |
| 1.944 | -0.5357 | 2.0668 | 2.4545 |
| 1.9466 | 1.1351 | 2.0706 | 1.9668 |
| 1.9561 | 1.8202 | 2.0715 | 0.6021 |
| 1.9665 | 1.6945 | 2.0729 | 1.4868 |
| 1.9849 | 0.9945 | 2.0797 | 1.3333 |
| 1.9861 | 1.1035 | 2.0834 | 0.6449 |
| 2.0025 | 1.2551 | 2.0841 | 2.01 |
| 2.0035 | 1.8863 | 2.1011 | 1.1299 |
| 2.0068 | 0.4265 | 2.1124 | -0.3577 |
| 2.0141 | 0.3322 | 2.1133 | 2.2778 |
| 2.0214 | 2.5621 | 2.1167 | 1.7388 |
| 2.025 | 2.8528 | 2.1236 | 2.2022 |
| 2.0276 | 1.2582 | 2.1254 | 1.6394 |
| 2.047 | 1.3287 | 2.1267 | 1.9029 |
| 2.0729 | 2.1952 | 2.1309 | 1.6713 |
| 2.0744 | 0.9006 | 2.1314 | 2.9094 |
| 2.0785 | 1.3477 | 2.1526 | -0.0233 |
| 2.0806 | 1.1898 | 2.154 | 2.3681 |
| 2.0866 | 0.968 | 2.1574 | 1.887 |
| 2.1007 | 1.4747 | 2.1796 | 0.8617 |
| 2.1023 | 1.5711 | 2.181 | 1.7477 |
| 2.1076 | 0.6954 | 2.1853 | 1.4932 |
| 2.1139 | 1.5239 | 2.1921 | 1.9713 |
| 2.1159 | 3.0144 | 2.1965 | 3.6817 |
| 2.1187 | 1.2816 | 2.2023 | 1.8473 |
| 2.1279 | 1.2982 | 2.2027 | 1.0353 |
| 2.1364 | 1.6237 | 2.209 | 2.1589 |
| 2.1486 | 2.5416 | 2.2232 | 0.8752 |
| 2.155 | 3.2047 | 2.2286 | 3.4427 |
| 2.1644 | 1.5441 | 2.2373 | 1.3078 |
| 2.1784 | 1.5059 | 2.2548 | 2.8398 |
| 2.1844 | 1.9489 | 2.2579 | 2.277 |
| 2.189 | 1.8244 | 2.2724 | 0.2059 |
| 2.1948 | 0.039 | 2.2778 | 1.4335 |
| 2.2021 | 0.4047 | 2.3021 | -1.0355 |
| 2.2181 | 0.4074 | 2.304 | 0.345 |
| 2.2313 | 2.5472 | 2.3372 | -0.6873 |
| 2.2816 | 1.2351 | 2.3392 | -0.2338 |
| 2.2894 | 2.3363 | 2.3456 | 0.7214 |
| 2.2982 | 2.0283 | 2.3463 | 2.1055 |
| 2.3009 | 3.2318 | 2.3627 | 1.8261 |
| 2.3288 | 0.9805 | 2.376 | 2.0221 |
| 2.3294 | 1.2685 | 2.4167 | 0.9282 |
| 2.3338 | 1.7572 | 2.4471 | 1.408 |
| 2.3415 | 1.3199 | 2.4912 | 2.2059 |
| 2.3505 | 2.1982 | 2.4932 | 2.6873 |
| 2.3517 | 2.1564 | 2.5273 | 0.4547 |
| 2.3527 | 1.3642 | 2.5288 | 2.3165 |
| 2.3545 | 0.8798 | 2.5331 | 2.7289 |
| 2.3604 | 3.9663 | 2.5553 | 0.3618 |
| 2.3694 | 0.676 | 2.561 | 1.1335 |
| 2.3697 | 0.2461 | 2.5679 | 2.9219 |
| 2.3757 | 2.5362 | 2.5704 | 0.8779 |
| 2.3788 | 2.3431 | 2.5795 | 0.8287 |
| 2.3881 | 0.1935 | 2.5927 | 1.1142 |
| 2.3912 | 0.4117 | 2.5951 | 0.1435 |
| 2.3998 | 2.1593 | 2.6099 | 2.198 |
| 2.4011 | 1.425 | 2.617 | 0.0474 |
| 2.407 | 1.7463 | 2.6354 | 1.9543 |
| 2.424 | 1.5022 | 2.6415 | 0.7905 |
| 2.4288 | 1.125 | 2.6762 | -0.2067 |
| 2.4299 | 1.2283 | 2.6821 | 3.6514 |
| 2.4306 | 1.3263 | 2.6832 | -0.6918 |
| 2.4487 | 1.5557 | 2.6841 | 1.4471 |
| 2.4548 | 2.7076 | 2.7198 | 0.5381 |
| 2.463 | 1.4301 | 2.7295 | 1.3383 |
| 2.4801 | 1.8359 | 2.7353 | 1.7736 |
| 2.4839 | 0.6259 | 2.7619 | 2.7863 |
| 2.4886 | 2.0452 | 2.7756 | 1.9551 |
| 2.5048 | 3.5383 | 2.7759 | 2.2776 |
| 2.5161 | 0.5962 | 2.777 | 0.9865 |
| 2.5233 | 2.3535 | 2.7774 | 1.236 |
| 2.5247 | 2.5161 | 2.7821 | 3.2396 |
| 2.5318 | 3.4042 | 2.7828 | 1.3143 |
| 2.5394 | 1.19 | 2.7861 | 2.6156 |
| 2.541 | 2.5922 | 2.8061 | 1.8566 |
| 2.5482 | 1.1123 | 2.8192 | 1.3471 |
| 2.5628 | 1.3365 | 2.8317 | 0.2716 |
| 2.5662 | 1.4664 | 2.8361 | 0.4673 |
| 2.5726 | 1.3908 | 2.8381 | 1.7706 |
| 2.5735 | -0.8527 | 2.845 | 1.5005 |
| 2.5782 | 1.4495 | 2.8459 | 0.3075 |
| 2.5875 | 1.2863 | 2.8618 | 2.9966 |
| 2.588 | 0.8967 | 2.8794 | -0.9359 |
| 2.6075 | 1.9824 | 2.8798 | 0.6029 |
| 2.6252 | 1.4675 | 2.8967 | 0.3512 |
| 2.6416 | 0.7039 | 2.9035 | 3.2982 |
| 2.6667 | 0.9689 | 2.9061 | 1.3727 |
| 2.6823 | 2.9901 | 2.9151 | 1.4978 |
| 2.6929 | 1.9333 | 2.9189 | -0.0856 |
| 2.6994 | 1.1257 | 2.9376 | 0.7893 |
| 2.7089 | 0.9677 | 2.9393 | 1.3522 |
| 2.7101 | 0.8526 | 2.9464 | 0.5629 |
| 2.7308 | 0.2441 | 2.9495 | -1.378 |
| 2.7313 | 1.3517 | 2.9716 | 0.0827 |
| 2.7324 | 1.1851 | 2.9753 | 0.1659 |
| 2.734 | 1.5918 | 2.9952 | -0.8783 |
| 2.7467 | -0.0396 | 3.0034 | 0.9411 |
| 2.747 | 0.764 | 3.004 | 2.0805 |
| 2.7774 | 1.3442 | 3.0093 | -0.8049 |
| 2.7989 | 0.744 | 3.0142 | 0.391 |
| 2.8153 | 1.2484 | 3.0216 | 0.943 |
| 2.8292 | 0.1334 | 3.028 | 0.0381 |
| 2.831 | 1.8835 | 3.0587 | -0.2705 |
| 2.832 | 1.994 | 3.0615 | 2.6995 |
| 2.8564 | 1.7745 | 3.0717 | -1.3826 |
| 2.8717 | 2.2899 | 3.077 | 1.4174 |
| 2.8907 | 1.0092 | 3.0817 | -0.75 |
| 2.9054 | -0.3105 | 3.0852 | -0.7386 |
| 2.9233 | 0.9354 | 3.0853 | -1.4995 |
| 2.9321 | 0.8246 | 3.0863 | -0.5001 |
| 2.9404 | 2.958 | 3.0959 | 0.463 |
| 2.9494 | 0.7599 | 3.1112 | 0.9426 |
| 2.9506 | 0.6574 | 3.1131 | -0.2029 |
| 2.9638 | 0.854 | 3.1212 | 0.4634 |
| 2.9813 | 2.9158 | 3.1252 | -1.1836 |
| 2.9893 | -0.0994 | 3.1268 | -0.6765 |
| 2.99 | 1.9617 | 3.1282 | 1.2986 |
| 3.0033 | 1.3675 | 3.1405 | -0.6426 |
| 3.012 | 0.3666 | 3.1438 | 0.0274 |
| 3.0243 | 3.1159 | 3.1727 | -1.9664 |
| 3.0363 | -0.1166 | 3.2137 | 0.4343 |
| 3.0418 | -0.9876 | 3.2183 | 1.5741 |
| 3.0466 | -1.1027 | 3.2643 | -0.525 |
| 3.0846 | -0.2942 | 3.2685 | -0.4383 |
| 3.0849 | 1.2921 | 3.2774 | 0.1432 |
| 3.0952 | 0.5632 | 3.2879 | -1.0605 |
| 3.1028 | 0.6892 | 3.2971 | -1.3466 |
| 3.1097 | 0.971 | 3.3023 | 0.2083 |
| 3.1136 | 1.0352 | 3.3061 | 0.6642 |
| 3.114 | 0.1692 | 3.309 | -1.8483 |
| 3.1567 | 1.3871 | 3.3099 | -0.8216 |
| 3.1588 | -0.0437 | 3.3187 | 1.0247 |
| 3.1688 | 0.8623 | 3.3238 | -1.0438 |
| 3.1753 | 0.895 | 3.332 | -2.419 |
| 3.1846 | 0.5927 | 3.3391 | 2.8416 |
| 3.194 | -1.0228 | 3.341 | 2.1599 |
| 3.1954 | -2.846 | 3.342 | -1.2733 |
| 3.2023 | -2.9643 | 3.3616 | -0.9914 |
| 3.2023 | -1.4183 | 3.3628 | -0.5003 |
| 3.2078 | -1.6476 | 3.3636 | 0.0734 |
| 3.2172 | -0.6335 | 3.3666 | -2.7106 |
| 3.2316 | 1.8168 | 3.3707 | 0.4308 |
| 3.2519 | 1.1086 | 3.3837 | 1.5435 |
| 3.2524 | -3.706 | 3.3949 | -3.1568 |
| 3.2545 | -0.4784 | 3.3993 | -3.1958 |
| 3.2589 | 0.204 | 3.4341 | 0.7918 |
| 3.2614 | -0.2392 | 3.4379 | -0.8333 |
| 3.267 | 1.2291 | 3.4499 | -4.6579 |
| 3.2882 | -0.1583 | 3.4511 | -1.4571 |
| 3.2897 | -1.8586 | 3.4688 | -1.0246 |
| 3.2974 | 2.4084 | 3.4742 | -0.6278 |
| 3.2989 | 1.5852 | 3.507 | -1.6362 |
| 3.2995 | -1.2105 | 3.5078 | -2.9792 |
| 3.3011 | 0.2073 | 3.5137 | 0.1971 |
| 3.3075 | 0.8081 | 3.5284 | -0.8581 |
| 3.309 | -2.3605 | 3.5456 | -3.9438 |
| 3.3265 | 0.0079 | 3.5567 | -3.4912 |
| 3.3321 | -1.5099 | 3.5641 | 3.7084 |
| 3.339 | 1.0414 | 3.6058 | 1.0986 |
| 3.3517 | -0.8276 | 3.6094 | -2.8512 |
| 3.3643 | -3.4862 | 3.6145 | -2.5941 |
| 3.365 | -1.9434 | 3.6152 | -0.3299 |
| 3.3742 | -2.6709 | 3.6272 | -1.4338 |
| 3.3776 | -0.3394 | 3.6427 | -2.3689 |
| 3.392 | 2.0743 | 3.6448 | -3.3914 |
| 3.3971 | -1.652 | 3.6476 | -5.6204 |
| 3.4014 | -1.9188 | 3.662 | -1.3256 |
| 3.4064 | -0.1281 | 3.6653 | -1.1851 |
| 3.4122 | -0.7638 | 3.6664 | -3.5966 |
| 3.4195 | 0.1777 | 3.6699 | -1.9446 |
| 3.421 | -1.0705 | 3.6751 | -1.5325 |
| 3.4327 | -1.9199 | 3.6777 | -2.9507 |
| 3.4455 | -2.4991 | 3.6812 | -3.7134 |
| 3.4623 | -1.703 | 3.6831 | -0.1963 |
| 3.4687 | -0.8684 | 3.6939 | 2.2823 |
| 3.4706 | 0.8904 | 3.7049 | -1.2888 |
| 3.4891 | -4.738 | 3.7069 | -2.8601 |
| 3.4894 | -1.2439 | 3.7168 | 0.5062 |
| 3.4906 | -1.8556 | 3.7271 | -1.1444 |
| 3.4909 | -1.5139 | 3.7361 | -2.8442 |
| 3.4967 | -1.5547 | 3.7398 | 0.3481 |
| 3.5144 | -2.4034 | 3.7717 | -4.1648 |
| 3.5195 | -2.65 | 3.7777 | -2.6229 |
| 3.5259 | 0.6176 | 3.7842 | -0.7429 |
| 3.5336 | -2.1745 | 3.7981 | -0.7077 |
| 3.584 | -2.3669 | 3.8011 | -6.0504 |
| 3.5853 | -3.2381 | 3.8256 | -3.1969 |
| 3.5884 | -2.3653 | 3.8703 | -2.5113 |
| 3.6036 | -0.0538 | 3.8779 | -1.7117 |
| 3.62 | 0.6381 | 3.8803 | -4.7338 |
| 3.6273 | -1.0857 | 3.8813 | -3.6101 |
| 3.6318 | -2.6374 | 3.8993 | -6.455 |
| 3.639 | -1.5886 | 3.9204 | -4.2352 |
| 3.6403 | 0.7478 | 3.9211 | -4.9308 |
| 3.6408 | 0.5829 | 3.9259 | -5.8494 |
| 3.6673 | -1.8605 | 3.9371 | -3.3253 |
| 3.6754 | 1.3043 | 3.9445 | -3.6521 |
| 3.6757 | -1.529 | 3.9487 | -3.235 |
| 3.6787 | -2.9222 | 3.95 | -2.5245 |
| 3.6968 | -5.1991 | 3.9507 | -2.9442 |
| 3.7192 | -1.3985 | 3.9509 | -3.6942 |
| 3.723 | -4.9091 | 3.9553 | -1.7628 |
| 3.7368 | -2.6638 | 3.9592 | -4.5534 |
| 3.7522 | -1.0442 | 3.969 | -3.6418 |
| 3.7643 | -1.2966 | 4.0011 | -1.4965 |
| 3.7734 | 1.1628 | 4.0163 | -4.2003 |
| 3.7773 | -0.7124 | 4.0171 | -6.2429 |
| 3.7958 | -1.4119 | 4.0258 | -0.9441 |
| 3.8023 | -2.9964 | 4.0379 | -3.088 |
| 3.8162 | -2.2062 | 4.039 | -4.4305 |
| 3.8357 | -5.8937 | 4.0733 | -3.6844 |
| 3.8371 | -1.9873 | 4.0866 | -0.9098 |
| 3.8753 | -0.6624 | 4.0873 | -1.9935 |
| 3.8785 | -4.4885 | 4.091 | -4.6581 |
| 3.8834 | -1.2922 | 4.0941 | -1.4428 |
| 3.8988 | -2.5026 | 4.0962 | -2.7529 |
| 3.9244 | -0.6898 | 4.0991 | -2.5955 |
| 3.9255 | -4.0083 | 4.1098 | -1.9417 |
| 3.9398 | -3.2683 | 4.1136 | -4.6336 |
| 3.9496 | -2.5406 | 4.1236 | -2.3236 |
| 3.9506 | -0.6031 | 4.1242 | -1.6633 |
| 3.9549 | -4.5333 | 4.1374 | -4.4783 |
| 3.9622 | -6.0216 | 4.1493 | -4.592 |
| 3.9862 | -1.4274 | 4.1522 | -2.238 |
| 4.0109 | -1.101 | 4.1608 | -2.3613 |
| 4.0179 | -2.5404 | 4.1625 | -3.2644 |
| 4.0268 | -1.9135 | 4.173 | -4.3929 |
| 4.0459 | -6.6888 | 4.1961 | -1.4266 |
| 4.0479 | -4.9192 | 4.218 | -5.2801 |
| 4.0481 | -4.8442 | 4.2185 | -3.1869 |
| 4.0499 | -4.1002 | 4.2384 | -4.8872 |
| 4.0571 | -0.3915 | 4.2581 | -7.7165 |
| 4.0947 | -4.0692 | 4.2623 | 0.3676 |
| 4.0972 | -2.1201 | 4.2698 | -3.034 |
| 4.0996 | -3.4772 | 4.2765 | -2.0171 |
| 4.1037 | -4.2162 | 4.2969 | -5.3134 |
| 4.1122 | -3.1187 | 4.2981 | -4.594 |
| 4.1176 | -3.1269 | 4.3044 | -2.2358 |
| 4.1227 | -1.6159 | 4.3259 | -4.6176 |
| 4.1431 | -4.8085 | 4.3294 | -2.6855 |
| 4.1445 | -2.3824 | 4.3383 | -1.8382 |
| 4.2116 | -7.121 | 4.3426 | -3.33 |
| 4.2425 | -5.172 | 4.3497 | -4.159 |
| 4.2584 | -2.4694 | 4.3806 | -3.4471 |
| 4.2585 | -6.8208 | 4.3975 | -5.8764 |
| 4.2622 | -4.3483 | 4.4036 | -6.1719 |
| 4.2671 | -4.8391 | 4.4081 | -2.9777 |
| 4.3054 | -5.5968 | 4.4103 | -5.4022 |
| 4.3176 | -1.6704 | 4.4121 | -5.5791 |
| 4.3452 | 0.2345 | 4.4161 | -2.9331 |
| 4.3508 | -5.8374 | 4.4203 | -4.0888 |
| 4.3583 | -4.1998 | 4.4299 | -4.0879 |
| 4.371 | -3.0498 | 4.4384 | -3.2589 |
| 4.372 | -7.3828 | 4.4517 | -4.8061 |
| 4.382 | -1.9297 | 4.4681 | -5.5887 |
| 4.3836 | -6.6172 | 4.4726 | -2.4367 |
| 4.3946 | -6.1668 | 4.4898 | -4.2048 |
| 4.3948 | -2.1968 | 4.4907 | -6.773 |
| 4.3977 | -2.3581 | 4.4928 | -3.2744 |
| 4.4146 | -4.426 | 4.5003 | -0.7099 |
| 4.4315 | -4.6744 | 4.5028 | -1.9771 |
| 4.4319 | -4.9709 | 4.5075 | -3.0633 |
| 4.4477 | -2.2035 | 4.5366 | -3.7231 |
| 4.4484 | -3.8535 | 4.5676 | -3.3861 |
| 4.46 | -4.8363 | 4.6191 | -4.7031 |
| 4.4842 | -5.2215 | 4.6219 | -3.3069 |
| 4.4946 | -3.7393 | 4.6353 | -6.2237 |
| 4.5017 | -4.6623 | 4.6454 | -6.8997 |
| 4.5177 | -4.4979 | 4.6572 | -2.6205 |
| 4.521 | -5.8563 | 4.7201 | -5.1417 |
| 4.5254 | -1.5586 | 4.7616 | -5.8709 |
| 4.5276 | -2.9659 | 4.7942 | -6.4643 |
| 4.5282 | -3.4283 | 4.7966 | -7.5439 |
| 4.536 | -3.6108 | 4.8034 | -6.7316 |
| 4.5368 | -4.3676 | 4.8276 | -4.4155 |
| 4.5467 | -4.2801 | 4.8595 | -6.0845 |
| 4.5471 | -5.9235 | 4.8651 | -1.7411 |
| 4.5509 | -6.5259 | 4.8663 | -4.6911 |
| 4.5669 | -8.241 | 4.8724 | -5.2067 |
| 4.5847 | -4.5211 | 4.8774 | -7.0729 |
| 4.598 | -4.4608 | 4.8781 | -4.7063 |
| 4.6024 | -4.0249 | 4.8867 | -6.8442 |
| 4.6104 | -0.7982 | 4.8905 | -4.8966 |
| 4.6399 | -8.199 | 4.9015 | -0.6115 |
| 4.6449 | -3.2692 | 4.9196 | -5.1811 |
| 4.6573 | -5.0843 | 4.9216 | -4.5817 |
| 4.6694 | -5.2115 | 4.9229 | -4.9482 |
| 4.6707 | -3.9481 | 4.9484 | -5.282 |
| 4.6754 | -5.317 | 4.9587 | -4.6449 |
| 4.6807 | -4.3575 | 4.9698 | -7.2013 |
| 4.7134 | -6.2988 | 4.9769 | -8.2436 |
| 4.7216 | -4.7819 | 4.9963 | -9.3514 |
| 4.7393 | -6.813 | 5.0001 | -6.2086 |
| 4.7573 | -3.5036 | 5.0216 | -4.6291 |
| 4.7888 | -5.4818 | 5.0332 | -3.2836 |
| 4.7991 | -0.6766 | 5.0348 | -11.3964 |
| 4.7991 | -6.7513 | 5.0529 | 0.1864 |
| 4.8175 | -1.7844 | 5.0535 | 3.3788 |
| 4.8177 | -0.7928 | 5.0983 | 0.4551 |
| 4.825 | -3.5227 | 5.1114 | 0.025 |
| 4.8259 | -4.4544 | 5.1264 | 3.0341 |
| 4.8359 | -9.6225 | 5.1317 | -7.2641 |
| 4.8391 | -9.901 | 5.14 | -8.6154 |
| 4.8445 | -5.7422 | 5.1468 | 0.6028 |
| 4.8571 | -9.8686 | 5.1588 | -7.7819 |
| 4.863 | -6.6496 | 5.1618 | 1.0308 |
| 4.8877 | -5.4514 | 5.1717 | -1.4958 |
| 4.9004 | -4.0455 | 5.1736 | -9.4612 |
| 4.9298 | -6.819 | 5.1854 | -0.1311 |
| 4.9456 | -5.2606 | 5.189 | 4.6617 |
| 4.9458 | -9.9275 | 5.19 | -0.8099 |
| 4.9493 | -5.6859 | 5.1998 | 3.2688 |
| 4.9727 | -5.9576 | 5.2001 | -3.7671 |
| 4.98 | -3.2061 | 5.2003 | -3.1879 |
| 4.9856 | -3.25 | 5.2005 | -3.7897 |
| 4.986 | -2.9212 | 5.2014 | -5.488 |
| 5.0018 | -6.2268 | 5.2043 | 2.2898 |
| 5.0028 | -4.138 | 5.2275 | 4.1691 |
| 5.0092 | -5.8433 | 5.2503 | -11.5106 |
| 5.02 | -5.4118 | 5.2597 | -1.6726 |
| 5.0202 | -5.2337 | 5.2753 | 1.3417 |
| 5.0208 | -5.8284 | 5.2777 | 0.9289 |
| 5.0391 | -4.5408 | 5.2783 | -11.7739 |
| 5.0401 | -5.7657 | 5.2811 | -0.9121 |
| 5.047 | -4.25 | 5.3103 | 0.2677 |
| 5.0471 | -4.9041 | 5.3142 | 3.7685 |
| 5.051 | -3.9097 | 5.3155 | -2.2952 |
| 5.1098 | -3.6078 | 5.3174 | -13.4535 |
| 5.1128 | -2.4476 | 5.335 | -10.5133 |
| 5.117 | -2.4083 | 5.3651 | 0.0947 |
| 5.1528 | -5.5802 | 5.3675 | -4.5573 |
| 5.1602 | -3.9668 | 5.3818 | 3.9849 |
| 5.1642 | -1.1243 | 5.3836 | -5.9566 |
| 5.1674 | -3.2491 | 5.3861 | 1.9898 |
| 5.1705 | -3.4543 | 5.3893 | -0.7414 |
| 5.1837 | -10.2494 | 5.4086 | -2.2067 |
| 5.2162 | -5.7407 | 5.4091 | -14.6916 |
| 5.2329 | -7.9959 | 5.4305 | -0.0901 |
| 5.2515 | -4.0937 | 5.4318 | 1.5069 |
| 5.2543 | -6.2504 | 5.4508 | 1.6434 |
| 5.2626 | -5.2892 | 5.4525 | -7.5393 |
| 5.2633 | -8.8538 | 5.4847 | -5.4213 |
| 5.267 | -3.95 | 5.498 | -0.1014 |
| 5.2829 | -8.7158 | 5.518 | 2.1241 |
| 5.2848 | -6.7737 | 5.5236 | 1.3333 |
| 5.2911 | -1.7994 | 5.5254 | -2.2376 |
| 5.3384 | -5.5807 | 5.5443 | -3.3645 |
| 5.3414 | -4.4084 | 5.5565 | 0.0687 |
| 5.3425 | -3.4825 | 5.5571 | 0.6666 |
| 5.354 | -4.2353 | 5.5798 | 3.8603 |
| 5.3592 | -2.194 | 5.5798 | 4.3605 |
| 5.3646 | -2.441 | 5.5807 | -6.6211 |
| 5.3732 | -3.1259 | 5.5991 | -5.5456 |
| 5.3827 | -0.6508 | 5.6084 | -3.0973 |
| 5.4122 | -7.3682 | 5.6178 | -7.8579 |
| 5.4238 | -6.0693 | 5.6251 | 0.0035 |
| 5.4282 | -6.1926 | 5.6305 | 0.2937 |
| 5.4431 | -2.2652 | 5.6322 | -9.645 |
| 5.4437 | -4.3474 | 5.6622 | 3.4281 |
| 5.4552 | -4.2957 | 5.6635 | -3.79 |
| 5.4778 | -6.8704 | 5.668 | -5.8309 |
| 5.493 | -1.8554 | 5.6743 | -1.1794 |
| 5.4994 | -2.9082 | 5.6839 | 1.6843 |
| 5.5094 | -3.3163 | 5.6843 | 3.7366 |
| 5.5115 | -9.5481 | 5.6851 | -7.2894 |
| 5.5124 | 0.0258 | 5.6928 | -1.9922 |
| 5.532 | -3.6951 | 5.7371 | -12.098 |
| 5.5364 | -5.6645 | 5.7457 | 0.9582 |
| 5.5568 | -2.0951 | 5.7479 | -2.4373 |
| 5.565 | -0.4633 | 5.7482 | -2.0016 |
| 5.5716 | -4.6696 | 5.7568 | -4.2624 |
| 5.5738 | -6.1455 | 5.7581 | -5.1638 |
| 5.5739 | -5.6171 | 5.7742 | -4.9897 |
| 5.5826 | -5.5105 | 5.7759 | 0.1306 |
| 5.5874 | -5.5902 | 5.7804 | 0.0033 |
| 5.592 | -4.1071 | 5.7979 | -5.7174 |
| 5.5975 | -5.7977 | 5.8057 | -3.4522 |
| 5.5979 | -3.2159 | 5.8386 | -14.0881 |
| 5.6039 | -3.351 | 5.8635 | -5.193 |
| 5.607 | -6.1781 | 5.8725 | 0.8485 |
| 5.6282 | -1.8539 | 5.8783 | -1.0917 |
| 5.6329 | -3.5193 | 5.8976 | -1.0647 |
| 5.6455 | 1.8834 | 5.9016 | -4.5855 |
| 5.6491 | -3.4299 | 5.9062 | -5.7064 |
| 5.6614 | -6.2185 | 5.9141 | 0.0555 |
| 5.6706 | -5.3601 | 5.9362 | -0.2443 |
| 5.6748 | -7.3581 | 5.9512 | -0.2294 |
| 5.6763 | -3.549 | 5.953 | -1.0999 |
| 5.6856 | 5.0026 | 5.9585 | 0.0199 |
| 5.6885 | -2.8441 | 5.9796 | -3.7654 |
| 5.6947 | -3.5835 | 5.9803 | -5.0015 |
| 5.7319 | 0.2647 | 5.9816 | -0.1389 |
| 5.7353 | 0.6052 | 5.9837 | -6.8232 |
| 5.737 | -5.886 | 5.9882 | 6.4734 |
| 5.7428 | -7.2902 | 5.9931 | 0.7967 |
| 5.7479 | -2.0552 | 6.0241 | -6.4975 |
| 5.7494 | 0.2834 | 6.0314 | -14.8445 |
| 5.7535 | -3.8322 | 6.0486 | -0.0437 |
| 5.7628 | -5.4329 | 6.0527 | 0.3178 |
| 5.7692 | -6.5162 | 6.0542 | -1.0758 |
| 5.7715 | -2.5571 | 6.0793 | -1.9316 |
| 5.7959 | -3.7616 | 6.0802 | 0.9188 |
| 5.8038 | -2.4513 | 6.0839 | -0.2895 |
| 5.8161 | -3.6144 | 6.102 | -0.0009 |
| 5.8189 | -6.9833 | 6.1237 | 2.3329 |
| 5.8315 | -2.1505 | 6.1306 | -0.5408 |
| 5.8468 | 0.0962 | 6.1516 | -1.9798 |
| 5.8509 | -3.4051 | 6.1582 | -0.0525 |
| 5.8876 | 0.2464 | 6.1602 | 0.0266 |
| 5.9038 | -1.4878 | 6.176 | -6.3161 |
| 5.9085 | -3.41 | 6.1967 | -0.8666 |
| 5.9227 | -6.8953 | 6.197 | 0.2753 |
| 5.9476 | -6.5883 | 6.2047 | -1.5891 |
| 5.954 | -2.5731 | 6.2292 | -0.843 |
| 5.9652 | -1.8532 | 6.257 | -1.0742 |
| 5.9696 | -4.2421 | 6.2572 | -1.2445 |
| 5.9966 | -4.6336 | 6.2856 | -6.9574 |
| 6.0007 | -0.5871 | 6.2958 | 1.3434 |
| 6.0172 | -1.0301 | 6.3133 | 1.0303 |
| 6.0198 | -5.91 | 6.3198 | 0.7026 |
| 6.0245 | -4.3619 | 6.3266 | 2.9734 |
| 6.0335 | 0.7098 | 6.3286 | 1.8477 |
| 6.0557 | 0.0266 | 6.3325 | 2.4125 |
| 6.0647 | -2.8251 | 6.3545 | -1.2172 |
| 6.0811 | 0.9875 | 6.3627 | 0.1943 |
| 6.121 | -1.6005 | 6.3789 | -1.8958 |
| 6.1214 | -1.1654 | 6.3912 | -1.6282 |
| 6.1306 | -2.8148 | 6.3912 | -2.6088 |
| 6.1339 | -3.3049 | 6.3976 | -4.6714 |
| 6.1358 | -5.1124 | 6.3989 | -3.7951 |
| 6.1543 | 0.6506 | 6.4063 | -0.3604 |
| 6.155 | -1.0507 | 6.4377 | 6.9085 |
| 6.1594 | 3.7132 | 6.4402 | -1.5472 |
| 6.1673 | -0.1462 | 6.4472 | 3.3327 |
| 6.1807 | -0.3132 | 6.4854 | -0.2329 |
| 6.1823 | 3.7338 | 6.5021 | 2.5312 |
| 6.1874 | -0.0643 | 6.5321 | 0.9572 |
| 6.2068 | -1.2815 | 6.5408 | -1.3165 |
| 6.2074 | -2.8908 | 6.5443 | 2.7089 |
| 6.208 | 3.699 | 6.5458 | 0.5627 |
| 6.2162 | -4.1293 | 6.5474 | 2.5835 |
| 6.227 | 0.8714 | 6.5492 | 0.0464 |
| 6.2287 | 0.2389 | 6.5643 | 0.2 |
| 6.2361 | -4.917 | 6.5887 | -0.5867 |
| 6.2436 | -0.1784 | 6.5946 | 9.8367 |
| 6.248 | -4.8622 | 6.6033 | -1.542 |
| 6.2577 | 1.214 | 6.6109 | 11.0818 |
| 6.2747 | -0.8278 | 6.6151 | 3.9908 |
| 6.2917 | -0.3799 | 6.616 | 3.4183 |
| 6.2971 | -2.4709 | 6.6191 | 1.6887 |
| 6.2973 | -0.3019 | 6.6205 | 0.3701 |
| 6.3025 | 1.9095 | 6.6276 | -4.4918 |
| 6.3143 | -2.7913 | 6.6408 | -1.9743 |
| 6.3476 | 0.2627 | 6.6434 | 1.5226 |
| 6.3615 | 2.988 | 6.6572 | 2.4246 |
| 6.369 | -1.0494 | 6.6635 | -1.4428 |
| 6.3711 | 3.986 | 6.6651 | -0.0785 |
| 6.3962 | -4.1127 | 6.6762 | 1.4783 |
| 6.4206 | 0.5699 | 6.6867 | 0.1879 |
| 6.4451 | 1.3129 | 6.6909 | -0.9599 |
| 6.4542 | 0.5171 | 6.6951 | 7.7764 |
| 6.4732 | 3.4283 | 6.6964 | 2.1625 |
| 6.4777 | -1.9322 | 6.702 | 4.3384 |
| 6.4778 | 4.2222 | 6.7131 | 5.2463 |
| 6.4863 | 0.1417 | 6.722 | 4.1664 |
| 6.4904 | -0.4593 | 6.7253 | -1.0475 |
| 6.4978 | 1.0184 | 6.7297 | -3.1288 |
| 6.5198 | 1.769 | 6.7427 | 1.9218 |
| 6.5272 | 6.3813 | 6.7489 | -0.8607 |
| 6.5351 | 1.3018 | 6.7819 | 9.5058 |
| 6.5434 | 3.9731 | 6.8177 | 0.3742 |
| 6.5563 | -1.8514 | 6.8317 | 3.8605 |
| 6.5843 | 5.9599 | 6.838 | 5.5379 |
| 6.5919 | -5.7573 | 6.8384 | -0.1576 |
| 6.6128 | 2.4209 | 6.8402 | 3.276 |
| 6.6235 | 3.7681 | 6.8595 | 3.1843 |
| 6.6321 | 6.5195 | 6.8664 | -3.983 |
| 6.6421 | 2.1697 | 6.8752 | -2.4075 |
| 6.6508 | 1.4467 | 6.8883 | 0.1293 |
| 6.6634 | 6.3928 | 6.8912 | 0.2154 |
| 6.6916 | 5.8543 | 6.909 | -1.7589 |
| 6.6927 | 2.8239 | 6.9136 | 1.6992 |
| 6.7026 | 2.4014 | 6.9328 | -0.8838 |
| 6.7125 | 4.7475 | 6.9358 | 7.3458 |
| 6.7229 | 3.5387 | 6.9457 | 4.0526 |
| 6.7528 | 2.0582 | 6.9465 | 10.1094 |
| 6.7541 | -0.1356 | 6.9576 | 1.3613 |
| 6.7598 | 5.0986 | 6.9579 | -2.2063 |
| 6.7661 | 1.7457 | 6.9654 | -5.6252 |
| 6.8013 | 5.652 | 6.975 | 9.4899 |
| 6.8049 | 3.1606 | 6.9953 | 0.3519 |
| 6.8226 | 5.103 | 7.0111 | 12.3617 |
| 6.8267 | 3.6847 | 7.012 | 0.0039 |
| 6.8534 | -0.7159 | 7.0122 | 3.4431 |
| 6.8586 | 4.1852 | 7.0125 | 18.7912 |
| 6.8709 | 0.305 | 7.0154 | 4.0318 |
| 6.8791 | 3.5719 | 7.0267 | 11.2151 |
| 6.8882 | 6.5385 | 7.0469 | -0.3569 |
| 6.8985 | 1.6677 | 7.0473 | 16.0836 |
| 6.9031 | 7.012 | 7.0636 | -8.7163 |
| 6.9413 | 1.3743 | 7.0755 | -9.4489 |
| 6.9492 | 3.6185 | 7.0972 | 2.7264 |
| 6.9668 | -0.4437 | 7.1005 | 5.8661 |
| 6.9715 | 2.2515 | 7.102 | 5.6044 |
| 6.9889 | 7.2076 | 7.1205 | 4.4278 |
| 6.993 | 6.1088 | 7.1249 | 1.1936 |
| 6.9979 | 3.5021 | 7.1305 | -4.1543 |
| 7.0104 | 8.0545 | 7.1317 | 8.3581 |
| 7.0347 | 6.414 | 7.1374 | -3.564 |
| 7.0465 | 2.8635 | 7.1385 | 4.9074 |
| 7.0521 | 3.3453 | 7.1468 | 2.0027 |
| 7.0587 | 2.591 | 7.1668 | 7.2164 |
| 7.0603 | 5.1245 | 7.1741 | 1.154 |
| 7.0635 | 0.2962 | 7.1762 | -0.6136 |
| 7.0751 | 6.227 | 7.2296 | 0.8656 |
| 7.0754 | 1.5816 | 7.2314 | -0.0716 |
| 7.0847 | 2.0657 | 7.2356 | 16.5545 |
| 7.0975 | 5.6557 | 7.2435 | 3.4122 |
| 7.1164 | 12.609 | 7.2476 | -6.0814 |
| 7.1226 | 0.176 | 7.2603 | 5.616 |
| 7.1264 | 3.2024 | 7.264 | -4.9068 |
| 7.14 | 9.0847 | 7.2681 | -1.4065 |
| 7.146 | 10.8587 | 7.2721 | 0.4209 |
| 7.1538 | 2.9019 | 7.2819 | 14.1964 |
| 7.1699 | 8.457 | 7.2913 | 14.3449 |
| 7.1931 | 8.1195 | 7.2939 | 0.6939 |
| 7.1944 | 2.9356 | 7.2998 | -6.0812 |
| 7.196 | -0.3108 | 7.3064 | 3.8272 |
| 7.1967 | 4.6321 | 7.3224 | -0.3574 |
| 7.2053 | 4.7098 | 7.3256 | 10.7854 |
| 7.2081 | 6.3955 | 7.3344 | 2.6951 |
| 7.2125 | 3.4306 | 7.3353 | 3.7053 |
| 7.2127 | 7.6358 | 7.3358 | 5.3237 |
| 7.2146 | 8.9233 | 7.3368 | 15.5196 |
| 7.2343 | 0.6599 | 7.3728 | -3.8946 |
| 7.2421 | 2.4001 | 7.3781 | 11.2576 |
| 7.2476 | 5.1526 | 7.3899 | -6.9453 |
| 7.2609 | -0.2375 | 7.3921 | 9.6731 |
| 7.2917 | 7.9678 | 7.4117 | 2.4603 |
| 7.3021 | 2.8324 | 7.4254 | 15.9989 |
| 7.3035 | 1.666 | 7.4298 | -1.244 |
| 7.3056 | 1.923 | 7.4384 | -5.0446 |
| 7.3151 | 0.2533 | 7.4411 | -1.6994 |
| 7.3222 | 3.1207 | 7.4526 | 15.6992 |
| 7.3282 | 9.2355 | 7.4591 | 0.0799 |
| 7.3294 | 4.058 | 7.46 | 9.3521 |
| 7.3711 | 3.7737 | 7.4656 | 5.9512 |
| 7.375 | 3.7889 | 7.4705 | 2.6851 |
| 7.3855 | 7.1349 | 7.4855 | 13.6585 |
| 7.3863 | 13.4754 | 7.4862 | 0.2653 |
| 7.387 | 9.0029 | 7.5473 | 9.5182 |
| 7.3888 | 9.9928 | 7.5641 | -7.2327 |
| 7.3889 | 6.4055 | 7.582 | -4.8587 |
| 7.4202 | 7.393 | 7.6333 | 13.6352 |
| 7.4543 | 1.8242 | 7.6377 | 8.6228 |
| 7.4743 | 9.9485 | 7.6617 | 2.534 |
| 7.4825 | 9.3987 | 7.6634 | -2.9295 |
| 7.4885 | 3.1484 | 7.6658 | -1.8884 |
| 7.4897 | 2.084 | 7.6772 | 6.3381 |
| 7.4902 | 10.9591 | 7.6794 | -1.7633 |
| 7.4944 | 4.0811 | 7.6845 | -5.3955 |
| 7.4961 | 7.7631 | 7.6997 | 18.4729 |
| 7.506 | 5.8547 | 7.707 | 0.7727 |
| 7.5071 | 5.793 | 7.7206 | -3.3178 |
| 7.5072 | 11.2078 | 7.7219 | 12.1625 |
| 7.5115 | 7.6928 | 7.7388 | 7.3387 |
| 7.5127 | 6.8942 | 7.7596 | 3.1316 |
| 7.5216 | 0.7006 | 7.7621 | 2.458 |
| 7.525 | 5.7657 | 7.7622 | 14.5971 |
| 7.5294 | 9.184 | 7.7766 | 7.0651 |
| 7.5316 | 0.5877 | 7.7823 | 10.1279 |
| 7.536 | 6.0088 | 7.7832 | 13.9265 |
| 7.5396 | 4.4431 | 7.7856 | -9.9637 |
| 7.5502 | 0.4072 | 7.7995 | 16.9864 |
| 7.5696 | 12.4281 | 7.8123 | -5.3094 |
| 7.5762 | 4.3485 | 7.8248 | 3.1826 |
| 7.5762 | 10.81 | 7.8478 | 0.8765 |
| 7.6145 | 8.8411 | 7.849 | 5.5412 |
| 7.6178 | 10.8729 | 7.865 | 7.3858 |
| 7.6201 | 11.419 | 7.8683 | -8.2283 |
| 7.6202 | 9.8818 | 7.8842 | -0.5158 |
| 7.6251 | 13.0635 | 7.8846 | -2.59 |
| 7.6355 | 13.2979 | 7.8866 | 17.8873 |
| 7.6524 | 10.0048 | 7.8875 | 13.2718 |
| 7.6531 | 6.1179 | 7.9002 | 1.8323 |
| 7.6975 | 5.7156 | 7.9281 | 6.2641 |
| 7.7135 | 7.9337 | 7.9303 | 7.5023 |
| 7.7137 | 5.1444 | 7.9498 | 3.9983 |
| 7.7141 | 3.9916 | 7.9502 | -0.198 |
| 7.7223 | 8.6164 | 7.9638 | 1.5408 |
| 7.7231 | 8.9416 | 7.9756 | 10.4015 |
| 7.7442 | 4.0205 | 7.9977 | 9.3619 |
| 7.7567 | 14.0673 | 8.0068 | 5.5024 |
| 7.7589 | 16.8966 | 8.0282 | 4.8153 |
| 7.7716 | 5.5393 | 8.0324 | 11.7728 |
| 7.7851 | 1.8234 | 8.0336 | 9.4677 |
| 7.8028 | 7.9203 | 8.0382 | 8.8612 |
| 7.8055 | 12.775 | 8.0643 | -0.7732 |
| 7.8381 | 7.7465 | 8.0772 | 8.4166 |
| 7.8656 | 6.4105 | 8.0826 | 4.9871 |
| 7.873 | 1.2339 | 8.0847 | 11.5004 |
| 7.8944 | 10.1305 | 8.0949 | 5.4494 |
| 7.8975 | 6.0373 | 8.1051 | 11.1165 |
| 7.9062 | 6.9023 | 8.1083 | 4.8885 |
| 7.9063 | 13.7702 | 8.1399 | 7.8051 |
| 7.9208 | 14.0583 | 8.1503 | 11.1905 |
| 7.9275 | 4.4574 | 8.1511 | 11.3288 |
| 7.9554 | 11.5644 | 8.1532 | 9.7032 |
| 7.9657 | 8.7247 | 8.1808 | 7.0187 |
| 7.9781 | 12.0449 | 8.1843 | 16.1759 |
| 7.9915 | 4.4759 | 8.1892 | 2.3229 |
| 8.0029 | 4.4776 | 8.2248 | 5.2925 |
| 8.0182 | 10.6747 | 8.2327 | 1.8077 |
| 8.0371 | 8.4799 | 8.2508 | 15.9829 |
| 8.0427 | 4.5786 | 8.2524 | 9.5864 |
| 8.0446 | 12.5515 | 8.2669 | 6.5128 |
| 8.06 | 2.0884 | 8.2723 | 5.5157 |
| 8.0787 | 4.0616 | 8.2873 | 9.3095 |
| 8.0827 | 5.6884 | 8.3037 | 6.9254 |
| 8.0912 | 5.0209 | 8.315 | 4.2946 |
| 8.1062 | 7.6882 | 8.3213 | 4.3198 |
| 8.1068 | 7.8275 | 8.3393 | 11.472 |
| 8.1075 | 9.1134 | 8.3522 | 6.3127 |
| 8.1107 | -0.2145 | 8.382 | -0.7172 |
| 8.1194 | 7.8443 | 8.3901 | 5.4406 |
| 8.125 | 6.8688 | 8.3931 | 10.5051 |
| 8.1559 | 4.5231 | 8.4084 | 5.8728 |
| 8.1586 | 8.2597 | 8.422 | 5.1949 |
| 8.1605 | 3.6119 | 8.425 | 8.5628 |
| 8.1643 | 4.1579 | 8.4263 | 2.6014 |
| 8.1663 | 12.9191 | 8.4267 | 5.0527 |
| 8.1767 | 4.9802 | 8.4328 | 5.9089 |
| 8.1782 | 5.3509 | 8.4452 | 13.7987 |
| 8.1876 | 8.1891 | 8.4628 | 3.9928 |
| 8.2019 | 12.6699 | 8.4765 | 2.0385 |
| 8.2029 | 8.6773 | 8.4789 | 6.4979 |
| 8.2197 | 8.6306 | 8.4792 | 6.1866 |
| 8.2603 | 7.1878 | 8.4804 | 6.1845 |
| 8.2841 | 14.8977 | 8.4856 | 9.747 |
| 8.2896 | 12.0744 | 8.4875 | 4.9862 |
| 8.2952 | 10.4734 | 8.4964 | 12.4363 |
| 8.2977 | 6.1007 | 8.5046 | 6.5975 |
| 8.326 | 7.4289 | 8.5165 | 11.7855 |
| 8.3348 | 5.3629 | 8.5262 | 6.726 |
| 8.3384 | 9.9961 | 8.5353 | 6.8501 |
| 8.353 | 10.8077 | 8.5433 | -3.9161 |
| 8.3729 | 8.3591 | 8.5751 | 9.2114 |
| 8.3947 | 11.251 | 8.5788 | 3.0697 |
| 8.3975 | 1.5668 | 8.5795 | 9.9813 |
| 8.3977 | 8.1381 | 8.6001 | 2.4004 |
| 8.4039 | 10.5832 | 8.6228 | 10.5173 |
| 8.4053 | 3.3094 | 8.6242 | 8.3224 |
| 8.4062 | 12.1646 | 8.6272 | 0.4939 |
| 8.4169 | 5.7355 | 8.713 | 3.4045 |
| 8.4217 | 10.0516 | 8.7297 | 5.5824 |
| 8.4218 | 7.0383 | 8.7715 | 8.1341 |
| 8.4562 | 2.7939 | 8.7742 | -2.6688 |
| 8.4608 | 4.9067 | 8.7802 | 11.4641 |
| 8.4679 | 8.6817 | 8.7889 | 2.2763 |
| 8.4786 | 9.7563 | 8.7937 | 6.0073 |
| 8.4969 | 8.1088 | 8.795 | 0.9942 |
| 8.5013 | 6.4651 | 8.815 | 6.5413 |
| 8.5031 | 9.9618 | 8.8183 | 2.3146 |
| 8.5074 | 4.9241 | 8.8235 | 8.517 |
| 8.5141 | 7.6897 | 8.8261 | 2.6516 |
| 8.5203 | 4.7917 | 8.8285 | 6.2826 |
| 8.5228 | 9.7442 | 8.8375 | 5.8896 |
| 8.5396 | 8.917 | 8.848 | 5.5169 |
| 8.5601 | 15.7912 | 8.848 | 8.3896 |
| 8.5704 | 1.6823 | 8.8604 | 6.337 |
| 8.5708 | 1.7126 | 8.8707 | -0.34 |
| 8.5804 | 10.2301 | 8.872 | 7.8504 |
| 8.625 | 2.2578 | 8.8782 | 8.8198 |
| 8.6314 | 8.154 | 8.8798 | 2.7891 |
| 8.6323 | 4.4448 | 8.9257 | 1.0283 |
| 8.6493 | 5.9588 | 8.9264 | 7.5278 |
| 8.6495 | 3.0437 | 8.9586 | 3.0472 |
| 8.6717 | 2.0386 | 8.9661 | 5.9497 |
| 8.6936 | 9.1496 | 8.9728 | 1.5824 |
| 8.7003 | 8.8361 | 8.9805 | 2.5315 |
| 8.7069 | 10.7855 | 8.9832 | -3.0275 |
| 8.7167 | 9.5 | 8.9909 | 7.5147 |
| 8.7184 | 3.4691 | 9.003 | 2.1646 |
| 8.728 | 6.1434 | 9.0055 | 7.6525 |
| 8.7291 | 6.49 | 9.0072 | 2.598 |
| 8.7385 | 1.88 | 9.0207 | 1.2953 |
| 8.7436 | 8.7474 | 9.0558 | 11.5284 |
| 8.7743 | 5.9596 | 9.0627 | 7.121 |
| 8.777 | 7.6203 | 9.064 | -1.168 |
| 8.7879 | 5.4948 | 9.066 | 3.0163 |
| 8.7893 | 6.6456 | 9.0669 | 5.9032 |
| 8.8224 | 4.8371 | 9.0697 | 2.9462 |
| 8.831 | 10.8199 | 9.0724 | -1.7038 |
| 8.8418 | 5.6007 | 9.0732 | 6.418 |
| 8.8523 | 2.584 | 9.0903 | 4.0365 |
| 8.8857 | 1.996 | 9.105 | 0.4777 |
| 8.9 | 1.7394 | 9.1137 | -2.1555 |
| 8.919 | 4.6977 | 9.1171 | 4.268 |
| 8.9252 | 1.3452 | 9.1179 | 3.6468 |
| 8.9322 | 5.3838 | 9.1231 | 2.1737 |
| 8.976 | 5.4924 | 9.1296 | -1.2393 |
| 8.9837 | 5.2846 | 9.1297 | -1.2768 |
| 8.9951 | 7.8086 | 9.1378 | 4.293 |
| 8.9966 | 3.8666 | 9.1625 | -1.3388 |
| 9.0309 | 7.1501 | 9.1654 | -1.2107 |
| 9.047 | 3.3219 | 9.1665 | -1.2235 |
| 9.0626 | 1.037 | 9.1685 | -1.4104 |
| 9.065 | -1.0918 | 9.1959 | 1.9864 |
| 9.0695 | 1.1976 | 9.2056 | 9.3341 |
| 9.0838 | -1.6455 | 9.2121 | -0.8495 |
| 9.0869 | -0.5745 | 9.2211 | 8.2666 |
| 9.1044 | 2.1338 | 9.2252 | 5.0758 |
| 9.1212 | 2.5859 | 9.2256 | -1.2324 |
| 9.122 | 3.0841 | 9.2359 | -5.0208 |
| 9.131 | 8.0507 | 9.2391 | -1.4144 |
| 9.1376 | 7.187 | 9.2625 | -4.3816 |
| 9.1444 | 2.7651 | 9.2665 | 3.3286 |
| 9.1511 | 0.5221 | 9.2677 | 2.2089 |
| 9.1882 | 6.1225 | 9.2756 | 1.2891 |
| 9.2324 | 4.4124 | 9.2835 | -3.2801 |
| 9.2335 | 3.4835 | 9.3085 | -3.6797 |
| 9.234 | 1.1117 | 9.3123 | -1.4521 |
| 9.2356 | -0.8843 | 9.3219 | 4.4134 |
| 9.239 | 8.1466 | 9.3253 | 4.1493 |
| 9.2484 | -2.2626 | 9.327 | 6.0308 |
| 9.258 | 2.8544 | 9.336 | 7.2215 |
| 9.263 | -2.5527 | 9.3453 | 3.4563 |
| 9.265 | 3.7487 | 9.3589 | 2.9226 |
| 9.2694 | 6.0887 | 9.361 | 5.9073 |
| 9.2886 | 5.7115 | 9.3726 | 0.0582 |
| 9.3012 | -4.188 | 9.3778 | 4.2119 |
| 9.3295 | -2.9307 | 9.379 | 2.7117 |
| 9.3444 | -5.4827 | 9.3806 | 5.6732 |
| 9.3631 | 3.9716 | 9.4097 | -3.0705 |
| 9.3743 | -2.6521 | 9.4196 | -6.171 |
| 9.3791 | 6.2501 | 9.4218 | -0.0612 |
| 9.3985 | 1.1946 | 9.4265 | -4.4403 |
| 9.4026 | -2.0467 | 9.4712 | 6.5542 |
| 9.4066 | 4.1126 | 9.4773 | -0.4983 |
| 9.4351 | 6.8541 | 9.5002 | 0.667 |
| 9.4474 | -6.6081 | 9.539 | -4.5759 |
| 9.4725 | 1.5464 | 9.5592 | -2.5198 |
| 9.4895 | 6.9594 | 9.573 | 2.9003 |
| 9.5004 | 4.5647 | 9.573 | -7.6892 |
| 9.5156 | 1.423 | 9.5782 | -3.1167 |
| 9.5306 | -1.7811 | 9.5792 | -2.8148 |
| 9.545 | -5.9224 | 9.582 | 6.2859 |
| 9.5649 | 1.9328 | 9.5881 | 0.0416 |
| 9.6003 | -3.3716 | 9.5953 | -7.9764 |
| 9.6041 | -0.261 | 9.5995 | -3.5406 |
| 9.6067 | 1.4486 | 9.6136 | 2.9887 |
| 9.6092 | 0.1877 | 9.6163 | -1.0215 |
| 9.6112 | -1.8282 | 9.6204 | -1.71 |
| 9.6261 | 2.1479 | 9.6266 | -1.1652 |
| 9.6341 | -2.5947 | 9.6294 | -1.0427 |
| 9.639 | -0.1973 | 9.6384 | -3.7098 |
| 9.6419 | -5.3452 | 9.6574 | 5.7931 |
| 9.6428 | -1.8308 | 9.6808 | -2.0879 |
| 9.6463 | -5.2101 | 9.6841 | 0.268 |
| 9.6505 | -1.124 | 9.7086 | 5.6166 |
| 9.6512 | -1.6858 | 9.7305 | -3.5858 |
| 9.6544 | -1.5909 | 9.7468 | 0.6979 |
| 9.6652 | -3.4026 | 9.7522 | -4.914 |
| 9.6874 | -4.7964 | 9.7743 | -5.5899 |
| 9.6966 | -1.8799 | 9.7768 | -4.4319 |
| 9.698 | -4.2341 | 9.7875 | -2.3684 |
| 9.7114 | -3.4314 | 9.8126 | -15.7314 |
| 9.7252 | -8.7974 | 9.8156 | -6.5871 |
| 9.729 | -3.6907 | 9.8161 | -6.1664 |
| 9.7309 | -5.6615 | 9.8307 | -6.0491 |
| 9.7657 | 1.926 | 9.8319 | -3.6094 |
| 9.7668 | 0.2847 | 9.8571 | 5.3534 |
| 9.8174 | 0.6976 | 9.8582 | -9.2926 |
| 9.8191 | -6.2084 | 9.8662 | -5.1387 |
| 9.8217 | -12.0868 | 9.8671 | -8.2902 |
| 9.8325 | -4.0754 | 9.8672 | -4.02 |
| 9.8363 | -2.3859 | 9.8947 | -6.6862 |
| 9.8428 | -4.1979 | 9.918 | -5.1329 |
| 9.8668 | 2.6898 | 9.9234 | -7.6934 |
| 9.8798 | -5.1072 | 9.929 | -0.4533 |
| 9.8832 | 3.7644 | 9.9337 | -2.8556 |
| 9.8863 | 0.6926 | 9.9343 | -6.8825 |
| 9.9052 | -1.3048 | 9.9405 | -4.1157 |
| 9.9115 | 0.9025 | 9.9442 | 2.6107 |
| 9.9127 | -3.4362 | 9.9465 | -8.653 |
| 9.9173 | -4.4931 | 9.953 | 0.2266 |
| 9.9239 | -9.3223 | 9.9534 | -1.6134 |
| 9.9416 | 0.3686 | 9.9609 | -4.2855 |
| 9.9723 | -6.8896 | 9.9661 | -1.7135 |
| 9.9821 | -19.4772 | 9.974 | -0.9742 |
| 9.9943 | 4.1316 | 9.9886 | -13.9494 |
| 9.9952 | -3.0715 | 9.9983 | -11.3288 |
| 10.0245 | -14.2104 | 10.0452 | -6.7802 |
| 10.0296 | -3.3596 | 10.0466 | 0.1298 |
| 10.071 | -6.1596 | 10.0628 | -9.65 |
| 10.0954 | -6.7452 | 10.1139 | -5.2245 |
| 10.146 | -6.8093 | 10.1172 | -6.4647 |
| 10.1603 | -7.9815 | 10.1239 | -2.3095 |
| 10.228 | -10.9246 | 10.1299 | -9.5746 |
| 10.2284 | -9.8265 | 10.13 | 1.1214 |
| 10.231 | -7.9856 | 10.177 | -12.1571 |
| 10.3293 | -19.2119 | 10.185 | -6.8074 |
| 10.3456 | -11.6712 | 10.2062 | -4.9859 |
| 10.3458 | -5.4145 | 10.2229 | -7.7702 |
| 10.3487 | -8.4491 | 10.2306 | -10.1243 |
| 10.3728 | -7.7322 | 10.2968 | -8.5786 |
| 10.3904 | -10.8341 | 10.3541 | -10.578 |
| 10.4079 | -4.5033 | 10.3573 | -10.1095 |
| 10.4146 | -15.3146 | 10.3842 | -4.9565 |
| 10.4413 | -5.0315 | 10.4281 | -4.2714 |
| 10.4654 | -12.4096 | 10.454 | -8.9586 |
| 10.5507 | 1.1833 | 10.5084 | -17.3185 |
| 10.5517 | -7.9937 | 10.5106 | 0.2908 |
| 10.5683 | -6.7017 | 10.515 | -11.5452 |
| 10.6296 | -16.8769 | 10.5398 | -7.7217 |
| 10.6397 | -12.0817 | 10.5658 | -12.385 |
| 10.6558 | -7.4206 | 10.5925 | -10.6 |
| 10.7163 | -8.479 | 10.6051 | -11.2926 |
| 10.7427 | -9.4971 | 10.6169 | -12.9282 |
| 10.7927 | -12.6762 | 10.6524 | -17.5899 |
| 10.793 | -14.4944 | 10.6737 | -17.9325 |
| 10.8493 | -11.9108 | 10.6842 | -8.3007 |
| 10.8567 | -14.9952 | 10.6972 | -7.5883 |
| 10.8607 | -16.6796 | 10.7119 | -5.1255 |
| 10.8772 | -20.1787 | 10.7497 | -10.116 |
| 10.9298 | -5.7359 | 10.7731 | -13.633 |
| 10.9765 | -6.9497 | 10.7933 | -11.5352 |
| 10.9766 | -7.8564 | 10.8471 | -16.5083 |
| 11.066 | -14.0312 | 10.8764 | -10.9172 |
| 11.1173 | -10.8501 | 10.896 | -15.5344 |
| 11.1191 | -6.6365 | 10.9317 | -12.5267 |
| 11.1363 | -9.0775 | 10.9505 | -14.5688 |
| 11.1942 | -16.8244 | 10.9516 | -7.1813 |
| 11.1995 | -9.7033 | 10.972 | -3.6936 |
| 11.2268 | -7.5306 | 10.9931 | -14.8245 |
| 11.2609 | -5.941 | 11.0235 | -15.115 |
| 11.3128 | -15.1957 | 11.0538 | -17.275 |
| 11.3377 | -12.4046 | 11.0682 | -6.58 |
| 11.3458 | -4.4336 | 11.0822 | -12.2432 |
| 11.3576 | -9.4285 | 11.0892 | -15.7721 |
| 11.3718 | -9.662 | 11.1157 | -6.1154 |
| 11.3846 | -10.2913 | 11.1408 | -15.2471 |
| 11.4973 | -15.582 | 11.1763 | -22.9959 |
| 11.5106 | -17.3017 | 11.1849 | -10.0835 |
| 11.5127 | -12.2698 | 11.1999 | -13.4998 |
| 11.5194 | -7.1719 | 11.2443 | -16.7748 |
| 11.5204 | -14.406 | 11.2524 | -14.6838 |
| 11.5213 | -7.2914 | 11.2659 | -8.2143 |
| 11.567 | -6.0851 | 11.2814 | -8.0344 |
| 11.5984 | -4.7378 | 11.2867 | -12.8687 |
| 11.6395 | -7.2097 | 11.3497 | -10.7549 |
| 11.6644 | -0.9305 | 11.3662 | -6.1222 |
| 11.7488 | -10.5738 | 11.416 | -6.3145 |
| 11.7566 | -9.2609 | 11.4358 | -9.2805 |
| 11.7969 | -12.5496 | 11.4747 | -9.7519 |
| 11.826 | -11.4902 | 11.5233 | -14.4589 |
| 11.8464 | -2.2452 | 11.5423 | -9.9688 |
| 11.869 | -3.3137 | 11.5425 | -10.1223 |
| 11.8821 | -3.3558 | 11.5548 | -13.2984 |
| 11.9229 | -21.7597 | 11.5568 | -10.553 |
| 11.929 | -17.0355 | 11.597 | -7.2452 |
| 11.9325 | -2.7738 | 11.6011 | 2.235 |
| 11.9764 | -11.1487 | 11.6158 | -13.9657 |
| 12.0093 | -1.9615 | 11.6262 | -8.4663 |
| 12.0458 | -2.6882 | 11.6295 | -5.8724 |
| 12.1159 | -9.2913 | 11.6495 | -8.1068 |
| 12.1481 | -12.8278 | 11.6497 | -13.1461 |
| 12.169 | -9.6582 | 11.6545 | -4.1165 |
| 12.194 | -8.1863 | 11.6695 | 0.5724 |
| 12.2075 | -3.4853 | 11.6759 | -9.598 |
| 12.2172 | -7.6505 | 11.6825 | -14.3509 |
| 12.2379 | -0.2668 | 11.6888 | -5.6703 |
| 12.2454 | -2.0254 | 11.696 | -0.9323 |
| 12.2663 | -8.7207 | 11.7343 | -12.9664 |
| 12.2693 | 0.9853 | 11.7445 | -6.2342 |
| 12.316 | -2.847 | 11.7484 | -10.9744 |
| 12.3165 | 6.9623 | 11.8136 | -2.158 |
| 12.3223 | -2.0023 | 11.8201 | -5.4141 |
| 12.3396 | -4.2201 | 11.8377 | -2.5674 |
| 12.3562 | -8.5319 | 11.8379 | -19.1196 |
| 12.3596 | 2.4415 | 11.8684 | -17.8599 |
| 12.3928 | 0.1396 | 11.8979 | -3.0552 |
| 12.4148 | -1.5533 | 11.9377 | -0.3278 |
| 12.4287 | -3.5919 | 11.9494 | -11.3786 |
| 12.4485 | -7.1546 | 11.9811 | -9.5757 |
| 12.469 | -2.9123 | 12.0054 | -8.4579 |
| 12.4852 | -0.0162 | 12.023 | -7.6247 |
| 12.4914 | -9.3923 | 12.0498 | -12.4902 |
| 12.5201 | -7.0384 | 12.1292 | -6.9755 |
| 12.6232 | -6.4087 | 12.1311 | -0.846 |
| 12.6632 | 2.2145 | 12.1726 | -4.9134 |
| 12.6655 | -7.3451 | 12.1791 | -0.7073 |
| 12.7091 | 3.9079 | 12.2073 | 0.796 |
| 12.7902 | 4.2347 | 12.2158 | -11.1819 |
| 12.7971 | -0.8859 | 12.2835 | 0.1393 |
| 12.8114 | -2.3581 | 12.3012 | -8.2348 |
| 12.8282 | 3.3131 | 12.3224 | 9.7223 |
| 12.8423 | -5.7927 | 12.3354 | -8.9196 |
| 12.9268 | 0.5046 | 12.413 | -4.3179 |
| 12.9325 | -0.6999 | 12.49 | 0.457 |
| 12.9464 | 5.0708 | 12.4928 | -5.982 |
| 12.9471 | 1.4045 | 12.5014 | -0.6035 |
| 12.9864 | -1.7517 | 12.51 | 5.2204 |
| 13.0211 | -1.511 | 12.5196 | 0.4051 |
| 13.0654 | 2.5603 | 12.5235 | 0.4421 |
| 13.0832 | 10.6454 | 12.5564 | 1.2855 |
| 13.0848 | -1.0868 | 12.6414 | -7.4156 |
| 13.1103 | -0.2016 | 12.6692 | -2.0177 |
| 13.1314 | -0.0897 | 12.6823 | 6.1002 |
| 13.2168 | 20.7191 | 12.6866 | 5.8634 |
| 13.268 | 5.6395 | 12.6914 | -1.2471 |
| 13.2793 | -0.4515 | 12.7219 | 0.2351 |
| 13.3086 | 5.9978 | 12.7465 | 2.2709 |
| 13.3896 | 13.2022 | 12.7869 | -2.1265 |
| 13.4058 | 13.6972 | 12.796 | 1.2727 |
| 13.4127 | 0.6847 | 12.8677 | 3.0616 |
| 13.4489 | 8.0375 | 12.8714 | 9.666 |
| 13.4509 | 13.4411 | 12.8747 | 13.4139 |
| 13.4635 | 13.7493 | 12.9848 | 3.856 |
| 13.4898 | 5.6467 | 12.9983 | -3.1166 |
| 13.5149 | 12.5499 | 13.0086 | 7.7379 |
| 13.5258 | 16.9917 | 13.0324 | 10.6873 |
| 13.5346 | 12.7234 | 13.0405 | 7.1924 |
| 13.5445 | 11.1038 | 13.0775 | 11.666 |
| 13.5573 | 5.7345 | 13.1487 | 2.4325 |
| 13.593 | 20.3043 | 13.1845 | 12.1979 |
| 13.6105 | 19.3217 | 13.2717 | 4.0727 |
| 13.6147 | 14.0703 | 13.2922 | 15.3507 |
| 13.6674 | 8.5214 | 13.3771 | 12.9502 |
| 13.6912 | 18.4127 | 13.4025 | 4.7326 |
| 13.7022 | -0.5819 | 13.4133 | 2.9155 |
| 13.7659 | 24.5598 | 13.4293 | 6.0728 |
| 13.7724 | 13.2582 | 13.4965 | -5.7818 |
| 13.7775 | 12.2627 | 13.5174 | 4.6586 |
| 13.8191 | 22.6168 | 13.5233 | 8.2816 |
| 13.8239 | 18.5403 | 13.5261 | 7.005 |
| 13.829 | 20.7691 | 13.5424 | 5.6354 |
| 13.8374 | 14.336 | 13.573 | 9.8052 |
| 13.8605 | 23.8522 | 13.598 | 19.3827 |
| 13.8711 | 7.4096 | 13.5983 | 2.7578 |
| 13.8859 | 18.5749 | 13.604 | 21.1599 |
| 13.9125 | 13.6523 | 13.6063 | 14.274 |
| 13.9174 | 5.2748 | 13.6171 | 10.7108 |
| 13.9368 | 13.3077 | 13.6238 | 2.8666 |
| 13.9425 | 16.4644 | 13.6517 | 8.6677 |
| 13.9588 | 3.0668 | 13.6528 | 8.4997 |
| 13.9621 | 21.5767 | 13.6575 | 8.0476 |
| 14.0116 | 3.8208 | 13.6931 | 14.4198 |
| 14.0143 | 9.4739 | 13.6944 | 9.2317 |
| 14.0171 | 17.5958 | 13.7101 | 16.9091 |
| 14.0501 | 8.5375 | 13.7412 | 9.2841 |
| 14.0764 | 10.3565 | 13.7558 | 16.0684 |
| 14.0771 | 9.0267 | 13.7608 | 13.9252 |
| 14.1111 | 13.1673 | 13.7881 | 0.4123 |
| 14.1348 | 25.3931 | 13.8568 | 24.4517 |
| 14.1496 | 17.4673 | 13.8568 | 19.0698 |
| 14.1633 | 10.3884 | 13.8615 | -3.643 |
| 14.1771 | 15.5528 | 13.9014 | 10.1689 |
| 14.2122 | 7.4148 | 13.9958 | 5.5919 |
| 14.2162 | 19.0523 | 14.0456 | 23.8859 |
| 14.2514 | 16.5376 | 14.0891 | 25.2947 |
| 14.2655 | 8.8179 | 14.1301 | 11.742 |
| 14.2791 | 10.9063 | 14.1476 | 7.4142 |
| 14.2807 | 24.6792 | 14.1489 | 15.3385 |
| 14.2993 | 9.7423 | 14.1765 | 7.3345 |
| 14.301 | 15.6201 | 14.1864 | 27.8179 |
| 14.3079 | 17.4666 | 14.2019 | 11.0694 |
| 14.3875 | 19.736 | 14.2027 | 16.5776 |
| 14.4626 | 14.1009 | 14.2339 | 10.461 |
| 14.4762 | 9.743 | 14.2515 | 16.7286 |
| 14.5309 | 23.5065 | 14.3468 | 14.9915 |
| 14.5467 | 25.0347 | 14.3535 | 4.9105 |
| 14.5497 | 19.3906 | 14.3718 | 14.0702 |
| 14.5625 | 21.9359 | 14.3872 | 21.838 |
| 14.5679 | 13.7839 | 14.394 | 13.2607 |
| 14.5736 | 19.4734 | 14.4155 | 26.2367 |
| 14.5775 | 16.406 | 14.4626 | 19.52 |
| 14.5921 | 12.14 | 14.5313 | 19.2228 |
| 14.5954 | 12.9417 | 14.5347 | 15.516 |
| 14.597 | 2.7478 | 14.5655 | 19.5352 |
| 14.682 | 5.5693 | 14.6325 | 19.0835 |
| 14.7391 | 12.8229 | 14.6506 | 18.447 |
| 14.7423 | 7.7147 | 14.7237 | 10.3544 |
| 14.7635 | 3.6342 | 14.7824 | 12.1922 |
| 14.7847 | 9.283 | 14.802 | 23.6772 |
| 14.8158 | 14.0422 | 14.821 | 19.2956 |
| 14.8167 | 13.293 | 14.8326 | 1.5942 |
| 14.8471 | 12.4243 | 14.8557 | 5.8801 |
| 14.9066 | 3.7638 | 14.8626 | 18.5306 |
| 14.956 | 18.0934 | 14.8655 | 10.8198 |
| 14.9639 | 20.0575 | 14.9334 | 13.3506 |
| 14.9936 | 7.0906 | 14.9587 | 11.9204 |
|  |  | 14.9735 | 0.0025 |
|  |  | 14.9984 | 0.002 |
|  |  | 15.0014 | 0.0261 |
|  |  | 15.0016 | -0.0227 |
|  |  | 15.0133 | 0.009 |
|  |  | 15.0271 | -0.0464 |
|  |  | 15.0853 | -0.0119 |
|  |  | 15.0884 | -0.0052 |
|  |  | 15.0947 | 0.079 |
|  |  | 15.0995 | -0.0474 |
|  |  | 15.1051 | -0.0635 |
|  |  | 15.1107 | 0.0274 |
|  |  | 15.1125 | -0.0433 |
|  |  | 15.1243 | 0.0365 |
|  |  | 15.1271 | 0.1662 |
|  |  | 15.131 | 0.2347 |
|  |  | 15.1429 | 0.0117 |
|  |  | 15.151 | -0.1578 |
|  |  | 15.1539 | 0.0575 |
|  |  | 15.1552 | -0.0389 |
|  |  | 15.1612 | -0.0248 |
|  |  | 15.1693 | -0.0825 |
|  |  | 15.2107 | -0.1013 |
|  |  | 15.2566 | 0.0905 |
|  |  | 15.2991 | 0.2042 |
|  |  | 15.3059 | 0.1036 |
|  |  | 15.3201 | -0.1366 |
|  |  | 15.3243 | 0.1492 |
|  |  | 15.3343 | 0.3999 |
|  |  | 15.3446 | 0.4941 |
|  |  | 15.363 | -0.0348 |
|  |  | 15.3632 | 0.3179 |
|  |  | 15.3636 | -0.0553 |
|  |  | 15.3828 | 0.0336 |
|  |  | 15.3864 | 0.1423 |
|  |  | 15.3884 | 0.1701 |
|  |  | 15.4049 | 0.2825 |
|  |  | 15.4174 | 0.1156 |
|  |  | 15.4594 | 0.5904 |
|  |  | 15.4693 | 0.1541 |
|  |  | 15.4799 | 0.1985 |
|  |  | 15.4808 | 0.2314 |
|  |  | 15.4898 | 0.0563 |
|  |  | 15.498 | 0.5095 |
|  |  | 15.4982 | 0.2415 |
|  |  | 15.5053 | 0.2359 |
|  |  | 15.524 | -0.1003 |
|  |  | 15.5538 | 0.0793 |
|  |  | 15.5629 | -0.1588 |
|  |  | 15.5665 | 0.3195 |
|  |  | 15.5778 | 0.6165 |
|  |  | 15.5799 | 0.6839 |
|  |  | 15.5846 | 0.2516 |
|  |  | 15.5847 | 0.5489 |
|  |  | 15.5928 | 1.1846 |
|  |  | 15.5939 | -0.131 |
|  |  | 15.5939 | 0.6684 |
|  |  | 15.5955 | 0.4047 |
|  |  | 15.6007 | 0.1746 |
|  |  | 15.6009 | 0.6414 |
|  |  | 15.6079 | 0.2454 |
|  |  | 15.6168 | 0.9479 |
|  |  | 15.6468 | -0.0974 |
|  |  | 15.6539 | 0.6761 |
|  |  | 15.6645 | 0.1758 |
|  |  | 15.6658 | 0.4267 |
|  |  | 15.6927 | 0.8836 |
|  |  | 15.6965 | 0.6091 |
|  |  | 15.7025 | 0.5204 |
|  |  | 15.7028 | 0.5754 |
|  |  | 15.7045 | 1.4404 |
|  |  | 15.7324 | 1.0058 |
|  |  | 15.7346 | 0.1979 |
|  |  | 15.7421 | 0.5255 |
|  |  | 15.7426 | 0.7626 |
|  |  | 15.7466 | 0.5215 |
|  |  | 15.7483 | 0.6534 |
|  |  | 15.7973 | 0.6015 |
|  |  | 15.8056 | 0.5903 |
|  |  | 15.8101 | 0.3716 |
|  |  | 15.8106 | 0.5055 |
|  |  | 15.8136 | 0.347 |
|  |  | 15.8275 | 0.6768 |
|  |  | 15.8738 | 1.0848 |
|  |  | 15.8903 | 0.2662 |
|  |  | 15.9084 | 1.136 |
|  |  | 15.9142 | 1.4863 |
|  |  | 15.9189 | 0.1374 |
|  |  | 15.9225 | 0.7737 |
|  |  | 15.9343 | 1.164 |
|  |  | 15.9347 | 1.348 |
|  |  | 15.942 | 0.9005 |
|  |  | 15.9662 | 0.3774 |
|  |  | 15.9756 | 0.9675 |
|  |  | 15.9886 | 1.4796 |
|  |  | 16.0003 | 0.9306 |
|  |  | 16.0071 | 0.487 |
|  |  | 16.0098 | 1.2144 |
|  |  | 16.0371 | 1.3389 |
|  |  | 16.0624 | 0.2124 |
|  |  | 16.0783 | 1.0014 |
|  |  | 16.0998 | 0.8039 |
|  |  | 16.1053 | 0.8541 |
|  |  | 16.1162 | 1.1619 |
|  |  | 16.1222 | 0.2907 |
|  |  | 16.1233 | 1.3907 |
|  |  | 16.1303 | 1.5641 |
|  |  | 16.1357 | 1.0473 |
|  |  | 16.1375 | 1.1267 |
|  |  | 16.1521 | 1.574 |
|  |  | 16.1609 | 0.8878 |
|  |  | 16.1728 | 0.6908 |
|  |  | 16.198 | 0.9697 |
|  |  | 16.2078 | 1.0187 |
|  |  | 16.2147 | 0.5606 |
|  |  | 16.2232 | 1.6495 |
|  |  | 16.2313 | 1.3829 |
|  |  | 16.245 | 0.4902 |
|  |  | 16.2482 | 0.9134 |
|  |  | 16.2613 | 1.3919 |
|  |  | 16.2721 | 1.5566 |
|  |  | 16.2747 | 1.5401 |
|  |  | 16.2777 | 1.8269 |
|  |  | 16.2869 | 1.6251 |
|  |  | 16.2933 | 1.8763 |
|  |  | 16.3007 | 1.1165 |
|  |  | 16.308 | 0.9622 |
|  |  | 16.3108 | 1.0209 |
|  |  | 16.3111 | 0.6926 |
|  |  | 16.3376 | 1.7919 |
|  |  | 16.3608 | 1.6923 |
|  |  | 16.3702 | 1.3872 |
|  |  | 16.3778 | 1.9558 |
|  |  | 16.3882 | 1.4254 |
|  |  | 16.399 | 0.6378 |
|  |  | 16.4095 | 0.6674 |
|  |  | 16.4162 | 1.8108 |
|  |  | 16.4183 | 1.6491 |
|  |  | 16.4213 | 1.2768 |
|  |  | 16.4506 | 1.3232 |
|  |  | 16.464 | 1.9307 |
|  |  | 16.4654 | 1.5773 |
|  |  | 16.469 | 0.8779 |
|  |  | 16.4952 | 1.6491 |
|  |  | 16.5005 | 2.4296 |
|  |  | 16.505 | 2.0398 |
|  |  | 16.508 | 0.3865 |
|  |  | 16.5117 | 1.4757 |
|  |  | 16.5125 | 2.412 |
|  |  | 16.5283 | 1.9533 |
|  |  | 16.5308 | 0.1703 |
|  |  | 16.5331 | 2.1045 |
|  |  | 16.5336 | 0.6737 |
|  |  | 16.537 | 1.8951 |
|  |  | 16.5383 | 1.8753 |
|  |  | 16.5474 | 0.8298 |
|  |  | 16.5528 | 2.8712 |
|  |  | 16.5551 | 0.7957 |
|  |  | 16.5668 | 1.8559 |
|  |  | 16.5782 | 1.189 |
|  |  | 16.6063 | 1.8569 |
|  |  | 16.607 | 1.6306 |
|  |  | 16.6394 | 3.3665 |
|  |  | 16.6491 | 2.2207 |
|  |  | 16.6634 | 1.3093 |
|  |  | 16.6656 | 1.5935 |
|  |  | 16.6741 | 1.1852 |
|  |  | 16.6938 | 1.2876 |
|  |  | 16.6996 | 1.7829 |
|  |  | 16.7036 | 1.8598 |
|  |  | 16.7078 | 2.4844 |
|  |  | 16.7194 | 0.6859 |
|  |  | 16.7257 | 1.2579 |
|  |  | 16.7274 | 1.5568 |
|  |  | 16.7419 | 0.7176 |
|  |  | 16.7633 | 1.9929 |
|  |  | 16.7904 | 1.1107 |
|  |  | 16.7923 | 2.0119 |
|  |  | 16.8107 | 2.4376 |
|  |  | 16.8118 | 2.5701 |
|  |  | 16.8151 | 0.8219 |
|  |  | 16.8358 | 1.3149 |
|  |  | 16.8363 | 0.9922 |
|  |  | 16.8366 | 1.5021 |
|  |  | 16.8506 | 1.6282 |
|  |  | 16.8677 | 1.9319 |
|  |  | 16.8776 | 1.8438 |
|  |  | 16.8844 | 0.1577 |
|  |  | 16.8882 | 2.1159 |
|  |  | 16.8968 | 0.2094 |
|  |  | 16.9064 | 1.8725 |
|  |  | 16.9304 | 1.772 |
|  |  | 16.9376 | 1.6574 |
|  |  | 16.9484 | 2.5266 |
|  |  | 16.9487 | 0.3261 |
|  |  | 16.9511 | 0.5849 |
|  |  | 16.9511 | 3.109 |
|  |  | 16.9533 | 3.3691 |
|  |  | 16.958 | 1.3455 |
|  |  | 16.9591 | 3.2437 |
|  |  | 16.9614 | 2.1975 |
|  |  | 16.978 | 1.5014 |
|  |  | 16.9992 | 2.4594 |
|  |  | 16.9998 | 2.291 |
|  |  | 17.0183 | 1.014 |
|  |  | 17.0197 | 1.1425 |
|  |  | 17.0206 | 2.8421 |
|  |  | 17.0254 | 2.4545 |
|  |  | 17.0293 | 1.9668 |
|  |  | 17.0302 | 0.6021 |
|  |  | 17.0316 | 1.4868 |
|  |  | 17.0384 | 1.3333 |
|  |  | 17.0421 | 0.6449 |
|  |  | 17.0428 | 2.01 |
|  |  | 17.0597 | 1.1299 |
|  |  | 17.0711 | -0.3577 |
|  |  | 17.072 | 2.2778 |
|  |  | 17.0753 | 1.7388 |
|  |  | 17.0823 | 2.2022 |
|  |  | 17.0841 | 1.6394 |
|  |  | 17.0854 | 1.9029 |
|  |  | 17.0896 | 1.6713 |
|  |  | 17.0901 | 2.9094 |
|  |  | 17.1113 | -0.0233 |
|  |  | 17.1127 | 2.3681 |
|  |  | 17.1161 | 1.887 |
|  |  | 17.1382 | 0.8617 |
|  |  | 17.1397 | 1.7477 |
|  |  | 17.144 | 1.4932 |
|  |  | 17.1508 | 1.9713 |
|  |  | 17.1551 | 3.6817 |
|  |  | 17.161 | 1.8473 |
|  |  | 17.1613 | 1.0353 |
|  |  | 17.1677 | 2.1589 |
|  |  | 17.1819 | 0.8752 |
|  |  | 17.1873 | 3.4427 |
|  |  | 17.1959 | 1.3078 |
|  |  | 17.2135 | 2.8398 |
|  |  | 17.2166 | 2.277 |
|  |  | 17.2311 | 0.2059 |
|  |  | 17.2365 | 1.4335 |
|  |  | 17.2608 | -1.0355 |
|  |  | 17.2627 | 0.345 |
|  |  | 17.2959 | -0.6873 |
|  |  | 17.2978 | -0.2338 |
|  |  | 17.3043 | 0.7214 |
|  |  | 17.305 | 2.1055 |
|  |  | 17.3214 | 1.8261 |
|  |  | 17.3347 | 2.0221 |
|  |  | 17.3754 | 0.9282 |
|  |  | 17.4058 | 1.408 |
|  |  | 17.4499 | 2.2059 |
|  |  | 17.4519 | 2.6873 |
|  |  | 17.486 | 0.4547 |
|  |  | 17.4875 | 2.3165 |
|  |  | 17.4918 | 2.7289 |
|  |  | 17.514 | 0.3618 |
|  |  | 17.5197 | 1.1335 |
|  |  | 17.5266 | 2.9219 |
|  |  | 17.529 | 0.8779 |
|  |  | 17.5382 | 0.8287 |
|  |  | 17.5514 | 1.1142 |
|  |  | 17.5537 | 0.1435 |
|  |  | 17.5686 | 2.198 |
|  |  | 17.5756 | 0.0474 |
|  |  | 17.5941 | 1.9543 |
|  |  | 17.6002 | 0.7905 |
|  |  | 17.6348 | -0.2067 |
|  |  | 17.6407 | 3.6514 |
|  |  | 17.6419 | -0.6918 |
|  |  | 17.6428 | 1.4471 |
|  |  | 17.6785 | 0.5381 |
|  |  | 17.6882 | 1.3383 |
|  |  | 17.6939 | 1.7736 |
|  |  | 17.7206 | 2.7863 |
|  |  | 17.7342 | 1.9551 |
|  |  | 17.7345 | 2.2776 |
|  |  | 17.7357 | 0.9865 |
|  |  | 17.7361 | 1.236 |
|  |  | 17.7407 | 3.2396 |
|  |  | 17.7415 | 1.3143 |
|  |  | 17.7448 | 2.6156 |
|  |  | 17.7648 | 1.8566 |
|  |  | 17.7779 | 1.3471 |
|  |  | 17.7904 | 0.2716 |
|  |  | 17.7947 | 0.4673 |
|  |  | 17.7968 | 1.7706 |
|  |  | 17.8037 | 1.5005 |
|  |  | 17.8045 | 0.3075 |
|  |  | 17.8205 | 2.9966 |
|  |  | 17.838 | -0.9359 |
|  |  | 17.8385 | 0.6029 |
|  |  | 17.8554 | 0.3512 |
|  |  | 17.8622 | 3.2982 |
|  |  | 17.8648 | 1.3727 |
|  |  | 17.8738 | 1.4978 |
|  |  | 17.8776 | -0.0856 |
|  |  | 17.8962 | 0.7893 |
|  |  | 17.898 | 1.3522 |
|  |  | 17.905 | 0.5629 |
|  |  | 17.9082 | -1.378 |
|  |  | 17.9303 | 0.0827 |
|  |  | 17.934 | 0.1659 |
|  |  | 17.9538 | -0.8783 |
|  |  | 17.962 | 0.9411 |
|  |  | 17.9626 | 2.0805 |
|  |  | 17.968 | -0.8049 |
|  |  | 17.9728 | 0.391 |
|  |  | 17.9802 | 0.943 |
|  |  | 17.9867 | 0.0381 |
|  |  | 18.0173 | -0.2705 |
|  |  | 18.0201 | 2.6995 |
|  |  | 18.0304 | -1.3826 |
|  |  | 18.0356 | 1.4174 |
|  |  | 18.0404 | -0.75 |
|  |  | 18.0439 | -0.7386 |
|  |  | 18.044 | -1.4995 |
|  |  | 18.045 | -0.5001 |
|  |  | 18.0546 | 0.463 |
|  |  | 18.0698 | 0.9426 |
|  |  | 18.0718 | -0.2029 |
|  |  | 18.0798 | 0.4634 |
|  |  | 18.0839 | -1.1836 |
|  |  | 18.0855 | -0.6765 |
|  |  | 18.0868 | 1.2986 |
|  |  | 18.0991 | -0.6426 |
|  |  | 18.1025 | 0.0274 |
|  |  | 18.1314 | -1.9664 |
|  |  | 18.1724 | 0.4343 |
|  |  | 18.177 | 1.5741 |
|  |  | 18.223 | -0.525 |
|  |  | 18.2272 | -0.4383 |
|  |  | 18.2361 | 0.1432 |
|  |  | 18.2465 | -1.0605 |
|  |  | 18.2558 | -1.3466 |
|  |  | 18.261 | 0.2083 |
|  |  | 18.2648 | 0.6642 |
|  |  | 18.2677 | -1.8483 |
|  |  | 18.2686 | -0.8216 |
|  |  | 18.2774 | 1.0247 |
|  |  | 18.2825 | -1.0438 |
|  |  | 18.2907 | -2.419 |
|  |  | 18.2978 | 2.8416 |
|  |  | 18.2997 | 2.1599 |
|  |  | 18.3007 | -1.2733 |
|  |  | 18.3203 | -0.9914 |
|  |  | 18.3215 | -0.5003 |
|  |  | 18.3222 | 0.0734 |
|  |  | 18.3252 | -2.7106 |
|  |  | 18.3294 | 0.4308 |
|  |  | 18.3424 | 1.5435 |
|  |  | 18.3536 | -3.1568 |
|  |  | 18.358 | -3.1958 |
|  |  | 18.3927 | 0.7918 |
|  |  | 18.3965 | -0.8333 |
|  |  | 18.4085 | -4.6579 |
|  |  | 18.4098 | -1.4571 |
|  |  | 18.4274 | -1.0246 |
|  |  | 18.4329 | -0.6278 |
|  |  | 18.4657 | -1.6362 |
|  |  | 18.4665 | -2.9792 |
|  |  | 18.4724 | 0.1971 |
|  |  | 18.487 | -0.8581 |
|  |  | 18.5043 | -3.9438 |
|  |  | 18.5154 | -3.4912 |
|  |  | 18.5228 | 3.7084 |
|  |  | 18.5644 | 1.0986 |
|  |  | 18.5681 | -2.8512 |
|  |  | 18.5732 | -2.5941 |
|  |  | 18.5738 | -0.3299 |
|  |  | 18.5858 | -1.4338 |
|  |  | 18.6014 | -2.3689 |
|  |  | 18.6035 | -3.3914 |
|  |  | 18.6063 | -5.6204 |
|  |  | 18.6207 | -1.3256 |
|  |  | 18.6239 | -1.1851 |
|  |  | 18.6251 | -3.5966 |
|  |  | 18.6286 | -1.9446 |
|  |  | 18.6338 | -1.5325 |
|  |  | 18.6364 | -2.9507 |
|  |  | 18.6399 | -3.7134 |
|  |  | 18.6418 | -0.1963 |
|  |  | 18.6525 | 2.2823 |
|  |  | 18.6636 | -1.2888 |
|  |  | 18.6655 | -2.8601 |
|  |  | 18.6755 | 0.5062 |
|  |  | 18.6857 | -1.1444 |
|  |  | 18.6947 | -2.8442 |
|  |  | 18.6985 | 0.3481 |
|  |  | 18.7304 | -4.1648 |
|  |  | 18.7364 | -2.6229 |
|  |  | 18.7429 | -0.7429 |
|  |  | 18.7568 | -0.7077 |
|  |  | 18.7597 | -6.0504 |
|  |  | 18.7842 | -3.1969 |
|  |  | 18.829 | -2.5113 |
|  |  | 18.8365 | -1.7117 |
|  |  | 18.839 | -4.7338 |
|  |  | 18.84 | -3.6101 |
|  |  | 18.858 | -6.455 |
|  |  | 18.8791 | -4.2352 |
|  |  | 18.8798 | -4.9308 |
|  |  | 18.8846 | -5.8494 |
|  |  | 18.8958 | -3.3253 |
|  |  | 18.9032 | -3.6521 |
|  |  | 18.9074 | -3.235 |
|  |  | 18.9087 | -2.5245 |
|  |  | 18.9094 | -2.9442 |
|  |  | 18.9095 | -3.6942 |
|  |  | 18.914 | -1.7628 |
|  |  | 18.9179 | -4.5534 |
|  |  | 18.9276 | -3.6418 |
|  |  | 18.9597 | -1.4965 |
|  |  | 18.975 | -4.2003 |
|  |  | 18.9758 | -6.2429 |
|  |  | 18.9845 | -0.9441 |
|  |  | 18.9966 | -3.088 |
|  |  | 18.9977 | -4.4305 |
|  |  | 19.032 | -3.6844 |
|  |  | 19.0453 | -0.9098 |
|  |  | 19.046 | -1.9935 |
|  |  | 19.0497 | -4.6581 |
|  |  | 19.0528 | -1.4428 |
|  |  | 19.0549 | -2.7529 |
|  |  | 19.0578 | -2.5955 |
|  |  | 19.0684 | -1.9417 |
|  |  | 19.0722 | -4.6336 |
|  |  | 19.0822 | -2.3236 |
|  |  | 19.0828 | -1.6633 |
|  |  | 19.0961 | -4.4783 |
|  |  | 19.1079 | -4.592 |
|  |  | 19.1108 | -2.238 |
|  |  | 19.1194 | -2.3613 |
|  |  | 19.1211 | -3.2644 |
|  |  | 19.1316 | -4.3929 |
|  |  | 19.1548 | -1.4266 |
|  |  | 19.1767 | -5.2801 |
|  |  | 19.1772 | -3.1869 |
|  |  | 19.197 | -4.8872 |
|  |  | 19.2168 | -7.7165 |
|  |  | 19.221 | 0.3676 |
|  |  | 19.2285 | -3.034 |
|  |  | 19.2352 | -2.0171 |
|  |  | 19.2556 | -5.3134 |
|  |  | 19.2568 | -4.594 |
|  |  | 19.2631 | -2.2358 |
|  |  | 19.2846 | -4.6176 |
|  |  | 19.288 | -2.6855 |
|  |  | 19.297 | -1.8382 |
|  |  | 19.3013 | -3.33 |
|  |  | 19.3083 | -4.159 |
|  |  | 19.3392 | -3.4471 |
|  |  | 19.3562 | -5.8764 |
|  |  | 19.3623 | -6.1719 |
|  |  | 19.3668 | -2.9777 |
|  |  | 19.369 | -5.4022 |
|  |  | 19.3708 | -5.5791 |
|  |  | 19.3748 | -2.9331 |
|  |  | 19.379 | -4.0888 |
|  |  | 19.3886 | -4.0879 |
|  |  | 19.3971 | -3.2589 |
|  |  | 19.4104 | -4.8061 |
|  |  | 19.4268 | -5.5887 |
|  |  | 19.4313 | -2.4367 |
|  |  | 19.4485 | -4.2048 |
|  |  | 19.4494 | -6.773 |
|  |  | 19.4515 | -3.2744 |
|  |  | 19.4589 | -0.7099 |
|  |  | 19.4615 | -1.9771 |
|  |  | 19.4662 | -3.0633 |
|  |  | 19.4952 | -3.7231 |
|  |  | 19.5263 | -3.3861 |
|  |  | 19.5778 | -4.7031 |
|  |  | 19.5806 | -3.3069 |
|  |  | 19.594 | -6.2237 |
|  |  | 19.6041 | -6.8997 |
|  |  | 19.6158 | -2.6205 |
|  |  | 19.6788 | -5.1417 |
|  |  | 19.7203 | -5.8709 |
|  |  | 19.7529 | -6.4643 |
|  |  | 19.7553 | -7.5439 |
|  |  | 19.762 | -6.7316 |
|  |  | 19.7862 | -4.4155 |
|  |  | 19.8181 | -6.0845 |
|  |  | 19.8238 | -1.7411 |
|  |  | 19.8249 | -4.6911 |
|  |  | 19.8311 | -5.2067 |
|  |  | 19.836 | -7.0729 |
|  |  | 19.8368 | -4.7063 |
|  |  | 19.8454 | -6.8442 |
|  |  | 19.8492 | -4.8966 |
|  |  | 19.8601 | -0.6115 |
|  |  | 19.8782 | -5.1811 |
|  |  | 19.8803 | -4.5817 |
|  |  | 19.8816 | -4.9482 |
|  |  | 19.907 | -5.282 |
|  |  | 19.9174 | -4.6449 |
|  |  | 19.9285 | -7.2013 |
|  |  | 19.9356 | -8.2436 |
|  |  | 19.955 | -9.3514 |
|  |  | 19.9588 | -6.2086 |
|  |  | 19.9803 | -4.6291 |
|  |  | 19.9919 | -3.2836 |
|  |  | 19.9935 | -11.3964 |
|  |  | 20.0116 | 0.1864 |
|  |  | 20.0122 | 3.3788 |
|  |  | 20.057 | 0.4551 |
|  |  | 20.07 | 0.025 |
|  |  | 20.0851 | 3.0341 |
|  |  | 20.0903 | -7.2641 |
|  |  | 20.0987 | -8.6154 |
|  |  | 20.1054 | 0.6028 |
|  |  | 20.1175 | -7.7819 |
|  |  | 20.1205 | 1.0308 |
|  |  | 20.1304 | -1.4958 |
|  |  | 20.1323 | -9.4612 |
|  |  | 20.144 | -0.1311 |
|  |  | 20.1477 | 4.6617 |
|  |  | 20.1487 | -0.8099 |
|  |  | 20.1585 | 3.2688 |
|  |  | 20.1588 | -3.7671 |
|  |  | 20.159 | -3.1879 |
|  |  | 20.1592 | -3.7897 |
|  |  | 20.1601 | -5.488 |
|  |  | 20.163 | 2.2898 |
|  |  | 20.1862 | 4.1691 |
|  |  | 20.209 | -11.5106 |
|  |  | 20.2184 | -1.6726 |
|  |  | 20.234 | 1.3417 |
|  |  | 20.2364 | 0.9289 |
|  |  | 20.237 | -11.7739 |
|  |  | 20.2397 | -0.9121 |
|  |  | 20.2689 | 0.2677 |
|  |  | 20.2728 | 3.7685 |
|  |  | 20.2742 | -2.2952 |
|  |  | 20.2761 | -13.4535 |
|  |  | 20.2937 | -10.5133 |
|  |  | 20.3237 | 0.0947 |
|  |  | 20.3262 | -4.5573 |
|  |  | 20.3405 | 3.9849 |
|  |  | 20.3423 | -5.9566 |
|  |  | 20.3448 | 1.9898 |
|  |  | 20.348 | -0.7414 |
|  |  | 20.3672 | -2.2067 |
|  |  | 20.3678 | -14.6916 |
|  |  | 20.3891 | -0.0901 |
|  |  | 20.3905 | 1.5069 |
|  |  | 20.4095 | 1.6434 |
|  |  | 20.4111 | -7.5393 |
|  |  | 20.4433 | -5.4213 |
|  |  | 20.4567 | -0.1014 |
|  |  | 20.4767 | 2.1241 |
|  |  | 20.4823 | 1.3333 |
|  |  | 20.4841 | -2.2376 |
|  |  | 20.5029 | -3.3645 |
|  |  | 20.5152 | 0.0687 |
|  |  | 20.5158 | 0.6666 |
|  |  | 20.5385 | 3.8603 |
|  |  | 20.5385 | 4.3605 |
|  |  | 20.5393 | -6.6211 |
|  |  | 20.5578 | -5.5456 |
|  |  | 20.5671 | -3.0973 |
|  |  | 20.5765 | -7.8579 |
|  |  | 20.5837 | 0.0035 |
|  |  | 20.5892 | 0.2937 |
|  |  | 20.5909 | -9.645 |
|  |  | 20.6208 | 3.4281 |
|  |  | 20.6222 | -3.79 |
|  |  | 20.6266 | -5.8309 |
|  |  | 20.633 | -1.1794 |
|  |  | 20.6425 | 1.6843 |
|  |  | 20.643 | 3.7366 |
|  |  | 20.6438 | -7.2894 |
|  |  | 20.6514 | -1.9922 |
|  |  | 20.6958 | -12.098 |
|  |  | 20.7043 | 0.9582 |
|  |  | 20.7066 | -2.4373 |
|  |  | 20.7069 | -2.0016 |
|  |  | 20.7155 | -4.2624 |
|  |  | 20.7168 | -5.1638 |
|  |  | 20.7329 | -4.9897 |
|  |  | 20.7346 | 0.1306 |
|  |  | 20.7391 | 0.0033 |
|  |  | 20.7566 | -5.7174 |
|  |  | 20.7644 | -3.4522 |
|  |  | 20.7973 | -14.0881 |
|  |  | 20.8222 | -5.193 |
|  |  | 20.8312 | 0.8485 |
|  |  | 20.837 | -1.0917 |
|  |  | 20.8563 | -1.0647 |
|  |  | 20.8603 | -4.5855 |
|  |  | 20.8649 | -5.7064 |
|  |  | 20.8728 | 0.0555 |
|  |  | 20.8948 | -0.2443 |
|  |  | 20.9099 | -0.2294 |
|  |  | 20.9116 | -1.0999 |
|  |  | 20.9172 | 0.0199 |
|  |  | 20.9383 | -3.7654 |
|  |  | 20.939 | -5.0015 |
|  |  | 20.9403 | -0.1389 |
|  |  | 20.9423 | -6.8232 |
|  |  | 20.9469 | 6.4734 |
|  |  | 20.9518 | 0.7967 |
|  |  | 20.9828 | -6.4975 |
|  |  | 20.99 | -14.8445 |
|  |  | 21.0073 | -0.0437 |
|  |  | 21.0114 | 0.3178 |
|  |  | 21.0129 | -1.0758 |
|  |  | 21.038 | -1.9316 |
|  |  | 21.0389 | 0.9188 |
|  |  | 21.0425 | -0.2895 |
|  |  | 21.0607 | -0.0009 |
|  |  | 21.0824 | 2.3329 |
|  |  | 21.0892 | -0.5408 |
|  |  | 21.1103 | -1.9798 |
|  |  | 21.1169 | -0.0525 |
|  |  | 21.1188 | 0.0266 |
|  |  | 21.1347 | -6.3161 |
|  |  | 21.1554 | -0.8666 |
|  |  | 21.1557 | 0.2753 |
|  |  | 21.1633 | -1.5891 |
|  |  | 21.1879 | -0.843 |
|  |  | 21.2157 | -1.0742 |
|  |  | 21.2158 | -1.2445 |
|  |  | 21.2442 | -6.9574 |
|  |  | 21.2545 | 1.3434 |
|  |  | 21.272 | 1.0303 |
|  |  | 21.2785 | 0.7026 |
|  |  | 21.2853 | 2.9734 |
|  |  | 21.2872 | 1.8477 |
|  |  | 21.2911 | 2.4125 |
|  |  | 21.3132 | -1.2172 |
|  |  | 21.3213 | 0.1943 |
|  |  | 21.3375 | -1.8958 |
|  |  | 21.3499 | -1.6282 |
|  |  | 21.3499 | -2.6088 |
|  |  | 21.3563 | -4.6714 |
|  |  | 21.3576 | -3.7951 |
|  |  | 21.365 | -0.3604 |
|  |  | 21.3963 | 6.9085 |
|  |  | 21.3989 | -1.5472 |
|  |  | 21.4059 | 3.3327 |
|  |  | 21.4441 | -0.2329 |
|  |  | 21.4608 | 2.5312 |
|  |  | 21.4908 | 0.9572 |
|  |  | 21.4994 | -1.3165 |
|  |  | 21.503 | 2.7089 |
|  |  | 21.5045 | 0.5627 |
|  |  | 21.5061 | 2.5835 |
|  |  | 21.5078 | 0.0464 |
|  |  | 21.523 | 0.2 |
|  |  | 21.5473 | -0.5867 |
|  |  | 21.5532 | 9.8367 |
|  |  | 21.562 | -1.542 |
|  |  | 21.5696 | 11.0818 |
|  |  | 21.5737 | 3.9908 |
|  |  | 21.5747 | 3.4183 |
|  |  | 21.5777 | 1.6887 |
|  |  | 21.5792 | 0.3701 |
|  |  | 21.5863 | -4.4918 |
|  |  | 21.5995 | -1.9743 |
|  |  | 21.6021 | 1.5226 |
|  |  | 21.6158 | 2.4246 |
|  |  | 21.6222 | -1.4428 |
|  |  | 21.6238 | -0.0785 |
|  |  | 21.6349 | 1.4783 |
|  |  | 21.6454 | 0.1879 |
|  |  | 21.6496 | -0.9599 |
|  |  | 21.6538 | 7.7764 |
|  |  | 21.6551 | 2.1625 |
|  |  | 21.6607 | 4.3384 |
|  |  | 21.6718 | 5.2463 |
|  |  | 21.6807 | 4.1664 |
|  |  | 21.6839 | -1.0475 |
|  |  | 21.6884 | -3.1288 |
|  |  | 21.7014 | 1.9218 |
|  |  | 21.7076 | -0.8607 |
|  |  | 21.7406 | 9.5058 |
|  |  | 21.7764 | 0.3742 |
|  |  | 21.7904 | 3.8605 |
|  |  | 21.7967 | 5.5379 |
|  |  | 21.7971 | -0.1576 |
|  |  | 21.7988 | 3.276 |
|  |  | 21.8181 | 3.1843 |
|  |  | 21.8251 | -3.983 |
|  |  | 21.8338 | -2.4075 |
|  |  | 21.847 | 0.1293 |
|  |  | 21.8498 | 0.2154 |
|  |  | 21.8677 | -1.7589 |
|  |  | 21.8723 | 1.6992 |
|  |  | 21.8915 | -0.8838 |
|  |  | 21.8945 | 7.3458 |
|  |  | 21.9044 | 4.0526 |
|  |  | 21.9052 | 10.1094 |
|  |  | 21.9163 | 1.3613 |
|  |  | 21.9166 | -2.2063 |
|  |  | 21.9241 | -5.6252 |
|  |  | 21.9337 | 9.4899 |
|  |  | 21.9539 | 0.3519 |
|  |  | 21.9698 | 12.3617 |
|  |  | 21.9707 | 0.0039 |
|  |  | 21.9708 | 3.4431 |
|  |  | 21.9712 | 18.7912 |
|  |  | 21.9741 | 4.0318 |
|  |  | 21.9854 | 11.2151 |
|  |  | 22.0055 | -0.3569 |
|  |  | 22.006 | 16.0836 |
|  |  | 22.0223 | -8.7163 |
|  |  | 22.0342 | -9.4489 |
|  |  | 22.0559 | 2.7264 |
|  |  | 22.0592 | 5.8661 |
|  |  | 22.0607 | 5.6044 |
|  |  | 22.0792 | 4.4278 |
|  |  | 22.0836 | 1.1936 |
|  |  | 22.0892 | -4.1543 |
|  |  | 22.0904 | 8.3581 |
|  |  | 22.0961 | -3.564 |
|  |  | 22.0972 | 4.9074 |
|  |  | 22.1055 | 2.0027 |
|  |  | 22.1255 | 7.2164 |
|  |  | 22.1327 | 1.154 |
|  |  | 22.1349 | -0.6136 |
|  |  | 22.1882 | 0.8656 |
|  |  | 22.1901 | -0.0716 |
|  |  | 22.1943 | 16.5545 |
|  |  | 22.2022 | 3.4122 |
|  |  | 22.2063 | -6.0814 |
|  |  | 22.219 | 5.616 |
|  |  | 22.2227 | -4.9068 |
|  |  | 22.2268 | -1.4065 |
|  |  | 22.2307 | 0.4209 |
|  |  | 22.2406 | 14.1964 |
|  |  | 22.25 | 14.3449 |
|  |  | 22.2525 | 0.6939 |
|  |  | 22.2585 | -6.0812 |
|  |  | 22.2651 | 3.8272 |
|  |  | 22.2811 | -0.3574 |
|  |  | 22.2843 | 10.7854 |
|  |  | 22.2931 | 2.6951 |
|  |  | 22.294 | 3.7053 |
|  |  | 22.2945 | 5.3237 |
|  |  | 22.2954 | 15.5196 |
|  |  | 22.3315 | -3.8946 |
|  |  | 22.3368 | 11.2576 |
|  |  | 22.3486 | -6.9453 |
|  |  | 22.3507 | 9.6731 |
|  |  | 22.3704 | 2.4603 |
|  |  | 22.3841 | 15.9989 |
|  |  | 22.3885 | -1.244 |
|  |  | 22.3971 | -5.0446 |
|  |  | 22.3998 | -1.6994 |
|  |  | 22.4113 | 15.6992 |
|  |  | 22.4177 | 0.0799 |
|  |  | 22.4187 | 9.3521 |
|  |  | 22.4242 | 5.9512 |
|  |  | 22.4291 | 2.6851 |
|  |  | 22.4441 | 13.6585 |
|  |  | 22.4448 | 0.2653 |
|  |  | 22.506 | 9.5182 |
|  |  | 22.5228 | -7.2327 |
|  |  | 22.5406 | -4.8587 |
|  |  | 22.592 | 13.6352 |
|  |  | 22.5963 | 8.6228 |
|  |  | 22.6204 | 2.534 |
|  |  | 22.6221 | -2.9295 |
|  |  | 22.6245 | -1.8884 |
|  |  | 22.6359 | 6.3381 |
|  |  | 22.6381 | -1.7633 |
|  |  | 22.6432 | -5.3955 |
|  |  | 22.6584 | 18.4729 |
|  |  | 22.6657 | 0.7727 |
|  |  | 22.6793 | -3.3178 |
|  |  | 22.6806 | 12.1625 |
|  |  | 22.6975 | 7.3387 |
|  |  | 22.7182 | 3.1316 |
|  |  | 22.7207 | 2.458 |
|  |  | 22.7209 | 14.5971 |
|  |  | 22.7353 | 7.0651 |
|  |  | 22.741 | 10.1279 |
|  |  | 22.7419 | 13.9265 |
|  |  | 22.7443 | -9.9637 |
|  |  | 22.7582 | 16.9864 |
|  |  | 22.771 | -5.3094 |
|  |  | 22.7835 | 3.1826 |
|  |  | 22.8065 | 0.8765 |
|  |  | 22.8076 | 5.5412 |
|  |  | 22.8237 | 7.3858 |
|  |  | 22.827 | -8.2283 |
|  |  | 22.8429 | -0.5158 |
|  |  | 22.8433 | -2.59 |
|  |  | 22.8453 | 17.8873 |
|  |  | 22.8461 | 13.2718 |
|  |  | 22.8589 | 1.8323 |
|  |  | 22.8868 | 6.2641 |
|  |  | 22.889 | 7.5023 |
|  |  | 22.9085 | 3.9983 |
|  |  | 22.9089 | -0.198 |
|  |  | 22.9224 | 1.5408 |
|  |  | 22.9343 | 10.4015 |
|  |  | 22.9563 | 9.3619 |
|  |  | 22.9654 | 5.5024 |
|  |  | 22.9869 | 4.8153 |
|  |  | 22.9911 | 11.7728 |
|  |  | 22.9923 | 9.4677 |
|  |  | 22.9969 | 8.8612 |
|  |  | 23.023 | -0.7732 |
|  |  | 23.0359 | 8.4166 |
|  |  | 23.0413 | 4.9871 |
|  |  | 23.0434 | 11.5004 |
|  |  | 23.0536 | 5.4494 |
|  |  | 23.0638 | 11.1165 |
|  |  | 23.067 | 4.8885 |
|  |  | 23.0985 | 7.8051 |
|  |  | 23.109 | 11.1905 |
|  |  | 23.1098 | 11.3288 |
|  |  | 23.1119 | 9.7032 |
|  |  | 23.1395 | 7.0187 |
|  |  | 23.143 | 16.1759 |
|  |  | 23.1479 | 2.3229 |
|  |  | 23.1835 | 5.2925 |
|  |  | 23.1914 | 1.8077 |
|  |  | 23.2094 | 15.9829 |
|  |  | 23.2111 | 9.5864 |
|  |  | 23.2256 | 6.5128 |
|  |  | 23.231 | 5.5157 |
|  |  | 23.2459 | 9.3095 |
|  |  | 23.2624 | 6.9254 |
|  |  | 23.2737 | 4.2946 |
|  |  | 23.28 | 4.3198 |
|  |  | 23.298 | 11.472 |
|  |  | 23.3109 | 6.3127 |
|  |  | 23.3407 | -0.7172 |
|  |  | 23.3488 | 5.4406 |
|  |  | 23.3517 | 10.5051 |
|  |  | 23.3671 | 5.8728 |
|  |  | 23.3807 | 5.1949 |
|  |  | 23.3836 | 8.5628 |
|  |  | 23.385 | 2.6014 |
|  |  | 23.3853 | 5.0527 |
|  |  | 23.3915 | 5.9089 |
|  |  | 23.4039 | 13.7987 |
|  |  | 23.4214 | 3.9928 |
|  |  | 23.4352 | 2.0385 |
|  |  | 23.4376 | 6.4979 |
|  |  | 23.4378 | 6.1866 |
|  |  | 23.4391 | 6.1845 |
|  |  | 23.4442 | 9.747 |
|  |  | 23.4462 | 4.9862 |
|  |  | 23.4551 | 12.4363 |
|  |  | 23.4632 | 6.5975 |
|  |  | 23.4752 | 11.7855 |
|  |  | 23.4849 | 6.726 |
|  |  | 23.494 | 6.8501 |
|  |  | 23.502 | -3.9161 |
|  |  | 23.5338 | 9.2114 |
|  |  | 23.5374 | 3.0697 |
|  |  | 23.5382 | 9.9813 |
|  |  | 23.5588 | 2.4004 |
|  |  | 23.5814 | 10.5173 |
|  |  | 23.5829 | 8.3224 |
|  |  | 23.5859 | 0.4939 |
|  |  | 23.6717 | 3.4045 |
|  |  | 23.6883 | 5.5824 |
|  |  | 23.7302 | 8.1341 |
|  |  | 23.7329 | -2.6688 |
|  |  | 23.7388 | 11.4641 |
|  |  | 23.7476 | 2.2763 |
|  |  | 23.7524 | 6.0073 |
|  |  | 23.7537 | 0.9942 |
|  |  | 23.7737 | 6.5413 |
|  |  | 23.7769 | 2.3146 |
|  |  | 23.7821 | 8.517 |
|  |  | 23.7848 | 2.6516 |
|  |  | 23.7872 | 6.2826 |
|  |  | 23.7962 | 5.8896 |
|  |  | 23.8066 | 5.5169 |
|  |  | 23.8067 | 8.3896 |
|  |  | 23.8191 | 6.337 |
|  |  | 23.8294 | -0.34 |
|  |  | 23.8307 | 7.8504 |
|  |  | 23.8369 | 8.8198 |
|  |  | 23.8385 | 2.7891 |
|  |  | 23.8844 | 1.0283 |
|  |  | 23.8851 | 7.5278 |
|  |  | 23.9172 | 3.0472 |
|  |  | 23.9248 | 5.9497 |
|  |  | 23.9315 | 1.5824 |
|  |  | 23.9391 | 2.5315 |
|  |  | 23.9418 | -3.0275 |
|  |  | 23.9496 | 7.5147 |
|  |  | 23.9616 | 2.1646 |
|  |  | 23.9642 | 7.6525 |
|  |  | 23.9659 | 2.598 |
|  |  | 23.9794 | 1.2953 |
|  |  | 24.0145 | 11.5284 |
|  |  | 24.0214 | 7.121 |
|  |  | 24.0227 | -1.168 |
|  |  | 24.0246 | 3.0163 |
|  |  | 24.0256 | 5.9032 |
|  |  | 24.0284 | 2.9462 |
|  |  | 24.0311 | -1.7038 |
|  |  | 24.0319 | 6.418 |
|  |  | 24.049 | 4.0365 |
|  |  | 24.0636 | 0.4777 |
|  |  | 24.0723 | -2.1555 |
|  |  | 24.0758 | 4.268 |
|  |  | 24.0765 | 3.6468 |
|  |  | 24.0818 | 2.1737 |
|  |  | 24.0883 | -1.2393 |
|  |  | 24.0884 | -1.2768 |
|  |  | 24.0964 | 4.293 |
|  |  | 24.1212 | -1.3388 |
|  |  | 24.1241 | -1.2107 |
|  |  | 24.1252 | -1.2235 |
|  |  | 24.1271 | -1.4104 |
|  |  | 24.1546 | 1.9864 |
|  |  | 24.1642 | 9.3341 |
|  |  | 24.1708 | -0.8495 |
|  |  | 24.1798 | 8.2666 |
|  |  | 24.1839 | 5.0758 |
|  |  | 24.1843 | -1.2324 |
|  |  | 24.1945 | -5.0208 |
|  |  | 24.1977 | -1.4144 |
|  |  | 24.2212 | -4.3816 |
|  |  | 24.2252 | 3.3286 |
|  |  | 24.2264 | 2.2089 |
|  |  | 24.2343 | 1.2891 |
|  |  | 24.2422 | -3.2801 |
|  |  | 24.2672 | -3.6797 |
|  |  | 24.271 | -1.4521 |
|  |  | 24.2806 | 4.4134 |
|  |  | 24.284 | 4.1493 |
|  |  | 24.2857 | 6.0308 |
|  |  | 24.2947 | 7.2215 |
|  |  | 24.304 | 3.4563 |
|  |  | 24.3175 | 2.9226 |
|  |  | 24.3196 | 5.9073 |
|  |  | 24.3313 | 0.0582 |
|  |  | 24.3365 | 4.2119 |
|  |  | 24.3376 | 2.7117 |
|  |  | 24.3393 | 5.6732 |
|  |  | 24.3684 | -3.0705 |
|  |  | 24.3783 | -6.171 |
|  |  | 24.3805 | -0.0612 |
|  |  | 24.3852 | -4.4403 |
|  |  | 24.4299 | 6.5542 |
|  |  | 24.436 | -0.4983 |
|  |  | 24.4588 | 0.667 |
|  |  | 24.4977 | -4.5759 |
|  |  | 24.5178 | -2.5198 |
|  |  | 24.5317 | 2.9003 |
|  |  | 24.5317 | -7.6892 |
|  |  | 24.5369 | -3.1167 |
|  |  | 24.5378 | -2.8148 |
|  |  | 24.5407 | 6.2859 |
|  |  | 24.5467 | 0.0416 |
|  |  | 24.554 | -7.9764 |
|  |  | 24.5581 | -3.5406 |
|  |  | 24.5723 | 2.9887 |
|  |  | 24.575 | -1.0215 |
|  |  | 24.5791 | -1.71 |
|  |  | 24.5853 | -1.1652 |
|  |  | 24.5881 | -1.0427 |
|  |  | 24.597 | -3.7098 |
|  |  | 24.6161 | 5.7931 |
|  |  | 24.6394 | -2.0879 |
|  |  | 24.6428 | 0.268 |
|  |  | 24.6673 | 5.6166 |
|  |  | 24.6892 | -3.5858 |
|  |  | 24.7054 | 0.6979 |
|  |  | 24.7109 | -4.914 |
|  |  | 24.733 | -5.5899 |
|  |  | 24.7355 | -4.4319 |
|  |  | 24.7462 | -2.3684 |
|  |  | 24.7712 | -15.7314 |
|  |  | 24.7743 | -6.5871 |
|  |  | 24.7748 | -6.1664 |
|  |  | 24.7894 | -6.0491 |
|  |  | 24.7906 | -3.6094 |
|  |  | 24.8158 | 5.3534 |
|  |  | 24.8169 | -9.2926 |
|  |  | 24.8249 | -5.1387 |
|  |  | 24.8258 | -8.2902 |
|  |  | 24.8259 | -4.02 |
|  |  | 24.8533 | -6.6862 |
|  |  | 24.8767 | -5.1329 |
|  |  | 24.8821 | -7.6934 |
|  |  | 24.8876 | -0.4533 |
|  |  | 24.8924 | -2.8556 |
|  |  | 24.893 | -6.8825 |
|  |  | 24.8992 | -4.1157 |
|  |  | 24.9029 | 2.6107 |
|  |  | 24.9052 | -8.653 |
|  |  | 24.9116 | 0.2266 |
|  |  | 24.912 | -1.6134 |
|  |  | 24.9196 | -4.2855 |
|  |  | 24.9247 | -1.7135 |
|  |  | 24.9327 | -0.9742 |
|  |  | 24.9472 | -13.9494 |
|  |  | 24.957 | -11.3288 |
|  |  | 25.0039 | -6.7802 |
|  |  | 25.0053 | 0.1298 |
|  |  | 25.0214 | -9.65 |
|  |  | 25.0726 | -5.2245 |
|  |  | 25.0759 | -6.4647 |
|  |  | 25.0825 | -2.3095 |
|  |  | 25.0886 | -9.5746 |
|  |  | 25.0887 | 1.1214 |
|  |  | 25.1356 | -12.1571 |
|  |  | 25.1437 | -6.8074 |
|  |  | 25.1649 | -4.9859 |
|  |  | 25.1816 | -7.7702 |
|  |  | 25.1893 | -10.1243 |
|  |  | 25.2555 | -8.5786 |
|  |  | 25.3127 | -10.578 |
|  |  | 25.316 | -10.1095 |
|  |  | 25.3429 | -4.9565 |
|  |  | 25.3868 | -4.2714 |
|  |  | 25.4127 | -8.9586 |
|  |  | 25.467 | -17.3185 |
|  |  | 25.4693 | 0.2908 |
|  |  | 25.4737 | -11.5452 |
|  |  | 25.4985 | -7.7217 |
|  |  | 25.5245 | -12.385 |
|  |  | 25.5512 | -10.6 |
|  |  | 25.5638 | -11.2926 |
|  |  | 25.5756 | -12.9282 |
|  |  | 25.6111 | -17.5899 |
|  |  | 25.6324 | -17.9325 |
|  |  | 25.6428 | -8.3007 |
|  |  | 25.6558 | -7.5883 |
|  |  | 25.6706 | -5.1255 |
|  |  | 25.7083 | -10.116 |
|  |  | 25.7318 | -13.633 |
|  |  | 25.752 | -11.5352 |
|  |  | 25.8058 | -16.5083 |
|  |  | 25.8351 | -10.9172 |
|  |  | 25.8547 | -15.5344 |
|  |  | 25.8904 | -12.5267 |
|  |  | 25.9092 | -14.5688 |
|  |  | 25.9103 | -7.1813 |
|  |  | 25.9307 | -3.6936 |
|  |  | 25.9517 | -14.8245 |
|  |  | 25.9822 | -15.115 |
|  |  | 26.0125 | -17.275 |
|  |  | 26.0269 | -6.58 |
|  |  | 26.0409 | -12.2432 |
|  |  | 26.0479 | -15.7721 |
|  |  | 26.0743 | -6.1154 |
|  |  | 26.0995 | -15.2471 |
|  |  | 26.135 | -22.9959 |
|  |  | 26.1435 | -10.0835 |
|  |  | 26.1586 | -13.4998 |
|  |  | 26.203 | -16.7748 |
|  |  | 26.2111 | -14.6838 |
|  |  | 26.2246 | -8.2143 |
|  |  | 26.2401 | -8.0344 |
|  |  | 26.2454 | -12.8687 |
|  |  | 26.3084 | -10.7549 |
|  |  | 26.3249 | -6.1222 |
|  |  | 26.3747 | -6.3145 |
|  |  | 26.3945 | -9.2805 |
|  |  | 26.4334 | -9.7519 |
|  |  | 26.482 | -14.4589 |
|  |  | 26.501 | -9.9688 |
|  |  | 26.5011 | -10.1223 |
|  |  | 26.5135 | -13.2984 |
|  |  | 26.5155 | -10.553 |
|  |  | 26.5557 | -7.2452 |
|  |  | 26.5598 | 2.235 |
|  |  | 26.5745 | -13.9657 |
|  |  | 26.5849 | -8.4663 |
|  |  | 26.5881 | -5.8724 |
|  |  | 26.6081 | -8.1068 |
|  |  | 26.6084 | -13.1461 |
|  |  | 26.6132 | -4.1165 |
|  |  | 26.6282 | 0.5724 |
|  |  | 26.6345 | -9.598 |
|  |  | 26.6412 | -14.3509 |
|  |  | 26.6475 | -5.6703 |
|  |  | 26.6547 | -0.9323 |
|  |  | 26.693 | -12.9664 |
|  |  | 26.7032 | -6.2342 |
|  |  | 26.707 | -10.9744 |
|  |  | 26.7723 | -2.158 |
|  |  | 26.7788 | -5.4141 |
|  |  | 26.7964 | -2.5674 |
|  |  | 26.7965 | -19.1196 |
|  |  | 26.8271 | -17.8599 |
|  |  | 26.8566 | -3.0552 |
|  |  | 26.8963 | -0.3278 |
|  |  | 26.9081 | -11.3786 |
|  |  | 26.9398 | -9.5757 |
|  |  | 26.9641 | -8.4579 |
|  |  | 26.9816 | -7.6247 |
|  |  | 27.0085 | -12.4902 |
|  |  | 27.0879 | -6.9755 |
|  |  | 27.0898 | -0.846 |
|  |  | 27.1313 | -4.9134 |
|  |  | 27.1378 | -0.7073 |
|  |  | 27.166 | 0.796 |
|  |  | 27.1744 | -11.1819 |
|  |  | 27.2421 | 0.1393 |
|  |  | 27.2599 | -8.2348 |
|  |  | 27.2811 | 9.7223 |
|  |  | 27.294 | -8.9196 |
|  |  | 27.3716 | -4.3179 |
|  |  | 27.4487 | 0.457 |
|  |  | 27.4515 | -5.982 |
|  |  | 27.4601 | -0.6035 |
|  |  | 27.4687 | 5.2204 |
|  |  | 27.4782 | 0.4051 |
|  |  | 27.4822 | 0.4421 |
|  |  | 27.5151 | 1.2855 |
|  |  | 27.6001 | -7.4156 |
|  |  | 27.6279 | -2.0177 |
|  |  | 27.6409 | 6.1002 |
|  |  | 27.6453 | 5.8634 |
|  |  | 27.65 | -1.2471 |
|  |  | 27.6805 | 0.2351 |
|  |  | 27.7052 | 2.2709 |
|  |  | 27.7456 | -2.1265 |
|  |  | 27.7547 | 1.2727 |
|  |  | 27.8263 | 3.0616 |
|  |  | 27.8301 | 9.666 |
|  |  | 27.8334 | 13.4139 |
|  |  | 27.9435 | 3.856 |
|  |  | 27.957 | -3.1166 |
|  |  | 27.9673 | 7.7379 |
|  |  | 27.991 | 10.6873 |
|  |  | 27.9992 | 7.1924 |
|  |  | 28.0362 | 11.666 |
|  |  | 28.1073 | 2.4325 |
|  |  | 28.1432 | 12.1979 |
|  |  | 28.2304 | 4.0727 |
|  |  | 28.2508 | 15.3507 |
|  |  | 28.3357 | 12.9502 |
|  |  | 28.3611 | 4.7326 |
|  |  | 28.372 | 2.9155 |
|  |  | 28.388 | 6.0728 |
|  |  | 28.4552 | -5.7818 |
|  |  | 28.476 | 4.6586 |
|  |  | 28.4819 | 8.2816 |
|  |  | 28.4847 | 7.005 |
|  |  | 28.501 | 5.6354 |
|  |  | 28.5317 | 9.8052 |
|  |  | 28.5567 | 19.3827 |
|  |  | 28.557 | 2.7578 |
|  |  | 28.5627 | 21.1599 |
|  |  | 28.5649 | 14.274 |
|  |  | 28.5758 | 10.7108 |
|  |  | 28.5825 | 2.8666 |
|  |  | 28.6104 | 8.6677 |
|  |  | 28.6115 | 8.4997 |
|  |  | 28.6162 | 8.0476 |
|  |  | 28.6518 | 14.4198 |
|  |  | 28.6531 | 9.2317 |
|  |  | 28.6688 | 16.9091 |
|  |  | 28.6999 | 9.2841 |
|  |  | 28.7144 | 16.0684 |
|  |  | 28.7195 | 13.9252 |
|  |  | 28.7468 | 0.4123 |
|  |  | 28.8154 | 24.4517 |
|  |  | 28.8155 | 19.0698 |
|  |  | 28.8202 | -3.643 |
|  |  | 28.8601 | 10.1689 |
|  |  | 28.9544 | 5.5919 |
|  |  | 29.0043 | 23.8859 |
|  |  | 29.0478 | 25.2947 |
|  |  | 29.0888 | 11.742 |
|  |  | 29.1063 | 7.4142 |
|  |  | 29.1075 | 15.3385 |
|  |  | 29.1352 | 7.3345 |
|  |  | 29.1451 | 27.8179 |
|  |  | 29.1606 | 11.0694 |
|  |  | 29.1613 | 16.5776 |
|  |  | 29.1926 | 10.461 |
|  |  | 29.2102 | 16.7286 |
|  |  | 29.3055 | 14.9915 |
|  |  | 29.3121 | 4.9105 |
|  |  | 29.3305 | 14.0702 |
|  |  | 29.3458 | 21.838 |
|  |  | 29.3526 | 13.2607 |
|  |  | 29.3742 | 26.2367 |
|  |  | 29.4213 | 19.52 |
|  |  | 29.49 | 19.2228 |
|  |  | 29.4934 | 15.516 |
|  |  | 29.5242 | 19.5352 |
|  |  | 29.5912 | 19.0835 |
|  |  | 29.6093 | 18.447 |
|  |  | 29.6824 | 10.3544 |
|  |  | 29.7411 | 12.1922 |
|  |  | 29.7607 | 23.6772 |
|  |  | 29.7796 | 19.2956 |
|  |  | 29.7913 | 1.5942 |
|  |  | 29.8144 | 5.8801 |
|  |  | 29.8213 | 18.5306 |
|  |  | 29.8241 | 10.8198 |
|  |  | 29.8921 | 13.3506 |
|  |  | 29.9173 | 11.9204 |
