# Supplementary material for: Synaptic plasticity-based regularizer for artificial neural networks
Source: Sci Rep. 2025 Apr 24;15:14330. doi: 10.1038/s41598-025-91635-3 (PMC12022306; doi:10.1038/s41598-025-91635-3)
Supplement: Supplementary file 2 — Supplementary Material 2 [file 41598_2025_91635_MOESM2_ESM.docx]

Type of the Paper (Research)

Title *Synaptic plasticity-based regularizer for artificial neural networks*

Qais Yousef ^1^,* & Pu Li ^2^,*

1 ORCID: [0000-0003-0239-9150].

2 ORCID: [0000-0001-6481-9961].

1,2 Group of Process Optimization, Institute for Automation and Systems Engineering, Technische Universität Ilmenau, P.O. Box 100565, 98684 Ilmenau, Germany

* Correspondence: {qais.yousef, pu.li}@tu-ilmenau.de

**Single Wave Principle: Theoretical Proof and Example**

**I. Single Wave Principle: a Theoretical Proof**

By applying the evolutionary algorithm given the problem (16.2) on the neuron level such as, $\xi_{l,i,t}^{(\tau+1)}=evolutionary algorithm \left( \xi_{l,i,t}^{\left( \tau\right)} \right)$ for iterations $0<\tau<T$, the algorithm will produce a sequence of vectors $\xi_{l,i,t}^{(0)}, \xi_{l,i,t}^{(1)}, \ldots, \xi_{l,i,t}^{(T)}$, where $\xi_{l,i,t}^{(T)}$ is the final minimized vector and $\boldsymbol{S}_{l,i,t}^{*}$ is its solution, which we prove as follows.

**Assumption A1:** ${\tilde{\dot{\hat{\boldsymbol{x}}}}}_{l-1,t}$ is the optimal final refined vector with optimal values obtained either from its fully connected layer or through the optimization in its layer.

**Assumption A2:** $\xi_{l-1,t}^{(T)}$ represents the inner cost of the previous layer, minimized either by employing ${\tilde{\dot{\hat{\boldsymbol{x}}}}}_{l-1,t}$ or from the optimization stage in its layer.

**Proposition 1:** The inner cost function $\xi_{l,i,t}^{(T)}$ is minimized by the evolutionary algorithm by solving (16.2), such that, $\xi_{l,i,t}^{(T)}\leq\xi_{l,i,t}^{(0)}$, resulting in the optimal solution vector $\boldsymbol{S}_{l,i,t}^{*}$.

**Proof 1:** Given that $\xi_{l,i,t}^{(T)}\leq\xi_{l,i,t}^{(0)}$ for each $\xi_{l,i,t}$, the mean at iteration $T$ in equation (13) is, $\xi_{l,t}^{(T)}$. It follows that $\xi_{l,t}^{(T)}\leq\xi_{l,t}^{(0)}$. Implying that the combinatory vector $\boldsymbol{S}_{l,t}^{(T)}$ has better values than $\boldsymbol{S}_{l,t}^{(0)}$. Deploying the solution $\boldsymbol{S}_{l,t}^{(T)}$ on the masks of the neurons of the corresponding layer and given A1 that ${\tilde{\dot{\hat{\boldsymbol{x}}}}}_{l-1,t}$ has the optimal refined values, results in improved refined values of the corresponding masks, i.e. $\boldsymbol{M}_{l,i}\left( \boldsymbol{S}_{l,i,t}^{*}.{\tilde{\dot{\hat{\boldsymbol{x}}}}}_{l-1,t} \right)={\tilde{\dot{\hat{\boldsymbol{M}}}}}_{l,i,t}^{\boldsymbol{(}T\boldsymbol{)}}$, which are more optimally refined than ${\tilde{\dot{\hat{\boldsymbol{M}}}}}_{l,i,t}^{\boldsymbol{(}0\boldsymbol{)}}$. This implies that the distance between any mask’s output and the mean of these masks is minimized, i.e. $\left| {\tilde{\dot{\hat{\boldsymbol{M}}}}}_{l,i,t}^{\boldsymbol{(}T\boldsymbol{)}}-\bar{{\tilde{\dot{\hat{\boldsymbol{M}}}}}_{l,i,t}^{\boldsymbol{(}T\boldsymbol{)}}} \right|\leq\left| {\tilde{\dot{\hat{\boldsymbol{M}}}}}_{l,i,t}^{\boldsymbol{(}0\boldsymbol{)}}-\bar{{\tilde{\dot{\hat{\boldsymbol{M}}}}}_{l,i,t}^{\boldsymbol{(}0\boldsymbol{)}}} \right|$. This means that the disparity of the masks is minimized, such as, $\sigma_{{\tilde{\dot{\hat{\boldsymbol{M}}}}}_{l,i,t}^{\boldsymbol{(}T\boldsymbol{)}}}\leq\sigma_{{\tilde{\dot{\hat{\boldsymbol{M}}}}}_{l,i,t}^{\boldsymbol{(}0\boldsymbol{)}}}$. Consequently the first condition of the weighted probability “If $\sigma_{M_{l,i,t}}<d_{l,i,t}$ meaning that the masks are more sure of their outputs than the original neuron, then $\hat{Pr}^{\mathcal{w}}\left( \hat{M}_{l,i,t} \right)>\hat{Pr}^{\mathcal{w}}\left( \hat{x}_{l,i,t} \right)$“ is satisfied. This indicates that $1-\hat{Pr}^{\mathcal{w}}\left( {\tilde{\dot{\hat{\boldsymbol{M}}}}}_{l,i,t}^{\boldsymbol{(}T\boldsymbol{)}} \right)$ is minimized. Applying (8) ensures that the corresponding neuron is refined with the best value obtained by its masks i.e. $\hat{x}_{l,i,t}\leftarrow{\tilde{\dot{\hat{x}}}}_{l,i,t}$, which implies that $1-\hat{Pr}^{\mathcal{w}}\left( {\tilde{\dot{\hat{x}}}}_{l,i,t} \right)$ is also minimized. Given that and hence from A2 the risk of the previous layer $\xi_{l-1,t}^{(T)}$ is minimized, this suggests that $\xi_{l,i,t}^{(T)}$ in (12) is minimized. Repeating this process on the masks of the remaining neurons results in a minimized mean of risks $\xi_{l,t}^{(T)}$ in (13), and its solution $\boldsymbol{S}_{l,t}^{(T)}$ is the global best, i.e. $\boldsymbol{S}_{l,t}^{*}=\left\{ \boldsymbol{S}_{l,i,t}^{*} \right\}_{i=1}^{N_{l}^{N}}$ .

**Proposition 2:** Finding the solutions $\boldsymbol{S}_{l,i,t}^{*}$ for all the neurons in $2<l<L$ solves the upper-level problem (16.1)

**Proof 2:** Given, from proof 1, that $\boldsymbol{S}_{l,t}^{*}=\left\{ \boldsymbol{S}_{l,i,t}^{*} \right\}_{i=1}^{N_{l}^{N}}$ is the optimal solution vector, applying this on the rest of the neurons in the layers $2<l<L$ results in optimally refined values of the neurons $\left\{ {\tilde{\dot{\hat{\boldsymbol{x}}}}}_{l,t} \right\}_{l=2}^{L}$. This includes the logits of the output layer i.e. $\left\{ {\tilde{\dot{\hat{x}}}}_{L,i,t} \right\}_{i=1}^{N_{L}^{N}}$.

**Supporting Evidence from Literature E1**: Given the features of prospect certainty (refer to Section 2.2.4 in [35]) and the properties of prospect theory in [38], and considering that the behavior function $b$ measures the Wasserstein distance between the original training distribution and the distribution of the model output influenced by the given alternative (i.e. logit) [35], then the logit ${\tilde{\dot{\hat{x}}}}_{L,i,t}$with the highest weighted probability will exhibit the maximum prospect certainty value.

Hence, the output ${\tilde{\dot{\hat{\boldsymbol{y}}}}}_{t}$ is calculated by (14) and (15) after selecting the logit with the highest weighted probability using (8). Considering that the prospect risk loss is the convex version of the prospect certainty, and in light of E1, employing (11) ensures that the final refined output ${\tilde{\dot{\hat{\boldsymbol{y}}}}}_{t}$results in the minimum risk, i.e. $\mathcal{L}_{t}^{r}\left( {\tilde{\dot{\hat{\boldsymbol{y}}}}}_{t} \right)$ in (16.1) is minimized .

The proofs above demonstrate that the single wave principle requires a single forward move to solve (16).

**II. Single Wave Principle: a Walkthrough Example**

A step-by-step walkthrough of the synaptic rewiring process for the example of the model mentioned in Fig 1.

Given that the first layer is fully connected with the input layer, i.e., $\boldsymbol{S}_{1,t}$ is known. The initial value ${\hat{\boldsymbol{x}}}_{1,t}={\hat{\boldsymbol{x}}}_{input,t}\left( \boldsymbol{u}_{t} \right)$ is calculated directly using the general formula of the neuron value,

$$\hat{x}_{l,i,t}=\sum_{\nu=1}^{\left[ \boldsymbol{u} \right]} {w_{x_{l,i},\nu}u}_{\nu}+b$$

Where $\left[ \boldsymbol{u} \right]$ is the input dimension and $w$ is the corresponding weight $w_{x_{l,i},\nu}$ on the connection between the neuron $x_{l,i}$ and the input $\nu$.

1. Applying this we get the initial values of the two neurons and their corresponding masks in the first layer.

$$\hat{x}_{1,1,t}=\sum_{\nu=1}^{2} {w_{x_{1,1},\nu}u}_{\nu}+b$$

$$\hat{m}_{1,1,1,t}=\sum_{\nu=1}^{2} {w_{m_{1,1,1},\nu}u}_{\nu}+b$$

$$\hat{m}_{1,1,2,t}=\sum_{\nu=1}^{2} {w_{m_{1,1,2},\nu}u}_{\nu}+b$$

$$\hat{m}_{1,1,3,t}=\sum_{\nu=1}^{2} {w_{m_{1,1,3},\nu}u}_{\nu}+b$$

$$\hat{x}_{1,2,t}=\sum_{\nu=1}^{2} {w_{x_{1,2},\nu}u}_{\nu}+b$$

$$\hat{m}_{1,2,1,t}=\sum_{\nu=1}^{2} {w_{m_{1,2,1},\nu}u}_{\nu}+b$$

$$\hat{m}_{1,2,2,t}=\sum_{\nu=1}^{2} {w_{m_{1,2,2},\nu}u}_{\nu}+b$$

$$\hat{m}_{1,2,3,t}=\sum_{\nu=1}^{2} {w_{m_{1,2,3},\nu}u}_{\nu}+b$$

$$\hat{x}_{1,3,t}=\sum_{\nu=1}^{2} {w_{x_{1,3},\nu}u}_{\nu}+b$$

$$\hat{m}_{1,3,1,t}=\sum_{\nu=1}^{2} {w_{m_{1,3,1},\nu}u}_{\nu}+b$$

$$\hat{m}_{1,3,2,t}=\sum_{\nu=1}^{2} {w_{m_{1,3,2},\nu}u}_{\nu}+b$$

$$\hat{m}_{1,3,3,t}=\sum_{\nu=1}^{2} {w_{m_{1,3,3},\nu}u}_{\nu}+b$$

$$\hat{x}_{1,4,t}=\sum_{\nu=1}^{2} {w_{x_{1,4},\nu}u}_{\nu}+b$$

$$\hat{m}_{1,4,1,t}=\sum_{\nu=1}^{2} {w_{m_{1,4,1},\nu}u}_{\nu}+b$$

$$\hat{m}_{1,4,2,t}=\sum_{\nu=1}^{2} {w_{m_{1,4,2},\nu}u}_{\nu}+b$$

$$\hat{m}_{1,4,3,t}=\sum_{i=\nu}^{2} {w_{m_{1,4,3},\nu}u}_{\nu}+b$$

2. The refined logit values of layer 1 are calculated using (8) as,

$${\dot{\hat{x}}}_{1,1,t}=argmax\left( \hat{Pr}^{\mathcal{w}}\left( \hat{x}_{1,1,t} \right),\left\{ \hat{Pr}^{\mathcal{w}}\left( \hat{m}_{1,1,j,t} \right) \right\}_{j=1}^{3} \right)$$

$${\dot{\hat{x}}}_{1,2,t}=argmax\left( \hat{Pr}^{\mathcal{w}}\left( \hat{x}_{1,2,t} \right),\left\{ \hat{Pr}^{\mathcal{w}}\left( \hat{m}_{1,2,j,t} \right) \right\}_{j=1}^{3} \right)$$

$${\dot{\hat{x}}}_{1,3,t}=argmax\left( \hat{Pr}^{\mathcal{w}}\left( \hat{x}_{1,3,t} \right),\left\{ \hat{Pr}^{\mathcal{w}}\left( \hat{m}_{1,3,j,t} \right) \right\}_{j=1}^{3} \right)$$

$${\dot{\hat{x}}}_{1,4,t}=argmax\left( \hat{Pr}^{\mathcal{w}}\left( \hat{x}_{1,4,t} \right),\left\{ \hat{Pr}^{\mathcal{w}}\left( \hat{m}_{1,4,j,t} \right) \right\}_{j=1}^{3} \right)$$

3. Then the vector of the neurons for the first layer is,

$${\dot{\hat{\boldsymbol{x}}}}_{1,t}=[{\dot{\hat{x}}}_{1,1,t},{\dot{\hat{x}}}_{1,2,t},{\dot{\hat{x}}}_{1,3,t},{\dot{\hat{x}}}_{1,4,t}]$$

The steps from 1 to 3, will be the same in the following layers to calculate the values of their neurons, considering the vector of the solution variables $\boldsymbol{S}_{l,j,t}$ for their corresponding neurons.

1. The initial risks of the neurons in the first layer are found directly by utilizing the weighted probability as,

$$\xi_{1,1,t}=1-\left\{ \hat{Pr}^{\mathcal{w}}\left( {\dot{\hat{x}}}_{1,1,t} \right) \right\}_{j=1}^{3}$$

$$\xi_{1,2,t}=1-\left\{ \hat{Pr}^{\mathcal{w}}\left( {\dot{\hat{x}}}_{1,2,t} \right) \right\}_{j=1}^{3}$$

$$\xi_{1,3,t}=1-\left\{ \hat{Pr}^{\mathcal{w}}\left( {\dot{\hat{x}}}_{1,3,t} \right) \right\}_{j=1}^{3}$$

$$\xi_{1,4,t}=1-\left\{ \hat{Pr}^{\mathcal{w}}\left( {\dot{\hat{x}}}_{1,4,t} \right) \right\}_{j=1}^{3}$$

2. Then the vector of the risks for the first layer is,

$$\xi_{1,t}=\frac{\sum_{i=1}^{4} \xi_{1,i,t}}{4}$$

3. The local cost (12) for the neurons of the second layer is calculated as follows, considering $\boldsymbol{S}_{2,j,t}$to be the vector of the solution variables.

$$\xi_{2,1,t}\left( M_{2,1}\left( \boldsymbol{S}_{2,1,t}. {\dot{\hat{\boldsymbol{x}}}}_{1,t} \right),\delta_{2} \right)=\left( 1-\delta_{2} \right)\left( 1-\hat{Pr}^{\mathcal{w}}\left( {\dot{\hat{x}}}_{2,1,t} \right) \right)+\delta_{2}\xi_{1,t}$$

$$\xi_{2,2,t}\left( M_{2,2}\left( \boldsymbol{S}_{2,2,t}. {\dot{\hat{\boldsymbol{x}}}}_{1,t} \right),\delta_{2} \right)=\left( 1-\delta_{2} \right)\left( 1-\hat{Pr}^{\mathcal{w}}\left( {\dot{\hat{x}}}_{2,2,t} \right) \right)+\delta_{2}\xi_{1,t}$$

$$\xi_{2,3,t}\left( M_{2,3}\left( \boldsymbol{S}_{2,3,t}. {\dot{\hat{\boldsymbol{x}}}}_{1,t} \right),\delta_{2} \right)=\left( 1-\delta_{2} \right)\left( 1-\hat{Pr}^{\mathcal{w}}\left( {\dot{\hat{x}}}_{2,3,t} \right) \right)+\delta_{2}\xi_{1,t}$$

$$\xi_{2,4,t}\left( M_{2,4}\left( \boldsymbol{S}_{2,4,t}. {\dot{\hat{\boldsymbol{x}}}}_{1,t} \right),\delta_{2} \right)=\left( 1-\delta_{2} \right)\left( 1-\hat{Pr}^{\mathcal{w}}\left( {\dot{\hat{x}}}_{2,4,t} \right) \right)+\delta_{2}\xi_{1,t}$$

4. The mean of the risks for the second layer is calculated using (13) as,

$$\xi_{2,t}\left( \boldsymbol{M}_{2}\left( \boldsymbol{S}_{2,t}.{\dot{\hat{\boldsymbol{x}}}}_{1,t} \right),\delta_{2} \right)=\frac{\sum_{i=1}^{4} \xi_{2,i,t}\left( M_{2,i}\left( \boldsymbol{S}_{2,i,t}.{\dot{\hat{\boldsymbol{x}}}}_{1,t} \right),\delta_{2} \right)}{4}$$

5. Repeating steps 7 and 8 by the evolutionary algorithm by employing its agents simultaneously results in the optimal solution of (16.2), as,

$$\boldsymbol{S}_{2,t}^{*}=\left\{ \boldsymbol{S}_{2,i,t}^{*} \right\}_{i=1}^{4}=\underset{\boldsymbol{S}_{2,t}}{\mathrm{argmin}} \xi_{2,t}\left( \boldsymbol{M}_{2}\left( \boldsymbol{S}_{2,t}.{\dot{\hat{\boldsymbol{x}}}}_{1,t} \right),\delta_{2} \right)$$

6. The local cost (12) for the neurons of the third layer is then calculated as follows, considering $\boldsymbol{S}_{3,j,t}$to be the vector of the solution variables.

$$\xi_{3,1,t}\left( M_{3,1}\left( \boldsymbol{S}_{3,1,t}. {\dot{\hat{\boldsymbol{x}}}}_{2,t} \right),\delta_{3} \right)=\left( 1-\delta_{3} \right)\left( 1-\hat{Pr}^{\mathcal{w}}\left( {\dot{\hat{x}}}_{3,1,t} \right) \right)+\delta_{3}\xi_{2,t}$$

$$\xi_{3,2,t}\left( M_{3,2}\left( \boldsymbol{S}_{3,2,t}. {\dot{\hat{\boldsymbol{x}}}}_{2,t} \right),\delta_{3} \right)=\left( 1-\delta_{3} \right)\left( 1-\hat{Pr}^{\mathcal{w}}\left( {\dot{\hat{x}}}_{3,2,t} \right) \right)+\delta_{3}\xi_{2,t}$$

$$\xi_{3,3,t}\left( M_{3,3}\left( \boldsymbol{S}_{3,3,t}. {\dot{\hat{\boldsymbol{x}}}}_{2,t} \right),\delta_{3} \right)=\left( 1-\delta_{3} \right)\left( 1-\hat{Pr}^{\mathcal{w}}\left( {\dot{\hat{x}}}_{3,3,t} \right) \right)+\delta_{3}\xi_{2,t}$$

7. The mean of the risks for the third layer is calculated using (13) as,

$$\xi_{3,t}\left( \boldsymbol{M}_{3}\left( \boldsymbol{S}_{3,t}.{\dot{\hat{\boldsymbol{x}}}}_{2,t} \right),\delta_{3} \right)=\frac{\sum_{i=1}^{3} \xi_{3,i,t}\left( M_{3,i}\left( \boldsymbol{S}_{3,i,t}.{\dot{\hat{\boldsymbol{x}}}}_{2,t} \right),\delta_{3} \right)}{3}$$

8. Repeating steps 9 and 10 by the evolutionary algorithm by employing its agents simultaneously results in the optimal solution of (16.2), as,

$$\boldsymbol{S}_{3,t}^{*}=\left\{ \boldsymbol{S}_{3,i,t}^{*} \right\}_{i=1}^{3}=\left( 1-\delta_{3} \right)\underset{\boldsymbol{S}_{3,t}}{\mathrm{argmin}} \xi_{3,t}\left( \boldsymbol{M}_{3}\left( \boldsymbol{S}_{3,t}.{\dot{\hat{\boldsymbol{x}}}}_{2,t} \right),\delta_{3} \right)+\delta_{3}\left[ \underset{\boldsymbol{S}_{2,t}}{\mathrm{argmin}} \xi_{2,t}\left( \boldsymbol{M}_{2}\left( \boldsymbol{S}_{2,t}.{\dot{\hat{\boldsymbol{x}}}}_{1,t} \right),\delta_{2} \right) \right]$$

where $\left[ \underset{\boldsymbol{S}_{2,t}}{\mathrm{argmin}} \xi_{2,t}\left( \boldsymbol{M}_{2}\left( \boldsymbol{S}_{2,t}.{\dot{\hat{\boldsymbol{x}}}}_{1,t} \right),\delta_{2} \right) \right]=\boldsymbol{S}_{2,t}^{*}$

Therefore we can rewrite the problem in step 11 as,

$$\boldsymbol{S}_{3,t}^{*}=\left\{ \boldsymbol{S}_{3,i,t}^{*} \right\}_{i=1}^{3}=\left( 1-\delta_{3} \right)\underset{\boldsymbol{S}_{3,t}}{\mathrm{argmin}} \xi_{3,t}\left( \boldsymbol{M}_{3}\left( \boldsymbol{S}_{3,t}.{\dot{\hat{\boldsymbol{x}}}}_{2,t} \right),\delta_{3} \right)+\delta_{3}\boldsymbol{S}_{2,t}^{*}$$

1. The local cost (12) for the neurons of the fourth (output) layer is then calculated as follows, considering $\boldsymbol{S}_{4,j,t}$to be the vector of the solution variables.

$$\xi_{4,1,t}\left( M_{4,1}\left( \boldsymbol{S}_{4,1,t}. {\dot{\hat{\boldsymbol{x}}}}_{3,t} \right),\delta_{4} \right)=\left( 1-\delta_{4} \right)\left( 1-\hat{Pr}^{\mathcal{w}}\left( {\dot{\hat{x}}}_{4,1,t} \right) \right)+\delta_{4}\xi_{3,t}$$

2. Since it is the only neuron in this layer, the mean of the risks for the last layer is calculated using (13) as,

$$\xi_{4,t}\left( \boldsymbol{M}_{4}\left( \boldsymbol{S}_{4,t}.{\dot{\hat{\boldsymbol{x}}}}_{3,t} \right),\delta_{4} \right)=\xi_{4,1,t}\left( M_{4,1}\left( \boldsymbol{S}_{4,1,t}. {\dot{\hat{\boldsymbol{x}}}}_{3,t} \right),\delta_{4} \right)$$

3. Repeating steps 12 and 13 by the evolutionary algorithm by employing its agents simultaneously results in the optimal solution of (16.2), as,

$$\boldsymbol{S}_{4,t}^{*}=\boldsymbol{S}_{4,i,t}^{*}=\left( 1-\delta_{4} \right)\underset{\boldsymbol{S}_{4,t}}{\mathrm{argmin}} \xi_{4,t}\left( \boldsymbol{M}_{4}\left( \boldsymbol{S}_{4,t}.{\dot{\hat{\boldsymbol{x}}}}_{3,t} \right),\delta_{4} \right)+\delta_{4}\left[ \left( 1-\delta_{3} \right)\underset{\boldsymbol{S}_{3,t}}{\mathrm{argmin}} \xi_{3,t}\left( \boldsymbol{M}_{3}\left( \boldsymbol{S}_{3,t}.{\dot{\hat{\boldsymbol{x}}}}_{2,t} \right),\delta_{3} \right)+\delta_{3}\left[ \underset{\boldsymbol{S}_{2,t}}{\mathrm{argmin}} \xi_{2,t}\left( \boldsymbol{M}_{2}\left( \boldsymbol{S}_{2,t}.{\dot{\hat{\boldsymbol{x}}}}_{1,t} \right),\delta_{2} \right) \right] \right]$$

where $\left[ \left( 1-\delta_{3} \right)\underset{\boldsymbol{S}_{3,t}}{\mathrm{argmin}} \xi_{3,t}\left( \boldsymbol{M}_{3}\left( \boldsymbol{S}_{3,t}.{\dot{\hat{\boldsymbol{x}}}}_{2,t} \right),\delta_{3} \right)+\delta_{3}\left[ \underset{\boldsymbol{S}_{2,t}}{\mathrm{argmin}} \xi_{2,t}\left( \boldsymbol{M}_{2}\left( \boldsymbol{S}_{2,t}.{\dot{\hat{\boldsymbol{x}}}}_{1,t} \right),\delta_{2} \right) \right] \right]=\boldsymbol{S}_{3,t}^{*}$

Therefore, we can rewrite the formulation in step 14 as,

$$\boldsymbol{S}_{4,t}^{*}=\boldsymbol{S}_{4,i,t}^{*}=\left( 1-\delta_{4} \right)\underset{\boldsymbol{S}_{4,t}}{\mathrm{argmin}} \xi_{4,t}\left( \boldsymbol{M}_{4}\left( \boldsymbol{S}_{4,t}.{\dot{\hat{\boldsymbol{x}}}}_{3,t} \right),\delta_{4} \right)+\delta_{4}\boldsymbol{S}_{3,t}^{*}$$

1. The complete synaptic connections vector is then accumulated as,

$$\boldsymbol{S}_{t}^{*}=\left[ \boldsymbol{S}_{2,t}^{*},\boldsymbol{S}_{3,t}^{*},\boldsymbol{S}_{4,t}^{*} \right]$$

2. Obtaining the complete synaptic connections structure $\boldsymbol{S}_{t}^{*}$, allows us to calculate the final output using (14) and (15) as,

${\tilde{\dot{\hat{\boldsymbol{x}}}}}_{t}=f\left( \boldsymbol{u}_{t},\boldsymbol{\theta}\boldsymbol{,S}_{t}^{*} \right)$

${\tilde{\dot{\hat{\boldsymbol{y}}}}}_{t}=g({\tilde{\dot{\hat{\boldsymbol{x}}}}}_{t})$

1. Which allows measuring its output prospect risk (11) as,

$$\mathcal{L}_{t}^{r}\left( {\tilde{\dot{\hat{\boldsymbol{y}}}}}_{t} \right)$$

This, as proven in Proof 1 and 2 is ensured to be minimized by taking $\boldsymbol{S}_{t}^{*}$ as a solution for (16.1) as,

$$\min\mathcal{L}_{t}^{r}\left( f\left( \boldsymbol{u}_{t},\boldsymbol{\theta,}\boldsymbol{S}_{t}^{*} \right) \right)$$
